# Metabolic fate and detectability of three novel fluoroprolintane psychostimulants: in vitro and in vivo characterization by hyphenated mass spectrometry

**DOI:** 10.1007/s00204-026-04462-4

**Published:** 2026-05-25

**Authors:** Lea Wagmann, Anica Herter, Michael B. Dybek, Adeboye Adejare, Jason Wallach, Simon D. Brandt, Markus R. Meyer

**Affiliations:** 1 Experimental and Clinical Toxicology and Pharmacology, Center for Molecular Signaling (PZMS), PharmaScienceHub (PSH), Saarland University, Kirrberger Str. / Geb. 48.5, 66421 Homburg, Germany; 2 Department of Pharmaceutical Sciences, Philadelphia College of Pharmacy, Saint Joseph’s University, Philadelphia, USA; 3 Alexander Shulgin Research Institute, Lafayette, CA USA

**Keywords:** New psychoactive substances, Stimulants, LC-HRMS/MS, Toxicokinetics, Metabolism, Urine screening

## Abstract

Amphetamine-type stimulants (ATS) represent a major segment of the global drugs of abuse market. Prolintane (1-(1-phenylpentan-2-yl)pyrrolidine), a substituted phenylethylamine ATS, was historically utilized as medication for several therapeutic indications. This study aimed to investigate the metabolic fate and urinary detectability of the three novel derivatives 2-, 3-, and 4-fluoroprolintane, which were recently shown to act as monoamine reuptake inhibitors. Liquid chromatography (LC)-high-resolution (HR) tandem mass spectrometry (MS/MS) was used for tentative identification of metabolites in rat urines (collected over a 24 h period following oral administration of 2 mg/kg fluoroprolintane isomer, each) or incubations with pooled human liver S9 fraction (1 h and 6 h, 25 µM fluoroprolintane isomer, each). Isozyme mapping was performed using individual incubations with 11 human phase I monooxygenases. The same rat urines were used for detectability studies using standard urine screening approaches (SUSA) by gas chromatography (GC)-mass spectrometry (MS), LC-ion trap MS, and LC-HRMS/MS. The fluoroprolintanes were extensively metabolized, with a total of 79 metabolites identified. Hydroxylations and subsequent oxidations were primarily mediated by CYP1A2, CYP2B6, CYP2C19, and CYP2D6, while glucuronidation and *O*-methylation were observed as main follow-up phase II reactions. All three SUSA allowed detection, primarily via metabolites. However, the high degree of metabolic overlap will make isomer differentiation challenging. These findings contribute to a comprehensive risk assessment and provide critical reference data for clinical and forensic laboratories to identify these substances in biological specimens.

**Supplementary Information:**

The online version contains supplementary material available at https://doi.org/10.1007/s00204-026-04462-4.

## Introduction

Amphetamine-type stimulants (ATS) continue to represent a major segment of the global drugs of abuse market. Recent international monitoring efforts highlight both the scale and the rapid evolution of ATS production, trafficking, and use. According to the latest World Drug Report published by the United Nations Office on Drugs and Crime (UNODC), the synthetic drug market has expanded sharply in recent years, with seizures of ATS reaching record levels in 2023 and comprising nearly half of all synthetic drug seizures worldwide (UNODC 2025). Complementary analyses from the European Union Drugs Agency (EUDA) further underscore the widespread availability of synthetic stimulants (EUDA 2025). A critical challenge within that landscape is the rapid proliferation of new psychoactive substances (NPS). This heterogeneous class of compounds is often intentionally designed to mimic the effects of controlled substances while circumventing international regulatory frameworks. Since 2008, the NPS market has been characterized by high structural turnover and record quantities were seized in Europe between 2020 and 2022, underscoring the scale of this public health threat (EUDA 2024). The integration of NPS into established ATS markets further complicates monitoring and public health responses, as structurally related stimulants may appear unexpectedly with limited toxicological and pharmacological data available. In addition, the investigation of such compounds can inform the development of novel chemical entities with improved therapeutic profiles suitable for various clinical indications.

**Fig. 1:**
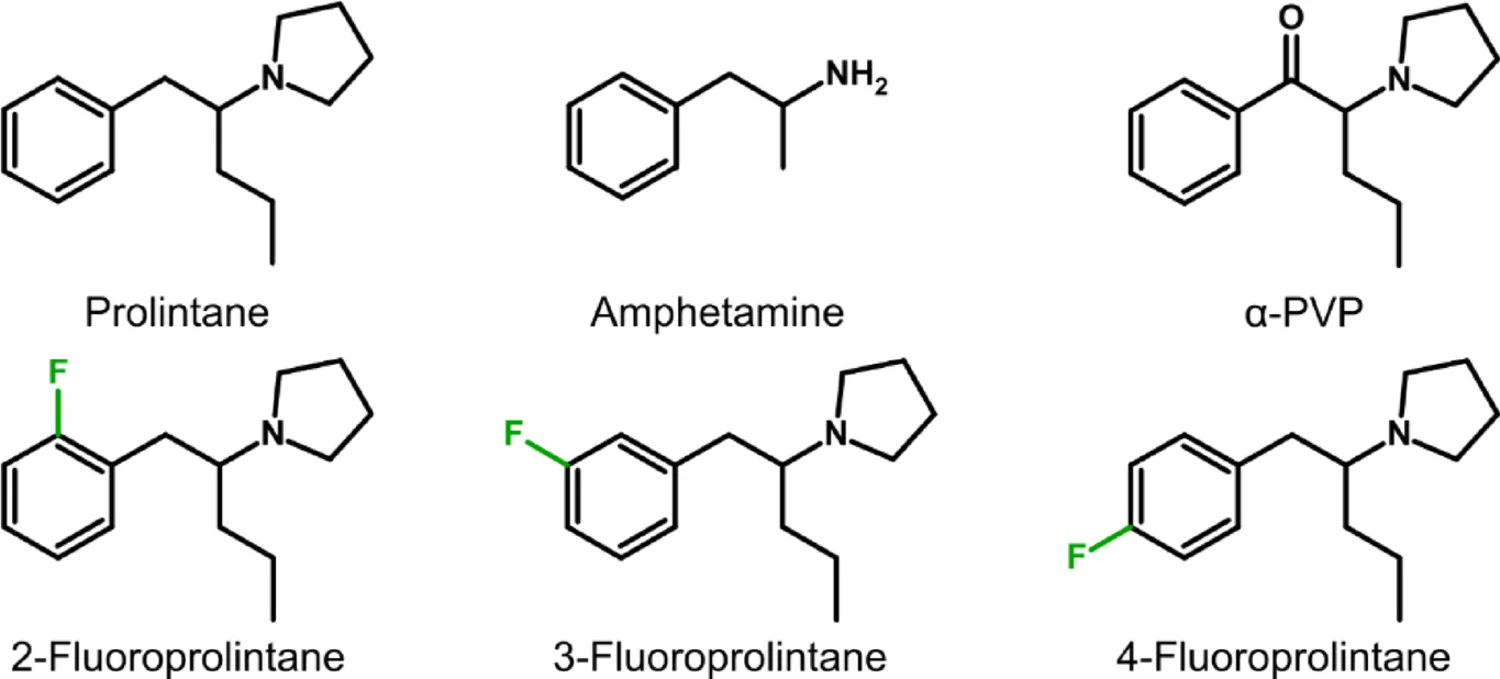
Chemical structures of prolintane, amphetamine, α-pyrrolidinovalerophenone (α-PVP), as well as 2-, 3-, and 4-fluoroprolintane

ATS and related NPS induce psychostimulation and euphoria by inhibiting monoamine reuptake transporters for dopamine (DAT), norepinephrine (NET), and serotonin (SERT), and/or promotion of transporter-mediated monoamine release (Simmler and Liechti 2018). Prolintane (1-(1-phenylpentan-2-yl)pyrrolidine, see Fig. 1) was developed as a pharmaceutical drug to promote wakefulness (Hollister and Gillespie 1970; Thomae 1959). However, adverse effects including headache, insomnia, anxiety, anorexia, hypertension, and tachycardia (Baselt 2020) as well as reports documenting its use for non-medical purposes or doping effectively led to the discontinuation of prolintane’s medical use in the 21st century (Delbeke 1996; Gaulier et al. 2002; Kyle and Daley 2007). Prolintane and structurally related compounds such as the NPS α-pyrrolidinovalerophenone (α-PVP, β-keto-prolintane, see Fig. 1) have been found to be potent uptake inhibitors at DAT and NET (Eshleman et al. 2019; Kastner et al. 2025). Recently, six ring-substituted prolintane analogs, including 2-, 3-, and 4-methyl and fluoro- positional isomers, were found to act as DAT and NET inhibitors and exhibited modest 5-HT release potencies (Kastner et al. 2025).

A defining feature of newly emerging NPS in contrast to pharmaceuticals is the absence of pre-market assessment including toxicity, pharmacokinetics, and metabolic fate. This lack of fundamental knowledge poses significant challenges not only to people who use drugs and healthcare providers during management of acute intoxications but also to central nervous system-focused medicinal chemists and clinical and forensic laboratories tasked with identifying these substances in biological specimens. Metabolism studies are a central pillar of drug development but also NPS risk assessment to elucidate biotransformation pathways, main metabolites, and elimination profiles, all of which are indispensable for interpreting toxicological findings, developing analytical screening methods, and assessing health risks (Pragst 2025). Such research is particularly important for novel ATS-type NPS, as small structural modifications can dramatically alter pharmacological activity, metabolic stability, and toxicity (EUDA 2024).

This study aimed to characterize the in vivo and in vitro metabolic fate and urinary detectability of the three novel ATS-type NPS 2-, 3-, and 4-fluoroprolintane (see Fig. 1). Metabolites were tentatively identified using liquid chromatography-high-resolution tandem mass spectrometry (LC-HRMS/MS) following oral administration in rats and incubation with pooled human liver S9 fractions (pHLS9). Additionally, phase I metabolic isozyme mapping was conducted using eleven human phase I monooxygenases. Finally, the detectability of these compounds and their metabolites including limits of identification (LOI) using standard urine screening approaches (SUSA) by gas chromatography (GC)-MS, LC-ion trap MS, and LC-HRMS/MS were assessed.

## Materials and methods

### Chemicals and enzymes

2-Fluoroprolintane hydrochloride, 3-fluoroprolintane hydrochloride, and 4-fluoroprolintane hydrochlorides were available from previous pharmacological studies (Kastner et al. 2025). Acetyl coenzyme A, isocitrate, isocitrate dehydrogenase, dithiothreitol, 3’-phosphoadenosine-5’-phosphosulfate (PAPS), *S*-(5’-adenosyl)-l-methionine (SAM), superoxide dismutase, reduced glutathione (GSH), potassium dihydrogen phosphate, dipotassium hydrogen phosphate, and Tris base were obtained from Sigma-Aldrich (Taufkirchen, Germany), trimipramine-d_3_ and diazepam-d_5_ from LGC (Wesel, Germany), and NADP^+^ from Biomol (Hamburg, Germany). Acetonitrile (LC-MS grade), ammonium formate (analytical grade), formic acid (LC-MS grade), methanol (LC-MS grade), and all other chemicals and reagents (analytical grade) were obtained from VWR (Darmstadt, Germany).

The baculovirus-infected insect cell microsomes (Supersomes) containing the human complementary cDNA-expressed cytochrome P450 (CYP) enzymes CYP1A2, CYP2A6, CYP2B6, CYP2C8, CYP2C9, CYP2C19, CYP2D6, CYP3A4 (1 nmol/mL, each), CYP2E1, CYP3A5 (2 nmol/mL, each), flavin-containing monooxygenase 3 (FMO3, 5 mg protein/mL), as well as pooled human liver microsomes (pHLM, 35 donors, 20 mg microsomal protein/mL, 240 pmol total CYP/mg protein), pHLS9 (10 donors, 20 mg microsomal protein/mL), uridine 5’-diphospho (UDP)-glucuronosyltransferase (UGT) reaction mixture solution A (25 mM UDP-glucuronic acid), and UGT reaction mixture solution B (250 mM Tris-HCl, 40 mM MgCl_2_, and 0.125 mg/mL alamethicin) were obtained from Discovery Life Sciences (Huntsville, LA, USA). After delivery the enzyme preparations and PAPS were thawed at 37 °C, aliquoted, snap-frozen in liquid nitrogen, and stored at -80 °C until use.

### Rat urine collection and sample preparation for identification of metabolites

The animal experiments, which were carried out in accordance with German legislation, the Animal Welfare Act, and the EU Directive 2010/63, have been approved by an ethics committee (Landesamt für Verbraucherschutz, Saarbrücken, Germany, 33/2019). Three male Wistar rats (Charles River, Sulzfeld, Germany), not showing any signs of discomfort, pain, or illness, were used for metabolism experiments according to previous studies (Frankenfeld et al. 2024; Wagmann et al. 2019a). The rats were three months old and treated with one of the fluoroprolintanes, orally administered in an aqueous suspension by gastric intubation in doses of 2 mg/kg body weight, which was between 300 and 330 g. Prior to compound administration, blank urine was collected to serve as a negative control and establish the absence of interfering compounds. Rats were housed in metabolism cages for 24 h with water ad libitum. Accumulated urine was collected at 24 h and was free of feces. Rats were carefully monitored after administration and well-being was assessed based on a previously established scoresheet. No adverse reactions were observed during the course of the experiment.

According to previous studies (Frankenfeld et al. 2024; Wagmann et al. 2019a), two different sample preparation procedures were performed: simple urine precipitation for the identification of phase I and II metabolites or enzymatic conjugate cleavage followed by liquid-liquid extraction (LLE) for the exclusive and more sensitive identification of phase I metabolites.

For urine precipitation, 0.5 mL of acetonitrile containing 0.2 mg/L of diazepam-d_5_ as internal standard (IS) were added to 0.1 mL of rat urine. The mixture was shaken on a rotary shaker for 2 min at 2,000 rpm. After centrifugation for 2 min at 18,407 × g, 0.5 mL of the mixture was transferred into heated glass vial at 70 °C and evaporated to dryness under a gentle stream of nitrogen. The resulting residue was dissolved in 50 µL of water: acetonitrile (1:1; *v/v*) mixture containing 0.1% formic acid and 5 mM ammonium formate before analysis by reversed-phase LC-HRMS/MS.

For conjugate cleavage followed by LLE, 2 mL of rat urine (adjusted to pH 5.2 with 50 µL of acetic acid, 1 M) were incubated at 56 °C for 3 h with 50 µL of a glucuronidase (EC no. 3.2.1.31, Merck) and arylsulfatase (EC no. 3.1.6.1, Merck) mixture from *Helix pomatia*. Afterwards, the mixture was acidified with 1 mL of hydrochloric acid (37%), and then 2 mL of ammonium sulfate solution (30%) and 1 mL of sodium hydroxide solution (10 M) were added (final pH 8–9), as well as 0.1 mL of methanol containing 0.1 mg/mL diazepam-d_5_ (IS). This mixture was then extracted with 5 mL of ethyl acetate/dichloromethane/isopropanol (3:1:1, *v/v/v*), followed by manually shaking for 20 s and centrifugation for two minutes at 3000 × g to separate the organic from the aqueous phase. The organic layer was then transferred into a heated glass flask at 60 °C, evaporated to dryness, and reconstituted with 0.1 mL of methanol.

### pHLS9 Incubations for identification of metabolites

The procedure was carried out as previously described (Wagmann et al. 2019a) Briefly, the final incubation volume was 150 µL and all given concentrations are final concentrations in the incubation mixture. First, a mixture containing 25 µg/mL alamethicin (UGT reaction mixture solution B), 90 mM phosphate buffer (pH 7.4), 2.5 mM Mg^2+^ (as MgCl), 2.5 mM isocitrate, 0.6 mM NADP^+^, 0.8 U/mL isocitrate dehydrogenase, 100 U/mL superoxide dismutase, 0.1 mM acetyl coenzyme A, and 2 mg/mL pHLS9 was preincubated for 10 min at 37 °C. Afterwards, 2.5 mM UDP glucuronic acid (UGT reaction mix solution A), 40 µM PAPS, 1.2 mM SAM, 1 mM dithiothreitol, and 10 mM GSH were added. The reactions were then initiated by addition of 25 µM of one of the fluoroprolintane isomers, each, in the above-described phosphate buffer and the tube was incubated for 360 min at 37 °C. After the initial 60 min, 60 µL of this incubation mixture was transferred to another tube and the reaction terminated by addition of 20 µL ice-cold acetonitrile containing 2.5 µM of trimipramine-d_3_ (IS). The remaining mixture (90 µL) was incubated for the full 360 min and then stopped via the addition of 30 µL ice-cold acetonitrile containing 2.5 µM of trimipramine-d_3_ (IS). After the reaction was terminated all solutions were cooled for 30 min at -20 °C, centrifuged for 2 min at 18,407 x g, and 50 µL of the supernatants were transferred to autosampler vials and analyzed by LC-HRMS/MS. Blank incubations without the fluoroprolintane isomers and control samples without pHLS9 were prepared to confirm the absence of interfering compounds and to identify compounds that are not of metabolic origin, respectively. All incubations were performed in duplicates.

### Monooxygenases activity screening

In accordance with a previous study (Wagmann et al. 2019a), microsomal incubations were performed at 37 °C for 30 min with 25 µM of the fluoroprolintane isomer, each, and 50 pmol/mL CYP isoenzyme (CYP1A2, CYP2A6, CYP2B6, CYP2C8, CYP2C9, CYP2C19, CYP2D6, CYP2E1, CYP3A4, or CYP3A5) or 0.25 mg protein/mL FMO3 (Wagmann et al. 2019a). The incubation mixtures with a final volume of 50 µL also contained 90 mM phosphate buffer (pH 7.4), the NADP regenerating system (1.2 mM NADP^+^, 5 mM Mg^2+^, 5 mM isocitrate, 0.5 U/mL isocitrate dehydrogenase), and 200 U/mL superoxide dismutase. The concentrations reported are final concentrations. All incubations were performed with phosphate buffer except for those with CYP2A6 and CYP2C9 which were performed with 90 mM Tris buffer (pH 7.4) in accordance with the manufacturer’s recommendations. An incubation with 1 mg protein/mL pHLM and a blank incubation without enzyme were also performed. The reactions were initiated by addition of the NADP regenerating system and terminated by addition of 50 µL ice-cold acetonitrile containing 2.5 µM of trimipramine-d_3_ (IS). Afterwards the mixture was centrifuged for 5 min at 18,407 x g and 50 µL of the supernatant were transferred to an autosampler vial and analyzed by LC-HRMS/MS.

### High resolution tandem mass spectrometry (HRMS/MS) and data evaluation conditions

Analyses were performed using a Thermo Fisher Scientific (TF, Dreieich, Germany) Dionex UltiMate 3000 Rapid Separation (RS) UHPLC system with a quaternary UltiMate 3000 RS pump and an UltiMate 3000 RS autosampler controlled by the TF Chromeleon software version 6.80 coupled to a TF Q-Exactive Plus, equipped with a heated electrospray ionization II source (HESI II). The HESI-II conditions were already described previously (Wagmann et al. 2019a): heater temperature, 320 °C; ion transfer capillary temperature, 320 °C; sheath gas, 60 arbitrary units (AU); auxiliary gas, 10 AU; spray voltage, 4.00 kV, positive mode; S-lens RF level, 50.0. The mass spectrometer was mass calibrated before analysis using a Positive Mode Cal Mix (Supelco, Bellefonte, PA, US).

LC conditions for tentative identification of metabolites in rat urines, pHLS9 incubations, and the monooxygenase activity screening were as follows: TF Accucore PhenylHexyl column (100 mm x 2.1 mm inside diameter, ID, 2.6 μm particle size) at 40 °C maintained by a Dionex UltiMate 3000 RS analytical column heater; gradient elution with eluent A (2 mM aqueous ammonium formate solution containing 0.1% (*v/v*) formic acid) and eluent B (2 mM ammonium formate solution with acetonitrile/methanol (50:50, *v/v*) containing 0.1% (*v/v*) formic acid and 1% (*v/v*) water). The following gradient and settings were: 0–1.0 min hold 1% B, 1.0–9.5 min to 70% B, 9.5–12.0 min hold 99% B, 12.0–13.5 min hold 1% B with curve 5 for all steps. The flow rate settings were as follows: 0–9.5 min at 0.5 mL/min and 9.5–13.5 min at 0.8 mL/min. The injection volume was 5 µL for all samples. The mass spectrometer operated in full scan and subsequent data-dependent MS^2^ (dd‐MS^2^) mode with an inclusion list containing mass-to-charge ratios *m/z* (M + H^+^) of the fluoroprolintane isomers (*m/z* 236.1809) and expected metabolites. The settings for full scan data acquisition were chosen as follows: resolution, 35,000; automatic gain control (AGC) target, 1e^6^; maximum injection time (IT), 120 ms; scan range, *m/z* 100–600. The settings for the dd-MS^2^ mode were chosen as follows: resolution, 17,500; AGC target, 2e^5^; maximum IT, 250 ms; loop count, 5; isolation window, *m/z* 1.0; stepped normalized collision energy, 17.5%, 35%, 52.5%; pick others, enabled. TF Xcalibur Qual Browser software version 4.1.31.9 was used for data collection and evaluation.

### Toxicological detectability in rat urine

SUSA by GC-MS, LC-ion trap MS, and LC‐HRMS/MS were performed as described previously (Frankenfeld et al. 2024; Helfer et al. 2015; Maurer et al. 2023; Wagmann et al. 2019a; Wissenbach et al. 2011), which included LLE after partial acidic hydrolysis followed by acetylation for the GC-MS SUSA and urine precipitation with acetonitrile for the LC‐based SUSA.

### Limits of identification in rat urine (LOI)

LOI were determined in accordance with a previous publication (Frankenfeld et al. 2024) and instruments and settings were the same as described for SUSA above. For GC-MS analysis the determinations were conducted as follows: a volume of 4.5 mL of blank rat urine was fortified with 500 µL of 2-, 3-, or 4-fluoroprolintane in methanol to achieve final urine concentrations of 20 mg/L, 10 mg/L, 1 mg/L, or 0.1 mg/L. Spiked urine samples (*n* = 3) were extracted by LLE after partial urine hydrolysis followed by acetylation (U+UHyAc), which corresponded to the GC-MS SUSA sample preparation (Maurer et al. 2023). Briefly, samples were divided into two aliquots of 2.5 mL, one aliquot was mixed with 0.1 mL of IS solution (0.1 mg/mL diazepam-d_5_ in methanol), and 1 mL of hydrochloric acid (37%) was added. This mixture was hydrolyzed for 15 min under reflux and 2 mL of ammonium sulfate solution (30%) and 1 mL of sodium hydroxide solution (10 M) were added (final pH 8–9). Then, the second aliquot of 2.5 mL of untreated urine was added in accordance with the GC-MS SUSA sample preparation. This mixture was then extracted with 5 mL of ethyl acetate/ dichloromethane/isopropanol (3:1:1, *v/v/v*), followed by manual shaking for 20 s and centrifugation for two minutes at 3000 × g to separate the organic from the aqueous phase. The organic layer was then transferred into a heated glass flask at 60 °C and evaporated to dryness. Samples were then derivatized with a mixture of acetic aldehyde and pyridine (3:2, *v/v*) using microwave irradiation (580 W, 5 min). After the acetylation, extracts were evaporated to dryness at 60 °C, reconstituted with 0.1 mL of methanol, and a 1 µL aliquot of each sample was injected onto the GC-MS system. The criteria for the LOI were fulfilled when the compound could be identified based on its MS spectrum using an extended version of the Maurer/Meyer/Pfleger/Weber GC-MS mass spectral library of drugs, poisons, and their metabolites (Maurer et al. 2023).

LOI of the LC-ion trap MS and LC-HRMS/MS analyses, were determined as follows. A volume of 90 µL of blank rat urine (*n* = 3) was fortified with 10 µL of 2-, 3-, or 4-fluoroprolintane in methanol to achieve final urine concentrations of 1000 µg/L, 100 µg/L, 10 µg/L, or 1 µg/L. Spiked urine samples were extracted via urine precipitation, as described above, which corresponded to the LC-ion trap MS and LC-HRMS/MS SUSA sample preparations. A 10 µL aliquot each was injected onto the LC-ion trap MS system. The criteria for the LOI were fulfilled when the S/N was at least 3 and the compound could be identified via its MS^2^ spectra using an extended version of the Maurer/Wissenbach/Weber library (Maurer et al. 2019). A 5 µL aliquot each was injected onto the LC-HRMS/MS system. The criteria for the LOI were fulfilled when the S/N was at least 3 and the compound could be identified via its MS^2^ spectra using an extended version of the Maurer/Meyer/Helfer/Weber library (Maurer et al. 2025).

## Results and discussion

### Identification of metabolites

Given that reference material was unavailable for the metabolites, the proposed identifications are tentative being based on HRMS^2^ spectra and retention times. Furthermore, the exact positions of the metabolic transformations on a given moiety, e.g., hydroxylation of the phenyl ring, cannot be determined with certainty from the fragment ion (FI) patterns. To tentatively identify metabolites, MS^1^ data after analysis of the in vivo and in vitro samples were manually mined for exact precursor masses (PM) of expected metabolites. A maximum deviation of 5 ppm between measured and calculated exact PM was considered acceptable. Afterwards, the fragmentation patterns in their HRMS^2^ spectra were individually interpreted and compared to those of the parent compounds. The metabolites were sorted by increasing mass and, in the case of isomers, by increasing retention time. Each metabolite was assigned to a unique metabolite ID, e.g. 4FM-1 meaning metabolite 1 of 4-fluoroprolintane. Full spectra of the parent compounds and all phase I and II metabolites detected are included in the Supplementary Figures S1–S3 (2-, 3-, and 4-fluoroprolintane, respectively, see Electronic Supplementary Material). The conditions and duration of the chromatographic run of 13.5 min allowed for sufficient separation of the parent compound and its metabolites. However, this setting was unable to chromatographically separate the parent isomers and is therefore not recommended for this purpose (see Supplementary Figure S4 in the Electronic Supplementary Material). A method to separate the parent isomers is available from previous studies (Kastner et al. 2025).

**Fig. 2:**
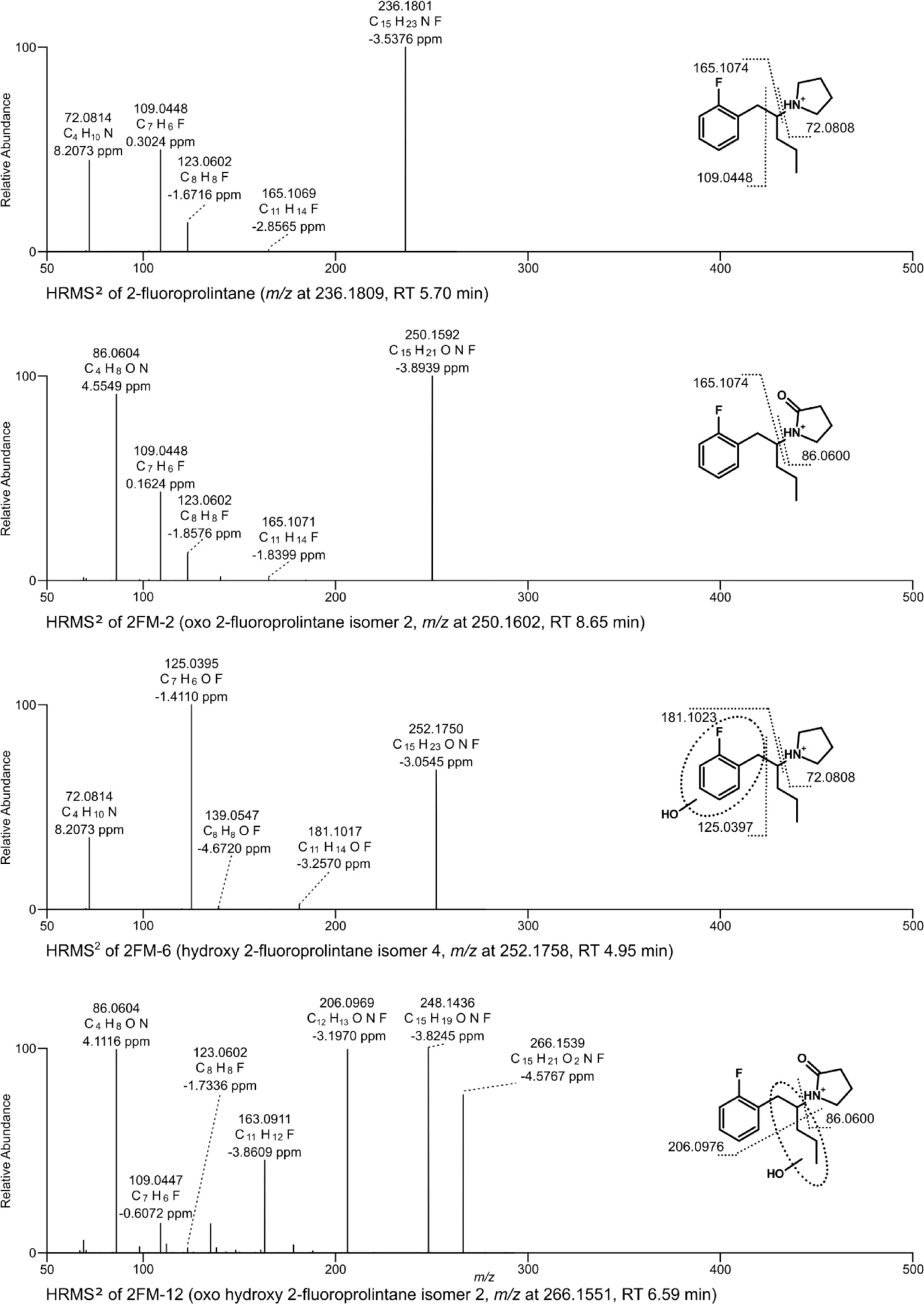

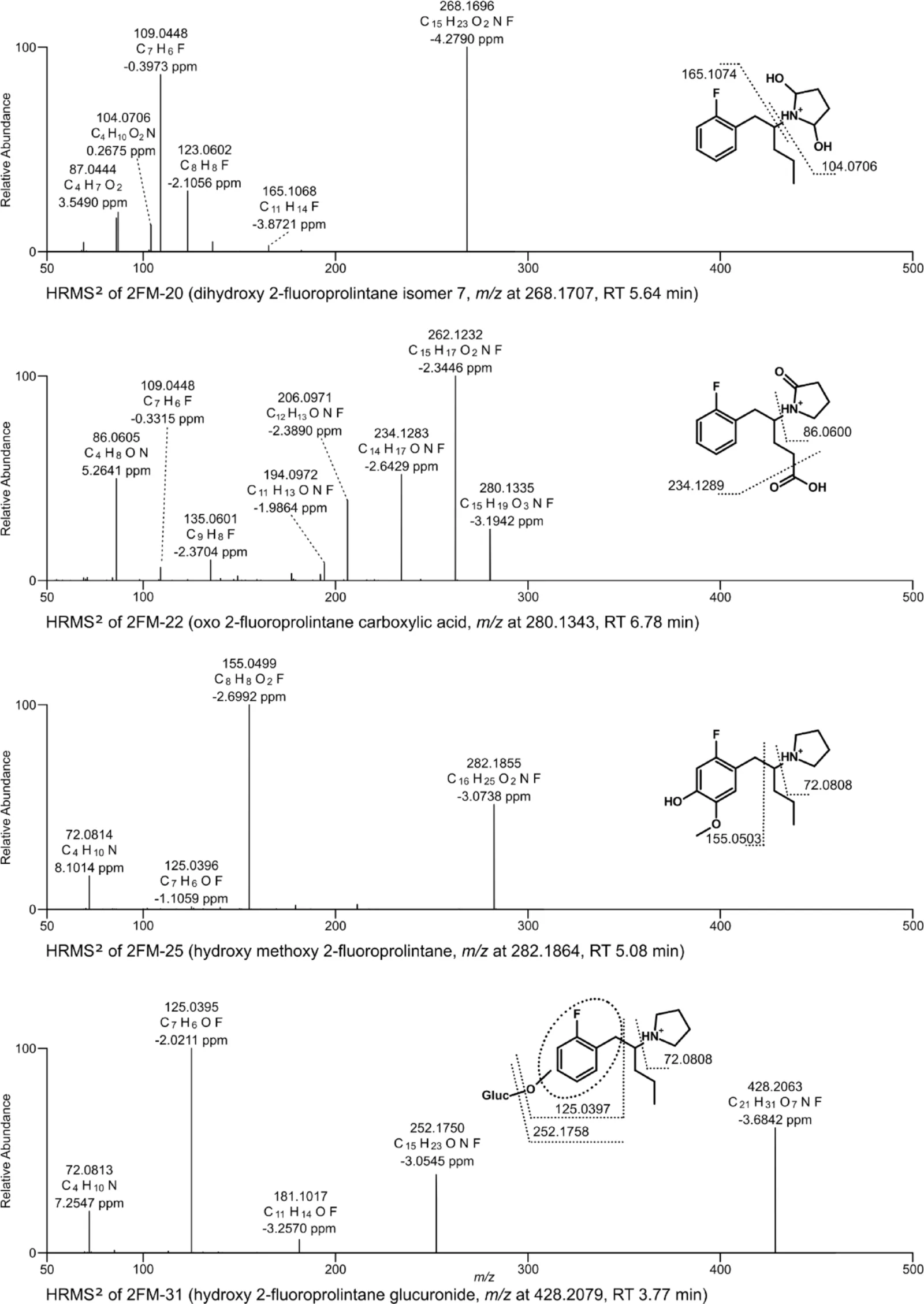
High-resolution (HR) MS^2^ spectra and retention times (RT) of 2-fluoroprolintane and selected metabolites detected in vivo and/or in vitro

Spectra of 2-fluoroprolintane and those of phase I and II metabolites with the highest abundance in rat urine and/or pHLS9 incubations are presented and discussed in the following section and are summarized in Fig. 2. Only calculated exact masses are given in the text. The analyses of rat urine and in vitro incubations with pHLS9, pHLM, or individual monooxygenases resulted in detections of numerous tentative metabolites for each isomer: 33 metabolites of 2-fluoroprolintane, 22 metabolites of 3-fluoroprolintane; and 24 metabolites of 4-fluoroprolintane.

Fragmentation patterns of 2-fluoroprolintane and selected metabolites are discussed below in some detail as representative examples, since the data for 3-fluoroprolintane, 4-fluoroprolintane, and their metabolites followed similar pathways. The HRMS^2^ spectrum of the parent compound 2-fluoroprolintane (M + H^+^, PM, at *m/z* 236.1809, C_15_H_23_NF^+^, see Fig. 2) showed the elimination of the pyrrolidine ring (-71 u, C_4_H_9_N, FI, at *m/z* 165.1074, C_11_H_14_F^+^). Furthermore, both a propylene (-42 u, C_3_H_6_, FI at *m/z* 123.0605, C_8_H_8_F^+^) and butylene moieties were eliminated (-56 u, C_4_H_8_, FI at *m/z* 109.0448, C_7_H_6_F^+^). The FI at *m/z* 72.0808 (C_4_H_10_N^+^) corresponded to the protonated pyrrolidine ring. For the metabolites, an additional oxygen atom and loss of two hydrogens led to the increased PM of 2FM-2 (+ 14 u, M + H^+^, PM at *m/z* 250.1602, C_15_H_21_ONF^+^) indicating the presence of an oxo group and likely the formation of a lactam pyrrolidin-2-one ring. The FI at *m/z* 165.1074 (C_11_H_14_F^+^), 123.0605 (C_8_H_8_F^+^), and 109.0448 (C_7_H_6_F^+^), already described for the parent compound, were also present in the spectrum of 2FM-2. However, the FI at *m/z* 72.0808 increased by + 14 u resulting in the FI at *m/z* 86.0600 (C_4_H_8_ON^+^), consistent with the γ-butyrolactam (pyrrolidin-2-one) most probably formed via initial metabolic hydroxylation and subsequent oxidation. In the case of 2FM-2, the oxo group was suggested to be in 2-position of the pyrrolidine ring due to the enhanced retention time of the metabolite, presumably due to higher lipophilicity from loss of the basic amine nitrogen. This observation aligns well with the fact that lactam formation was previously described as prevalent biotransformation for pyrrolidine containing drugs and the behavior of pyrrolidinophenone metabolites separated under reversed-phase LC conditions seen previously (Bolleddula et al. 2014; Manier et al. 2019; Maurer et al. 2025; Wagmann et al. 2019b).

The addition of a single oxygen atom to the parent molecule was detected as an increased PM of 2FM-6 (+ 16 u, M + H^+^, PM at *m/z* 252.1758, C_15_H_23_ONF^+^). In this case, the pyrrolidine ring represented by the FI at *m/z* 72.0808 remained unchanged, but the other FI described for the parent compound increased by + 16 u and resulted in the FI at *m/z* 181.1023 (C_11_H_14_OF^+^), 139.0554 (C_8_H_8_OF^+^), and 125.0397 (C_7_H_6_OF^+^), consistent with hydroxylation at the benzylic position or the fluorophenyl ring. Unfortunately, the final position of the hydroxy group could not be determined based on the fragmentation pattern. The PM and fragmentation pattern of 2FM-12 (+ 30 u, M + H^+^, PM at *m/z* 266.1551, C_15_H_21_O_2_NF^+^) indicated the presence of both an oxo group and a hydroxy group. The detection of FI at *m/z* 86.0600 (C_4_H_8_ON^+^), coupled with the observation that the metabolite eluted later than the parent compound, again suggest the same pyrrolidin-2-one moiety proposed above for 2FM-2. Furthermore, the elimination of water (-18 u, H_2_O, FI at *m/z* 248.1445, C_15_H_19_ONF^+^), together with the detection of the FI at *m/z* 163.0918 (C_11_H_12_F^+^), i.e. FI at *m/z* 165.1074 in the spectrum of the parent compound decreased by -2 u, indicated that hydroxylation may have occurred on the alkyl side chain. Correspondingly, the fragment ions at *m/z* 123.0605 (C_8_H_8_F^+^) and 109.0448 (C_7_H_6_F^+^) were also observed in the mass spectra of the parent compound and 2FM-12.

The spectrum of 2FM-20 (+ 32 u, M + H^+^, PM at *m/z* 268.1707, C_15_H_23_O_2_NF^+^), indicated the addition of two oxygen atoms, containing the FI at *m/z* 104.0706 (C_4_H_10_O_2_N^+^), while the FI at *m/z* 165.1074 (C_11_H_14_F^+^), 123.0605 (C_8_H_8_F^+^), and 109.0448 (C_7_H_6_F^+^), already described for the parent compound, were also present. Both hydroxyl groups were therefore expected to be located on the pyrrolidine ring. This was supported by the subsequent loss of ammonia (-17 u, NH_3_) by this FI, resulting in the FI at *m/z* 87.0441 (C_4_H_7_O_2_^+^), consistent with fragmentation mechanisms observed with pyrrolidinophenone metabolites reported previously (Manier et al. 2019). Retention time and mass spectral data recorded for 2FM-22 (+ 44 u, M + H^+^, PM at *m/z* 280.1343, C_15_H_19_O_3_NF^+^) suggested γ-lactam formation (FI at *m/z* 86.0600, C_4_H_8_ON^+^) similar to 2FM-2 as well as the presence of a carboxylic group on the end of the alpha-alkyl side chain. The latter addition is supported by the loss of formic acid (-46 u, CH_2_O_2_) resulting in the FI at *m/z* 234.1289 (C_14_H_17_ONF^+^) and the presence of the FI at *m/z* 109.0448 (C_7_H_6_F^+^) indicating the presence of an unchanged fluorophenyl ring.

The presence of an additional oxygen atom and a methoxy group are consistent with the increased PM seen in 2FM-25 (+ 46 u, M + H^+^, PM at *m/z* 282.1864, C_16_H_25_O_2_NF^+^) and the increased mass shift of the FI from *m/z* 109.0448 (characteristic C_7_H_6_F^+^ tropylium ion, representing the parent molecule without additional phenyl ring substitutions) to *m/z* 155.0503 (C_8_H_8_O_2_F^+^), suggesting these metabolic changes are on the fluorophenyl ring. In the case of 2FM-31, hydroxylation and glucuronidation are consistent with the increased PM (+ 192 u, M + H^+^, PM at *m/z* 428.2079, C_21_H_31_O_7_NF^+^). After elimination of the glucuronic acid moiety (-176 u, FI at *m/z* 252.1758, C_15_H_23_ONF^+^), the remaining fragment ions are consistent with those observed in the mass spectrum of hydroxylated metabolite 2FM-6.

### Metabolic pathways and isozyme mapping

**Fig. 3:**
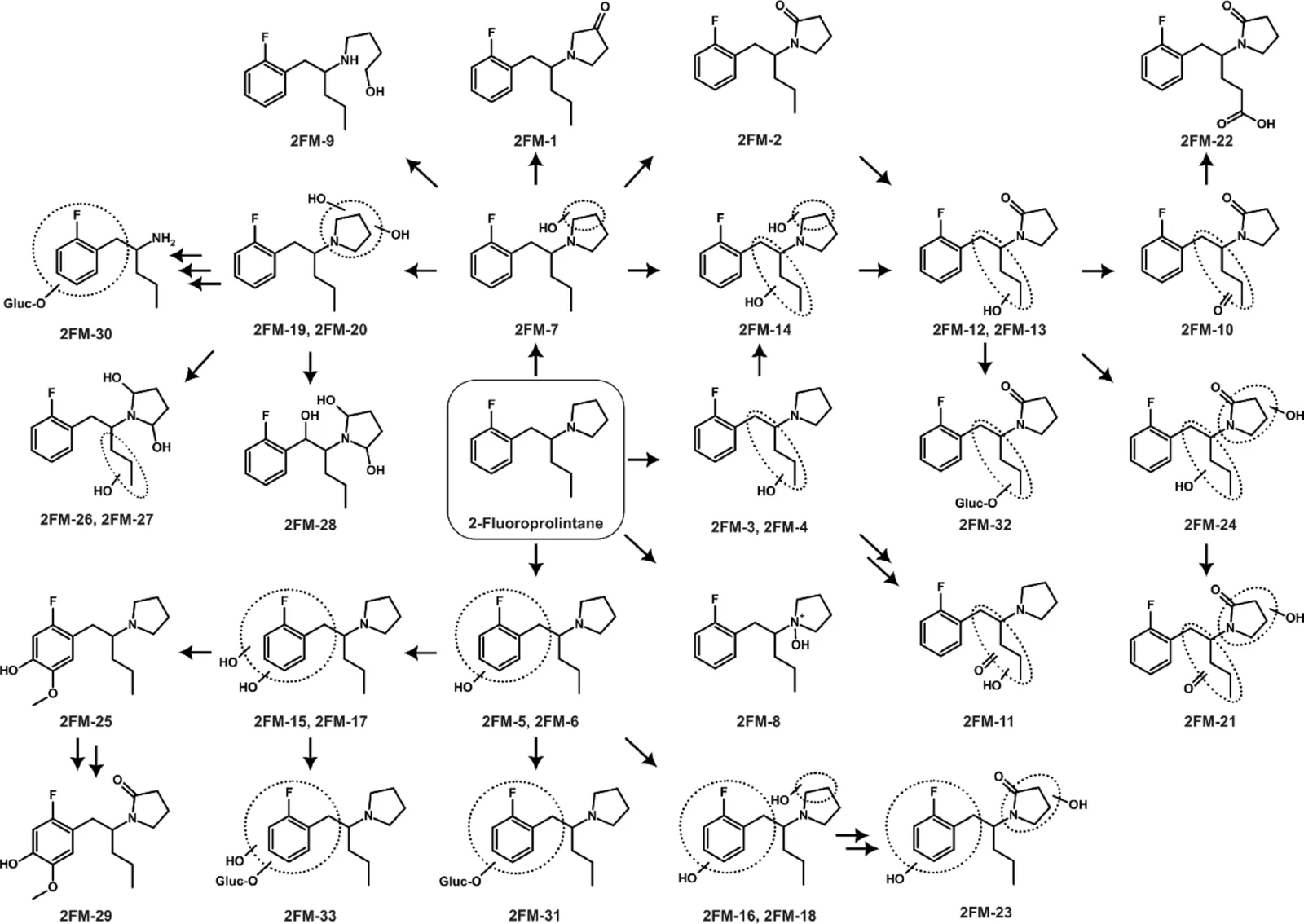
In vivo and in vitro metabolic pathways of 2-fluoroprolintane

**Fig. 4:**
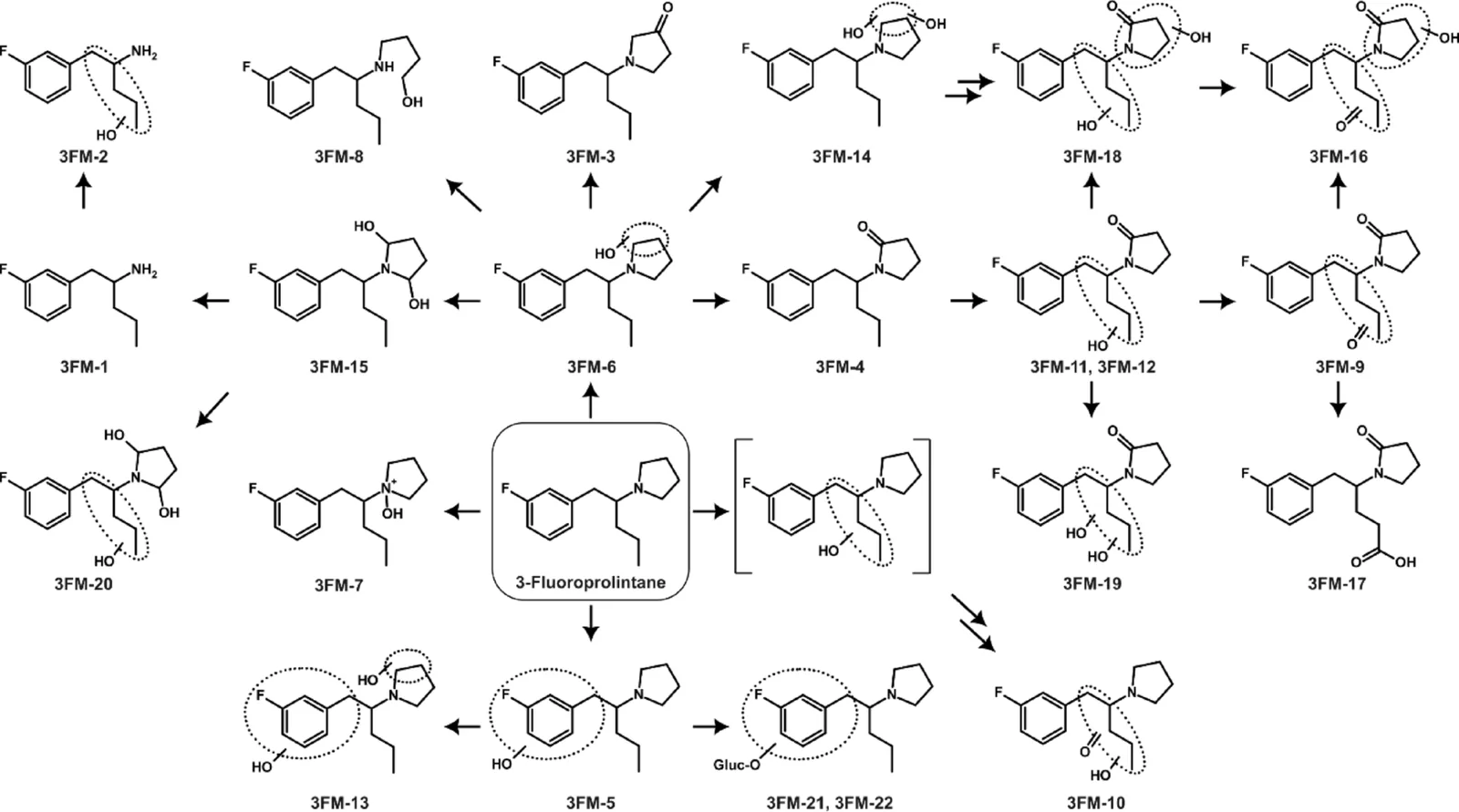
In vivo and in vitro metabolic pathways of 3-fluoroprolintane

**Fig. 5:**
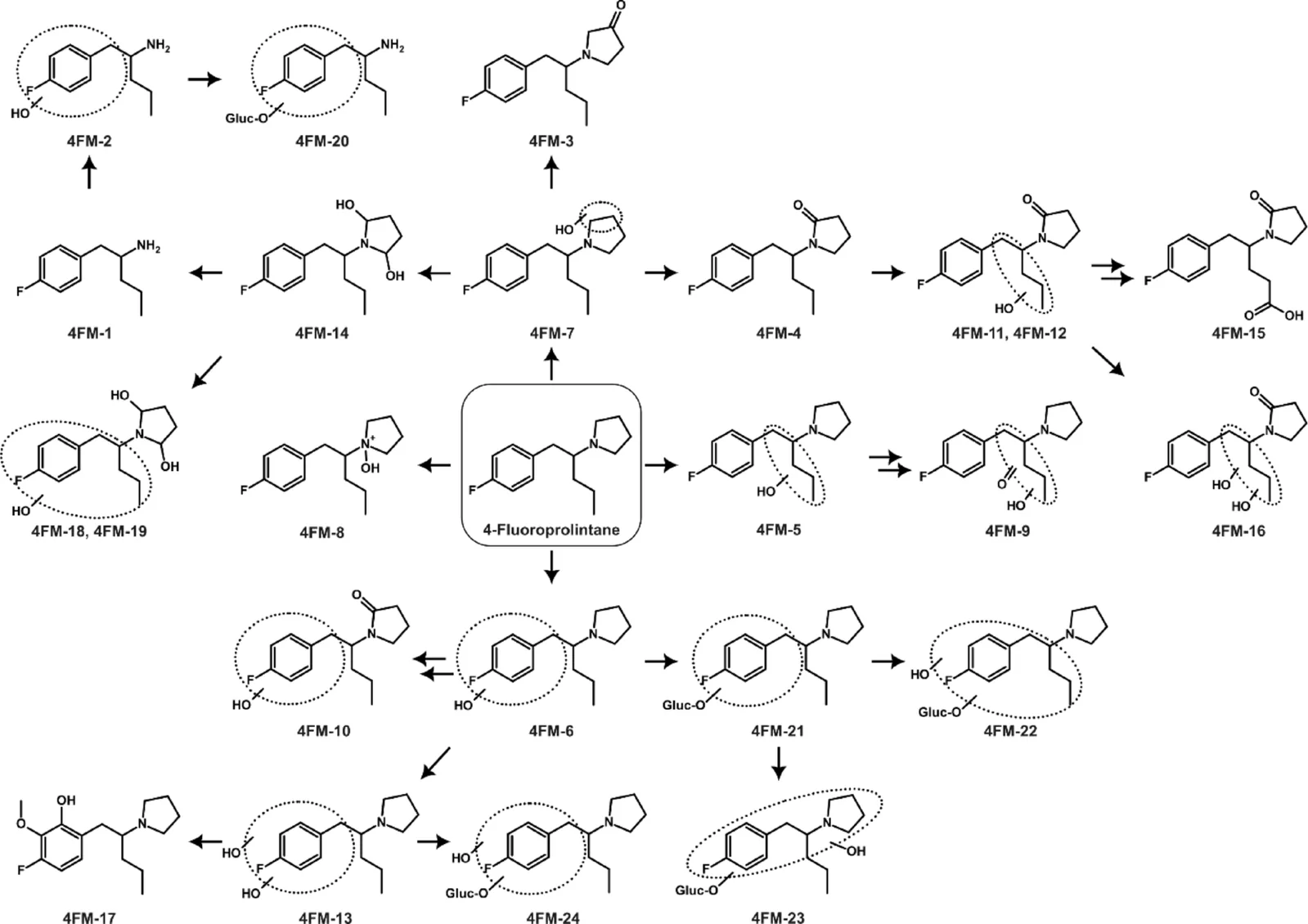
In vivo and in vitro metabolic pathways of 4-fluoroprolintane

All proposed in vivo and in vitro metabolic pathways are summarized in Figs. 3, 4 and 5. Additionally, the monooxygenases activity screening was performed to investigate the impact of 10 CYP isoenzymes and FMO3 on the initial phase I metabolic transformations of the three fluoroprolintane isomers. The results are summarized in Table 1. Incubations with pHLM were additionally performed for comparative purposes. Since pHLM represent a mixture of all microsomal liver enzymes, it would be expected that all metabolites detected in the single-enzyme incubations would also be observed in the pHLM incubations. Nevertheless, several metabolites were detected in the single-isozyme incubations but not in the pHLM incubations (3FM-3, 3FM-7, 4FM-3, and 4FM-8). This can be explained by the fact that recombinant single-enzyme systems are enriched in one CYP isoform known to exhibit higher enzyme activity compared to pHLM (Venkatakrishnan et al. 2000). Phase I metabolic transformations observed with 2-fluoroprolintane (see Fig. 3) included five mono-hydroxylations (2FM-3 to 2FM-7); 2FM-3 and 2FM-4 were hydroxylated on the alkyl side chain, 2FM-5 and 2FM-6 were hydroxylated on the benzylic position or the fluorophenyl ring, and 2FM-7 was hydroxylated on the pyrrolidine ring. Presumably, subsequent oxidation of hydroxy groups on the pyrrolidine ring provides the oxo metabolites 2FM-1 and 2FM-2. All of these metabolic transformations were found to be catalyzed by CYP1A2, CYP2C19, or CYP2D6. CYP2B6 and CYP3A4 only catalyzed a subset of these metabolic reactions, whereas FMO3 catalyzed the *N*-oxygenation of 2-fluoroprolintane to give the *N*-oxide metabolite 2FM-8. In the case of 2FM-9, the pyrrolidine ring underwent ring opening comparable to previous reports on pyrrolidinophenones (Manier et al. 2019; Sauer et al. 2009). Combinations of two or three hydroxylations led to seven dihydroxy (2FM-14 to 2FM-20) and three trihydroxy isomers (2FM-26 to 2FM-29). Further oxidations of these metabolites formed several combined metabolites containing oxo or carboxyl groups. In total, three oxo hydroxy isomers (2FM-11 to 2FM-13), two oxo dihydroxy isomers (2FM-23 and 2FM-24), as well as dioxo (2FM-10), dioxo hydroxy (2FM-21), and oxo carboxylic acid metabolites (2FM-22) were identified. The phase I metabolic transformations observed were largely consistent with those expected from monooxygenase enzymes.

**Table 1:** General involvement of monooxygenases in the formation of the given 2-, 3-, and 4-fluoroprolintane phase I metabolites

Parent compound	CYP										FMO	pHLM
Metabolite ID	1A2	2A6	2B6	2C8	2C9	2C19	2D6	2E1	3A4	3A5	3	
*2-Fluoroprolintane*	+	+	+	+	+	+	+	+	+	+	+	+
2FM-1 (oxo-)	-	-	+	-	-	+	+	-	-	-	-	+
2FM-2 (oxo-)	+	-	-	-	-	+	-	-	-	-	-	+
2FM-3 (hydroxy-)	+	-	-	-	-	+	+	-	-	-	-	+
2FM-4 (hydroxy-)	-	-	+	-	-	+	-	-	+	-	-	+
2FM-5 (hydroxy-)	-	-	-	-	-	+	+	-	-	-	-	+
2FM-6 (hydroxy-)	+	-	-	-	-	+	+	-	-	-	-	+
2FM-7 (hydroxy-)	+	-	+	-	-	+	+	-	+	-	-	+
2FM-8 (*N*-oxide)	-	-	-	-	-	-	-	-	-	-	+	+
*3-Fluoroprolintane*	+	+	+	+	+	+	+	+	+	+	+	+
3FM-3 (oxo-)	-	-	-	-	-	-	+	-	-	-	-	-
3FM-4 (oxo-)	+	-	+	-	-	-	+	-	+	-	-	+
3FM-5 (hydroxy-)	+	-	+	-	-	+	+	-	-	-	-	+
3FM-6 (hydroxy-)	+	-	+	-	-	+	+	-	-	-	-	+
3FM-7 (*N*-oxide)	-	-	-	-	-	-	-	-	-	-	+	-
*4-Fluoroprolintane*	+	+	+	+	+	+	+	+	+	+	+	+
4FM-3 (oxo-)	-	-	-	-	-	-	+	-	-	-	-	-
4FM-4 (oxo-)	+	-	-	-	-	-	-	-	+	-	-	+
4FM-5 (hydroxy-)	-	-	+	-	-	-	-	-	-	-	-	+
4FM-6 (hydroxy-)	-	-	-	-	-	-	-	-	-	-	-	-
4FM-7 (hydroxy-)	+	-	+	-	-	+	+	-	-	-	-	+
4FM-8 (*N*-oxide)	-	-	-	-	-	-	+	-	-	-	+	-

Pooled human liver microsomes (pHLM) incubations were performed additionally. Metabolite IDs correspond to Figs. 3, 4 and 5. CYP, cytochrome P450; FMO, flavin-containing monooxygenase, +, detected in in vitro incubations; -, not detected in in vitro incubations

Phase II metabolic transformations of phase I metabolites included UGT-catalyzed glucuronidation (2FM-30 to 2FM-33) and catechol-*O*-methyl transferase (COMT)-catalyzed formation of hydroxy methoxy metabolites (2FM-25 and 2FM-29). The formation of 2FM-30 included the transformation of the pyrrolidine ring to the corresponding primary amine, which was observed previously with structurally related pyrrolidinophenones (Manier et al. 2019; Sauer et al. 2009). Two isoforms of the COMT are known, a membrane-bound form and a soluble cytoplasmic form. The latter has been reported to play a more prominent role in the inactivation of catechol-containing xenobiotics (Bastos et al. 2017). As COMT-catalyzed transfer of a methyl group requires a 1,2-dihydroxy-phenyl catechol moiety, positions 4 and 5 of the 2-fluorobenzyl ring are considered likely sites for the dihydroxylation of 2-fluoroprolintane, due to the ring electronic and steric effects. Furthermore, hydrophobic amino acids in proximity to the active site of COMT can influence the regioselectivity of the transformation, resulting in preferential methylation at the meta-position relative to the alkyl side chain in phenylalkylamine substrates. This regioselectivity has been described for many phenylalkylamine COMT substrates (Cao et al. 2014; Schwaninger et al. 2011; Tsao et al. 2011).

In case of 3-fluoroprolintane, only two hydroxylations (3FM-5 and 3FM-6) were identified as initial phase I metabolic transformations (see Fig. 4). 3FM-5 was hydroxylated either on the benzylic or fluorophenyl ring position and 3FM-6 suggested hydroxylation of the pyrrolidine ring. Oxidation of hydroxy groups in 2- or 1-position of the pyrrolidine ring presumably led to the oxo metabolites 3FM-3 and 3FM-4, respectively. These metabolic transformations were all found to be catalyzed by CYP1A2, CYP2B6, or CYP2D6. Whereas CYP2C19 and CYP3A4 catalyzed only a subset of these metabolic reactions. As with 2-fluoroprolintane, *N*-oxygenation of 3-fluoroprolintane (3FM-7) was catalyzed by FMO3. The formation of 3FM-1 and 3FM-2 included the elimination of the pyrrolidine ring resulting in the corresponding primary amine. In the case of 3FM-8 the pyrrolidine ring underwent ring opening. Three dihydroxy isomers (3FM-13 to 3FM-15) were also identified, along with three oxo hydroxy isomers (3FM-10 to 3FM-12), two oxo dihydroxy isomers (3FM-18 and 3FM-19), as well as single trihydroxy (3FM-20), dioxo (3FM-9), dioxo hydroxy (3FM-16), and oxo carboxylic acid metabolites (3FM-17). Phase II metabolic steps included UGT-catalyzed glucuronidation of 3FM-5 (3FM-21 and 3FM-22).

For 4-fluoroprolintane (see Fig. 5), three mono-hydroxylations (4FM-5 to 4FM-7) were detected. 4FM-5 was hydroxylated at the side chain, 4FM-6 at the benzylic position or at the fluorophenyl ring, and 4FM-7 at the pyrrolidine ring. Subsequent oxidation of hydroxy groups on the pyrrolidine ring led to the oxo metabolites 4FM-3 and 4FM-4. Whereas 4FM-6 was only detected in rat urine, all other metabolic steps were catalyzed by CYP1A2, CYP2B6, or CYP2D6. CYP2C19 and CYP3A4 catalyzed only a subset of these metabolic reactions. CYP2D6 and FMO3 were found to catalyze the *N*-oxygenation of 4-fluoroprolintane (4FM-8). In the cases of 4FM-1 and 4FM-2, the pyrrolidine ring underwent transformation to the corresponding primary amine. Two dihydroxy isomers (4FM-13 and 4FM-14) were identified, along with four oxo hydroxy isomers (4FM-9 to 4FM-12), two trihydroxy isomers (4FM-18 and 4FM-19), as well as single oxo dihydroxy (4FM-16), and oxo carboxylic acid metabolites (4FM-15). Phase II metabolic steps included COMT-catalyzed formation of a hydroxy methoxy metabolite (4FM-17) and UGT-catalyzed glucuronidations of hydroxy metabolites (4FM-20 to 4FM-24).

Overall, the in vivo and in vitro metabolic pathways of 2-, 3-, and 4-fluoroprolintane were comparable, with only minor differences observed. Furthermore, the metabolic steps observed for the fluoroprolintanes were similar to those reported previously for prolintane as well as structurally similar pyrrolidinophenones including α-PVP (Baselt 2020; Manier et al. 2019; Sauer et al. 2009). Though the metabolic transformations were not quantified, the fact that several CYP isozymes were found to be involved in the initial transformations of the phase I metabolism of 2-, 3-, and 4-fluoroprolintane may suggest a reduced risk of toxicity due to interindividual activity differences of single CYP isozymes or hampered metabolism and elimination due to e.g., CYP inhibition in the context of drug-drug or drug-food interactions. Moreover, the involvement of metabolic enzymes from many different families, including CYP, FMO, COMT, and UGT support a similar conclusion.

**Fig. 6:**
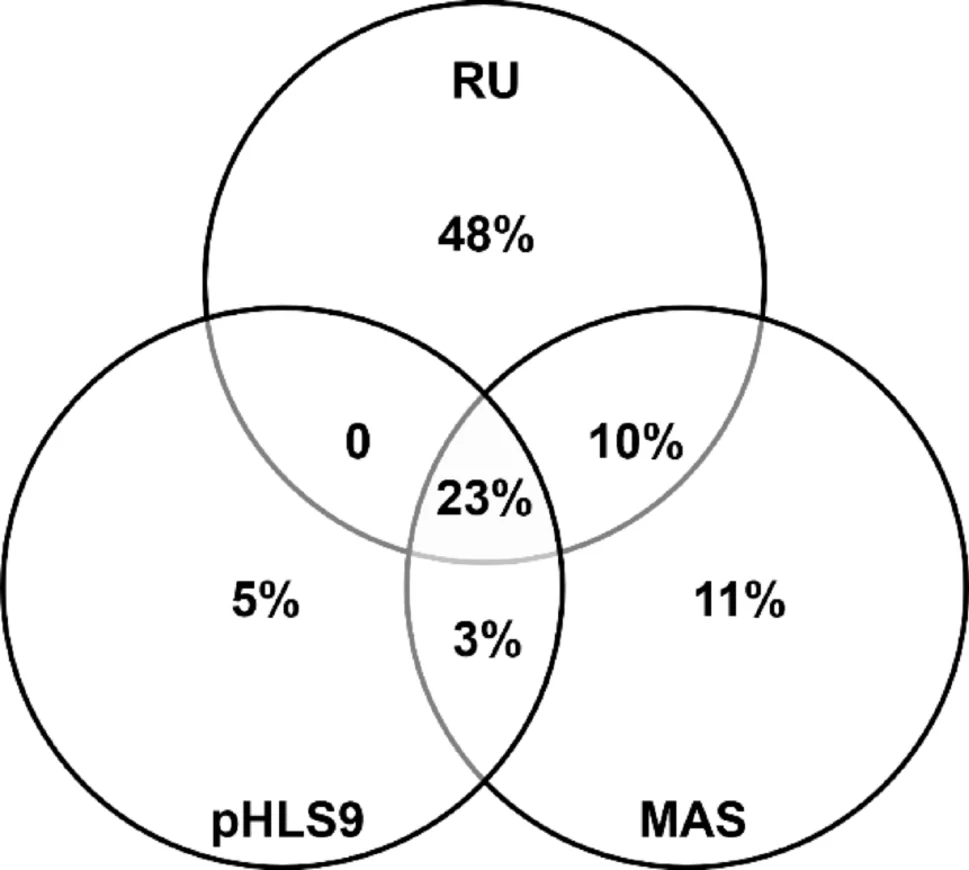
Relative amount of 2-, 3-, and 4-fluoroprolintane metabolites detected in the given metabolism models. RU, rat urine; pHLS9, pooled human liver S9 fraction incubation; MAS, monooxygenases activity screening; 100% represent 79 metabolites tentatively identified in total

Table S1 (see Electronic Supplementary Material) provides a summary of metabolites detected in rat urine, pHLS9 incubations, and/or the monooxygenases activity screening. In total, a greater number of metabolites were tentatively identified from rat urine (65 metabolites) compared to the in vitro incubations (41 metabolites). The relative amount of 2-, 3-, and 4-fluoroprolintane metabolites detected in the given metabolism models is depicted in Fig. 6. In total, 79 metabolites of 2-, 3-, and 4-fluoroprolintane were tentatively identified. 48% of this total metabolite number were exclusively detected in rat urine, whereas only 5% and 11% were exclusively detected in pHLS9 incubations and the monooxygenases activity screening, respectively. 23% of the tentatively identified metabolites were detected in all three metabolism models, while 10% could be detected in rat urine and the monooxygenases activity screening, but not in the pHLS9 incubations.

These variations were likely caused by differences in the experimental conditions. In vitro experiments are widely acknowledged to have limitations concerning distribution and excretion processes leading to production of more simple metabolites involving only a few transformation steps. In addition, incubations during the monooxygenases activity screening were stopped after 30 min, whereas the pHLS9 incubations were stopped after a maximum of 6 h, and rat rat urine was collected over a 24 h period. It is therefore not surprising that more complex metabolites were detected in rat urine, including many phase II metabolites detected exclusively in rat urine. Although rats are the most frequently used mammalian model for identifying urinary biomarkers of drugs of abuse and NPS, it is also important to consider species differences that may lead to the formation of different metabolites or metabolite ratios. Despite potential species differences combining both in vivo and in vitro approaches is expected to provide a comprehensive metabolic overview, suitable for the development of clinical and toxicological screening procedures (Wagmann et al. 2019a).

### Toxicological detectability in rat urine

Application of the dose-by-factor approach allows for the calculation of the human-equivalent dose from the rat dose (Sharma and McNeill 2009). The rat dose of 2 mg/kg body weight results in a human-equivalent dose of about 20 mg if a body weight of 60 kg was assumed. No human doses for fluoroprolintane isomers were described, but 10 to 30 mg were described as common oral human single doses of prolintane (Baselt 2020). Therefore, human doses might be expected to be in this range, whereas doses resulting in severe intoxications are expected to be higher.

To the best of the authors’ knowledge, neither 2-, 3-, nor 4-fluoroprolintane were detected on the drugs of abuse market so far and human biosamples were not available. However, one lesson learned from the NPS phenomenon is that one cannot exclude the appearance of these compounds on the market especially due to their reported pharmacological activity and ability to inhibit DAT, NET, and SERT, typical for other psychostimulants (Kastner et al. 2025). A proactive approach is therefore considered critical for providing comprehensive information and data used for risk assessments related to clinical and forensic casework. These findings might also be relevant in drug discovery efforts in this space to find better tolerated psychostimulant medications with reduced abuse liability. An intake by humans in the framework of a controlled trial would be the gold standard for the identification of metabolites in blood and urine, but is usually not feasible in the context of NPS due to ethical concerns and lacking knowledge of toxicity, toxicokinetics, and toxicodynamics (Wagmann et al. 2019b). Human biosamples following ingestion of NPS derived from authentic cases are sometimes available and can then be used for confirmation of those metabolites previously identified in vivo (e.g., rats) and/or in vitro.

Rat urine after oral administration of the fluoroprolintane isomers could not only be used to tentatively identify their metabolites but also to simulate the routine analysis of a urine sample in a clinical or forensic toxicological context following the intake of unknown compounds. For this purpose, SUSA by GC-MS, LC-ion trap MS, and LC-HRMS/MS were applied to the rat urine samples. These screening approaches were developed to detect a broad spectrum of compounds such as poisons, therapeutics, drugs of abuse, and their metabolites in urine specimens, thereby providing information on prior substance consumption. Because these methods are designed to detect numerous different compounds, their chromatographic and mass spectrometric settings are not specifically optimized for the fluoroprolintane isomers, and, unlike in the targeted experiments for metabolite identification, no inclusion list is used in the LC-HRMS/MS SUSA. Furthermore, information on the limits of identification (LOI) obtained from all three SUSAs by spiking blank rat urine provided valuable insights into the ability to detect the parent compound in urine following the ingestion of biorelevant doses of the fluoroprolintane isomers.

### Toxicological detectability using GC-MS SUSA

**Fig. 7:**
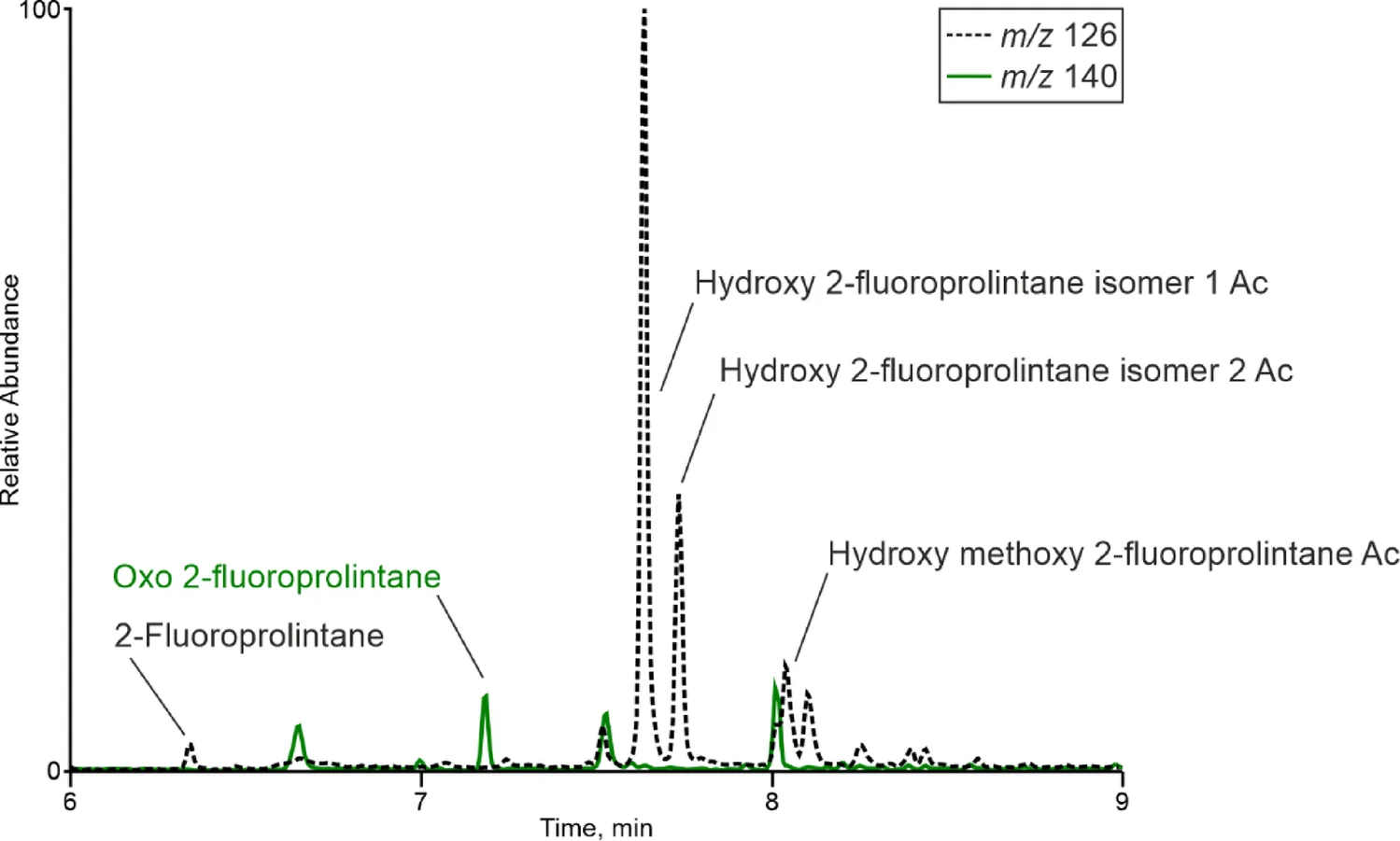
Selected mass chromatograms of the GC-MS standard urine screening approach including liquid-liquid extraction after partial acidic hydrolysis followed by acetylation of a rat urine sample after oral administration of 2-fluoroprolintane (2 mg/kg body weight)

Using U+UHyAc with GC-MS, the LOI were determined to be at 100 µg/L for all parent compounds. Detectability of the fluoroprolintane isomers using the GC-MS SUSA is summarized in Table 2 and selected mass chromatograms of the GC-MS SUSA of the rat urine sample after oral administration of 2-fluoroprolintane are depicted in Fig. 7. U+UHyAc with GC-MS allowed for the detection of the three parent compounds in rat urine following oral administration of 2 mg/kg body weight. Furthermore, metabolites of each fluoroprolintane isomer could successfully be detected. The 2-oxo-pyrrolidine metabolites could be detected for all compounds. In the cases of 2-fluoroprolintane and 4-fluoroprolintane, two acetylated hydroxy isomers were detected for each test drug. All hydroxylations were most probably located at the fluorophenyl ring due to the presence of fragment *m/z* 126 indicating an unchanged side chain and pyrroline ring. For 2-fluoroprolintane, the acetylated hydroxy methoxy metabolite was also detected. For this reason, oxo and hydroxy metabolites are recommended as additional urine screening targets when employing GC-MS analysis.

**Table 2:** Toxicological detectability of 2-, 3-, and 4-fluoroprolintane in rat urine after oral administration of 2 mg/kg body weight by the GC-MS standard urine screening approach

Compound	Precursor ion mass, m/z	Elemental composition	Retention index (RI)	Characteristic fragment Ions, m/z
2-Fluoroprolintane	235	C_15_H_22_NF	1710	96, 109, 126, 192, 234
2-Fluoroprolintane-M (oxo)	249	C_15_H_20_ONF	1940	98, 109, 140, 206, 249
2-Fluoroprolintane-M (hydroxy isomer 1) Ac	293	C_17_H_24_O_2_NF	2060	96, 126, 208, 250, 292
2-Fluoroprolintane-M (hydroxy isomer 2) Ac	293	C_17_H_24_O_2_NF	2090	96, 126, 208, 250, 292
2-Fluoroprolintane-M (hydroxy methoxy) Ac	323	C_18_H_26_O_3_NF	2170	126, 155, 280, 322
3-Fluoroprolintane	235	C_15_H_22_NF	1720	96, 109, 126, 192, 234
3-Fluoroprolintane-M (oxo)	249	C_15_H_20_ONF	1960	98, 109, 140, 206, 249
4-Fluoroprolintane	235	C_15_H_22_NF	1725	96, 109, 126, 192, 234
4-Fluoroprolintane-M (oxo)	249	C_15_H_20_ONF	1970	98, 109, 140, 206, 249
4-Fluoroprolintane-M (hydroxy isomer 1) Ac	293	C_17_H_24_O_2_NF	2060	96, 126, 208, 250, 292
4-Fluoroprolintane-M (hydroxy isomer 2) Ac	293	C_17_H_24_O_2_NF	2090	96, 126, 208, 250, 292

+, detected; −, not detected; AC, acetylated

### Toxicological detectability using LC-ion trap MS SUSA

Using urine precipitation with LC-ion trap MS, the LOI were determined to be at 100 µg/L for all parent compounds. Detectability of the fluoroprolintane isomers using the LC-ion trap MS SUSA is summarized in Table 3. All parent compounds were successfully detected after urine precipitation and LC-ion trap MS analysis. Eight metabolites of 2-fluoroprolintane were detected, consisting of five phase I and three phase II metabolites. Phase I metabolites ranged from single (monohydroxylated, 2FM-6) to three step transformation (trihydroxylated, 2FM-27) metabolites. As the sample preparation did not involve intentional conjugate cleavage, two intact glucuronides were detected and serve as additional screening targets. Comparable results were obtained for 4-fluoroprolintane with six phase I and three phase II metabolites, including two intact glucuronides. For 3-fluoroprolintane, only five phase I metabolites but no phase II metabolites were detected. All in all, hydroxy (mono-, bis- and trihydroxylations), oxo carboxylic acid metabolites, and hydroxy glucuronides are therefore recommended as additional urine screening targets for LC-ion trap MS analysis.

**Table 3:** Toxicological detectability of 2-, 3-, and 4-fluoroprolintane in rat urine after oral administration of 2 mg/kg body weight by the LC-ion trap MS standard urine screening approach

Parent compound or metabolite ID	Precursor ion mass, m/z	Retention time, min	Characteristic MS^2^ fragment ions, m/z (relative intensity)
2-Fluoroprolintane	236	10.10	72(64), 109(96), 123(100), 165(5)
2FM-6 (hydroxy-)	252	7.44	72(5), 125(100), 139(1), 181(8)
2FM-20 (dihydroxy-)	268	9.42	87(22), 104(32), 109(79), 123(100), 165(16), 182(8)
2FM-22 (oxo carboxylic acid-)	280	10.83	86(39), 135(6), 159(1), 177(8), 234(100), 244(3)
2FM-24 (oxo dihydroxy-)	282	8.41	86(9), 102(6), 109(8), 206(100), 222(41)
2FM-25 (hydroxy methoxy-)	282	7.85	126(1), 155(100), 179(6), 211(2)
2FM-27 (trihydroxy-)	284	5.77	87(22), 104(41), 109(21), 135(9), 163(100), 206(2), 224(1)
2FM-31 (hydroxy glucuronide-)	428	4.10	125(17), 181(3), 252(100)
2FM-33 (dihydroxy glucuronide-)	444	3.92	126(1), 141(23), 197(2), 268(100)
3-Fluoroprolintane	236	10.37	72(23), 109(85), 123(100), 165(11)
3FM-7 (*N*-oxide)	252	11.25	88(29), 109(54), 123(100), 165(38)
3FM-10 (oxo hydroxy-)	266	5.99	109(3), 135(100), 177(14), 195(4)
3FM-15 (dihydroxy-)	268	9.74	87(13), 104(24), 109(73), 123(100), 165(30), 182(3)
3FM-17 (oxo carboxylic acid-)	280	10.91	86(50), 135(22), 159(17), 177(26), 234(100), 244(18)
3FM-20 (trihydroxy-)	284	6.85	87(5), 104(12), 109(20), 135(10), 163(100), 206(24), 224(67)
4-Fluoroprolintane	236	10.28	72(64), 109(96), 123(100), 165(5)
4FM-6 (hydroxy-)	252	8.04	72(5), 125(100), 139(4), 181(7)
4FM-8 (*N*-oxide)	252	11.17	88(100), 109(30), 123(51), 165(12)
4FM-13 (dihydroxy-)	268	5.75	125(7), 126(85), 141(100), 197(5)
4FM-14 (dihydroxy-)	268	9.65	87(33), 104(40), 109(78), 123(100), 165(13), 182(9)
4FM-15 (oxo carboxylic acid-)	280	11.18	86(35), 135(3), 159(100), 177(42), 234(64), 244(99)
4FM-16 (oxo dihydroxy-)	282	8.54	86(7), 109(4), 135(9), 161(8), 206(100), 222(1), 246(10)
4FM-17 (hydroxy methoxy-)	282	7.05	126(45), 155(100), 179(9), 211(6)
4FM-21 (hydroxy glucuronide-)	428	4.25	125(8), 181(1), 252(100)
4FM-24 (dihydroxy glucuronide-)	444	3.84	126(3), 141(8), 197(1), 268(100)

Relative intensities are given in brackets. Metabolite iDs correspond to Figs. 3, 4 and 5

### Toxicological detectability using LC-HRMS/MS SUSA

Using urine precipitation with LC-HRMS/MS operating in full scan and subsequent dd-MS^2^ mode without an inclusion list, the LOI were determined to be at 100 µg/L for all parent compounds except for 2-fluoroprolintane (1000 µg/L). Detectability of the fluoroprolintane isomers using the LC-HRMS/MS SUSA is summarized in Table 4. In contrast to the other two SUSA, the parent compounds were not detected after urine precipitation with LC-HRMS/MS analysis. This was most probably caused by the lower injection volume (5 µL for LC-HRMS/MS versus 10 µL for LC-ion trap MS) in combination with the shorter duration of the chromatographic runs (13.5 min for LC-HRMS/MS versus over 20 min for LC-ion trap MS). In accordance with the LC-ion trap MS-based SUSA, eight metabolites of 2-fluoroprolintane were detected, comprising five phase I and three phase II metabolites, including a hydroxy methoxy metabolite and two glucuronides. Phase I metabolites ranged from single (monohydroxylated, 2FM-6) to three transformation (trihydroxylated, 2FM-27) metabolites. For 3-fluoroprolintane, only six phase I metabolites were detected, but no phase II metabolites. For 4-fluoroprolintane, five phase I and two phase II metabolites were detected. Overall, hydroxy (mono-, bis- and trihydroxylations), oxo carboxylic acid metabolites, and hydroxy glucuronides are therefore recommended as additional urine screening targets for LC-HRMS/MS analysis.

**Table 4:** Toxicological detectability of 2-, 3-, and 4-fluoroprolintane in rat urine after oral administration of 2 mg/kg body weight by the LC-HRMS/MS standard urine screening approach

Metabolite ID	Exact precursor ion mass, m/z	Retention time, min
2FM-6 (hydroxy-)	252.1758	4.30
2FM-20 (dihydroxy-)	268.1707	4.89
2FM-22 (oxo carboxylic acid-)	280.1343	5.71
2FM-24 (oxo dihydroxy-)	282.1500	5.03
2FM-25 (hydroxy methoxy-)	282.1864	4.35
2FM-27 (trihydroxy-)	284.1656	4.06
2FM-31 (hydroxy glucuronide-)	428.2079	3.37
2FM-33 (dihydroxy glucuronide-)	444.2028	3.28
3FM-7 (*N*-oxide)	252.1758	5.23
3FM-10 (oxo hydroxy-)	266.1551	3.83
3FM-15 (dihydroxy-)	268.1707	4.92
3FM-17 (oxo carboxylic acid-)	280.1343	5.69
3FM-19 (oxo dihydroxy-)	282.1500	5.05
3FM-20 (trihydroxy-)	284.1656	4.09
4FM-6 (hydroxy-)	252.1758	4.03
4FM-8 (*N*-oxide)	252.1758	5.23
4FM-13 (dihydroxy-)	268.1707	3.70
4FM-15 (oxo carboxylic acid-)	280.1343	5.69
4FM-16 (oxo dihydroxy-)	282.1500	5.03
4FM-21 (hydroxy glucuronide-)	428.2079	3.18
4FM-24 (dihydroxy glucuronide-)	444.2028	3.26

Metabolite iDs correspond to Figs. 3, 4 and 5

## Conclusions

The global ATS market is undergoing rapid expansion, accompanied by the ongoing emergence of structurally diverse NPS. These developments present major challenges for public health, law enforcement, and clinical and forensic toxicology, due to the lack of pre-market safety testing and limited availability of metabolic data for newly appearing substances. This proactive study addressed this critical knowledge gap by providing the first comprehensive characterization of the metabolic fate and urinary detectability of the three novel psychostimulants 2-, 3-, and 4-fluoroprolintane. This supports clinical and forensic toxicologists, informs toxicological risk assessments, and enhances the understanding of the health implications associated with these emerging psychoactive compounds. This work also provides insight into the metabolic profile of three fluoroprolintane isomers that may inform medicinal chemists and pharmacologists investigating novel psychostimulants with improved tolerability and reduced abuse liability.

The research revealed that 2-, 3-, and 4-fluoroprolintane were extensively metabolized, with 79 total metabolites tentatively identified across rat urine and in vitro incubations. Hydroxylation was the predominant phase I biotransformation, mainly catalyzed by CYP1A2, CYP2B6, CYP2C19, and CYP2D6, while subsequent *O-*methylation and glucuronidation represented the major phase II metabolic pathways. Contribution of several isozymes involved in the metabolism may suggest a decreased likelihood of toxicity arising from CYP-mediated drug-drug interactions or inter-individual genetic polymorphisms in metabolic enzymes although additional studies are required. The study showed that SUSA by GC-MS, LC-ion trap MS, and LC-HRMS/MS should be able to identify a case of fluoroprolintane ingestion. Although overlapping metabolites complicate differentiation of single isomers, the identification of common biomarkers allows reliable confirmation of exposure. Thus, law enforcements and toxicologists were provided with essential information for risk assessment and the effective monitoring of these emerging psychoactive compounds.

## Supplementary Information

Below is the link to the electronic supplementary material.

Supplementary Material 1

## Data Availability

The data that support the findings of this study are available from the corresponding author upon reasonable request.
