# Supplementary material for: Metabolic fate and detectability of three novel fluoroprolintane psychostimulants: in vitro and in vivo characterization by hyphenated mass spectrometry
Source: Arch Toxicol. 2026 May 25;100(10):4561–78. doi: 10.1007/s00204-026-04462-4 (PMC13593737; doi:10.1007/s00204-026-04462-4)
Supplement: Supplementary file 1 [file 204_2026_4462_MOESM1_ESM.pdf]

Electronic Supplementary Materials

Metabolic fate and detectability of three novel fluoroprolintane psychostimulants: In vitro and in vivo characterization by hyphenated mass spectrometry

Lea Wagmann, Anica Herter, Michael B. Dybek, Adeboye Adejare, Jason Wallach, Simon D. Brandt, Markus R. Meyer

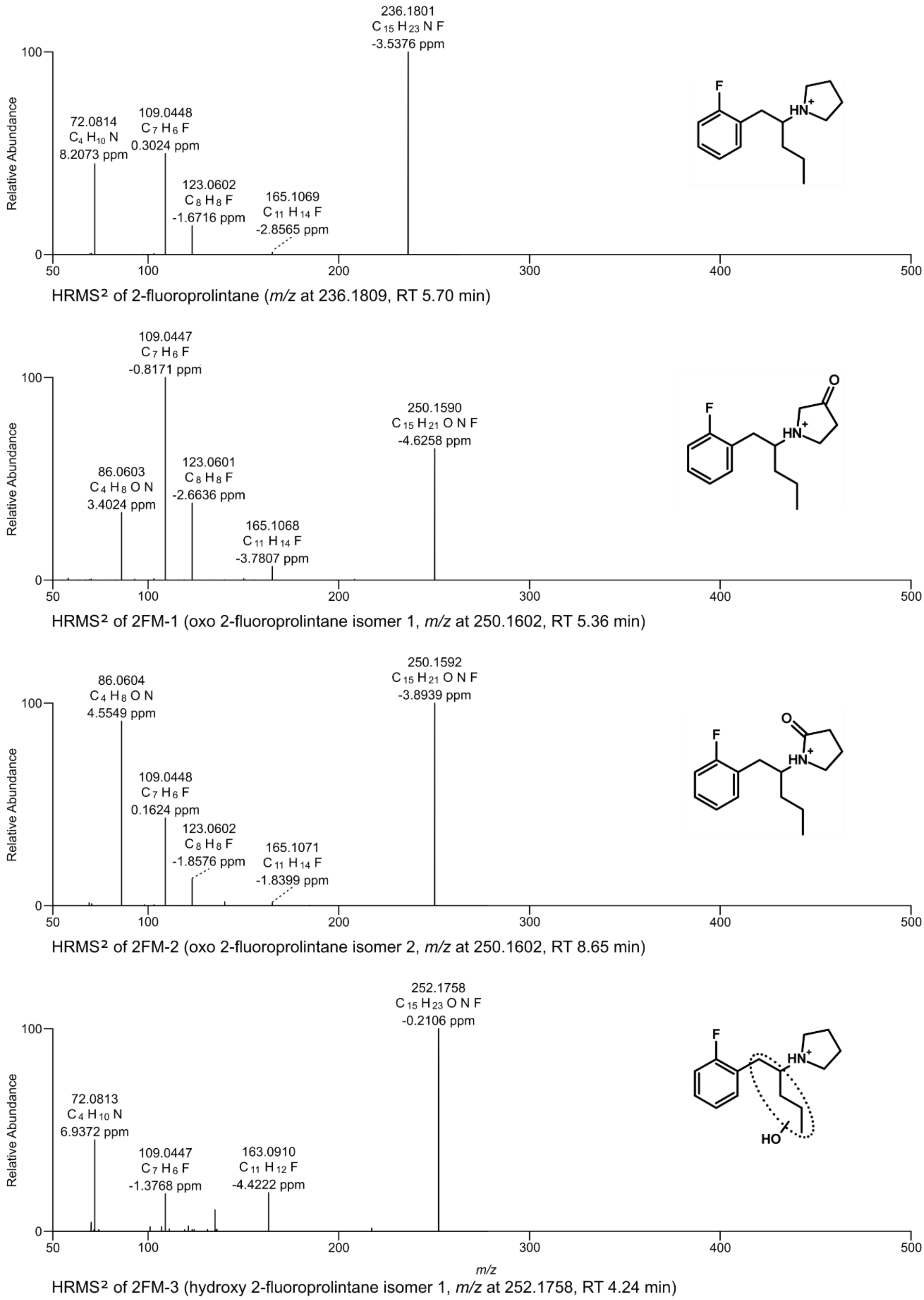

Figure S1 High-resolution (HR) MS^2 spectra and retention times (RT) of 2-fluoroprolintane and its metabolites detected in vivo and/or in vitro.

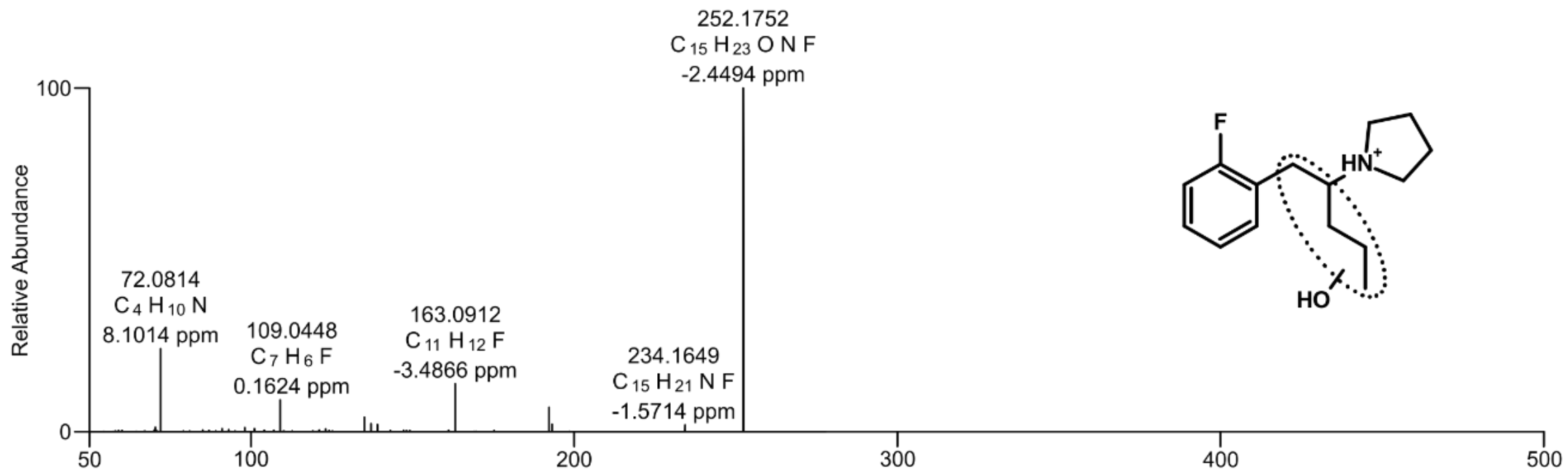

HRMS² of 2FM-4 (hydroxy 2-fluoroprolintane isomer 2, *m/z* at 252.1758, RT 4.60 min)

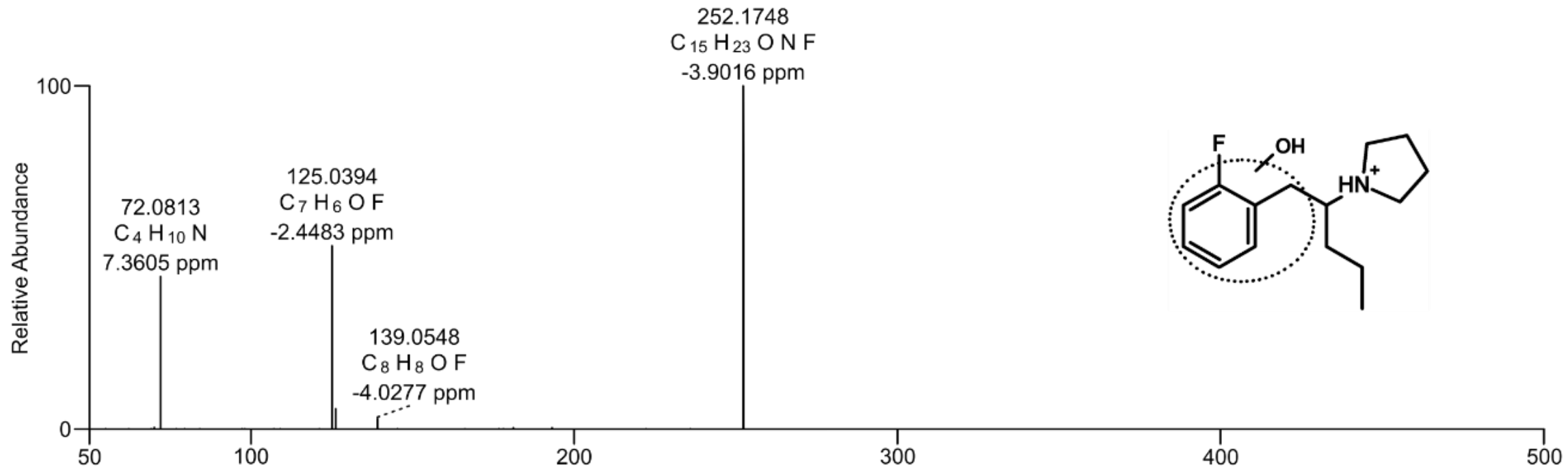

HRMS² of 2FM-5 (hydroxy 2-fluoroprolintane isomer 3, *m/z* at 252.1758, RT 4.72 min)

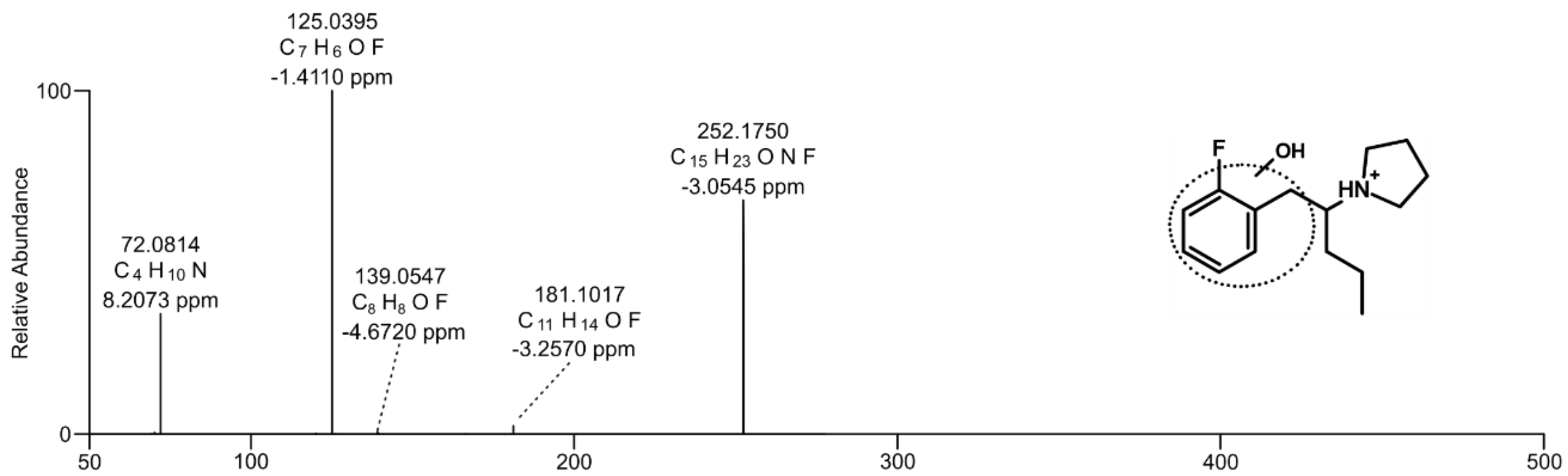

HRMS² of 2FM-6 (hydroxy 2-fluoroprolintane isomer 4, *m/z* at 252.1758, RT 4.95 min)

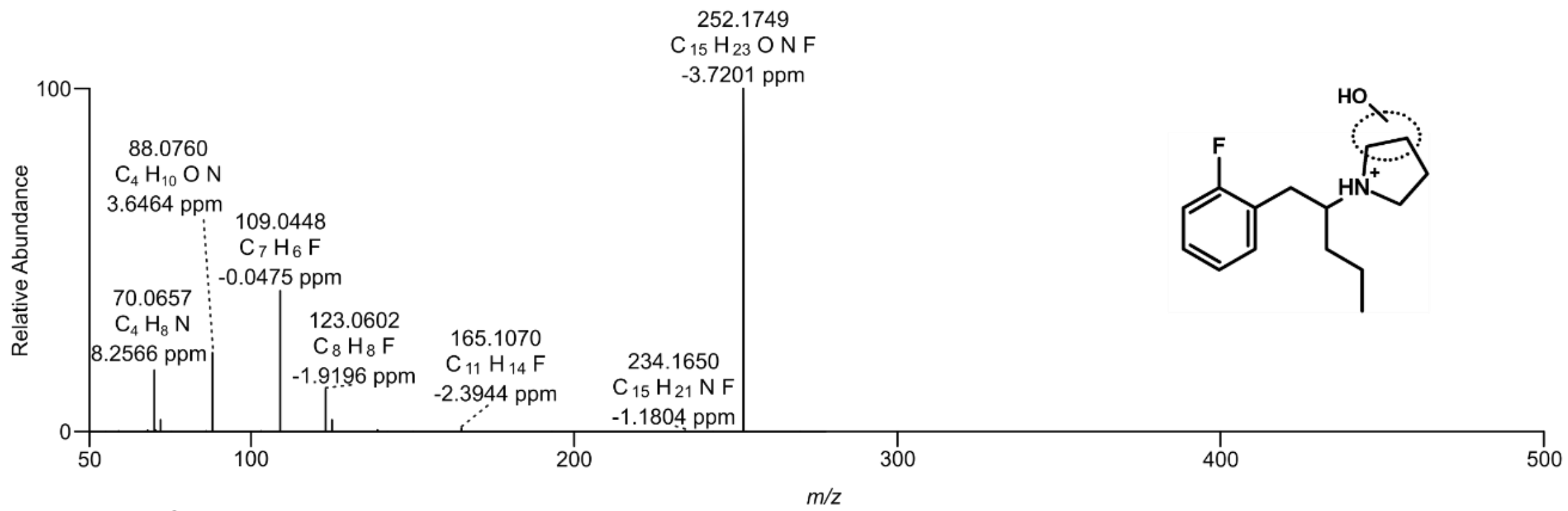

HRMS² of 2FM-7 (hydroxy 2-fluoroprolintane isomer 5, *m/z* at 252.1758, RT 5.36 min)

Figure S1 (continued)

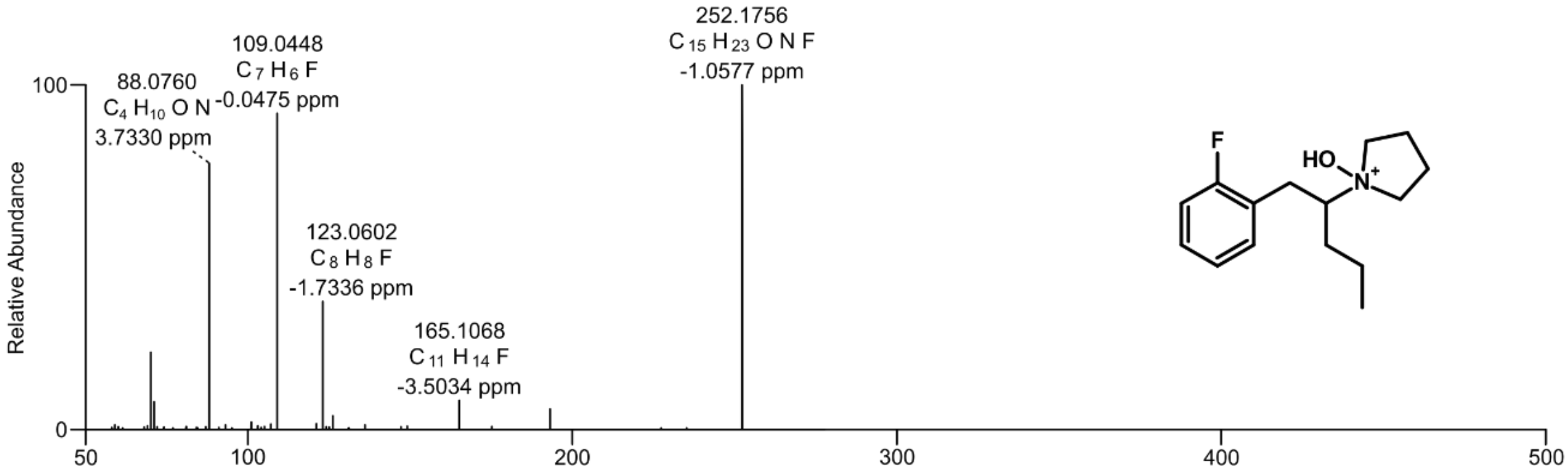

HRMS² of 2FM-8 (2-fluoroprolintane *N*-oxide, *m/z* at 252.1758, RT 6.03 min)

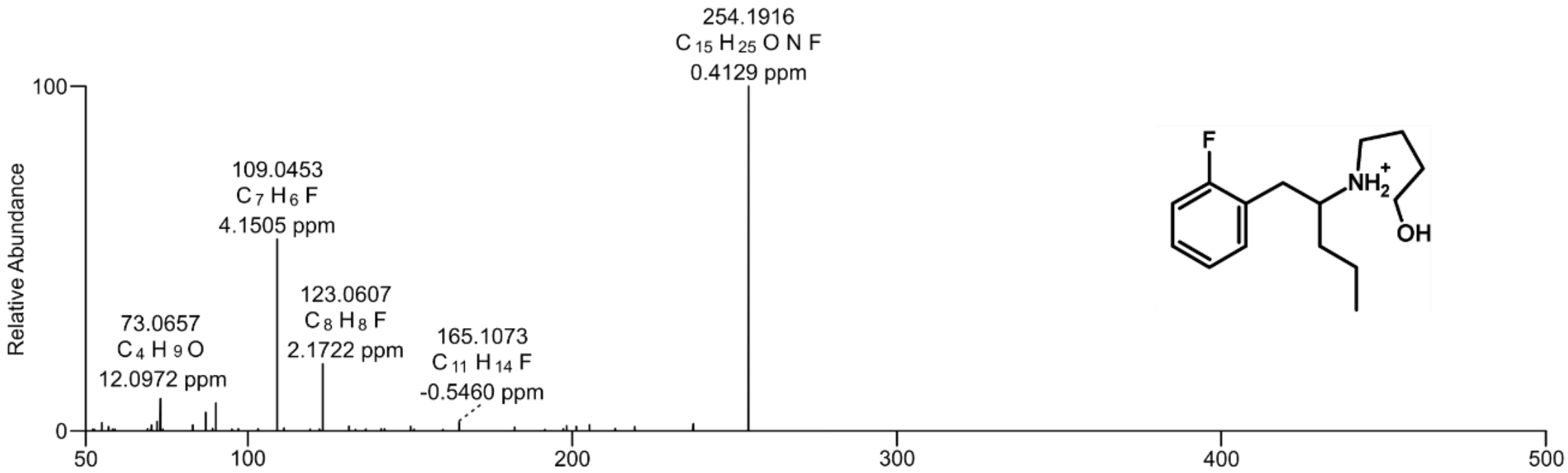

HRMS² of 2FM-9 (ring open hydroxy 2-fluoroprolintane, *m/z* at 254.1915, RT 5.69 min)

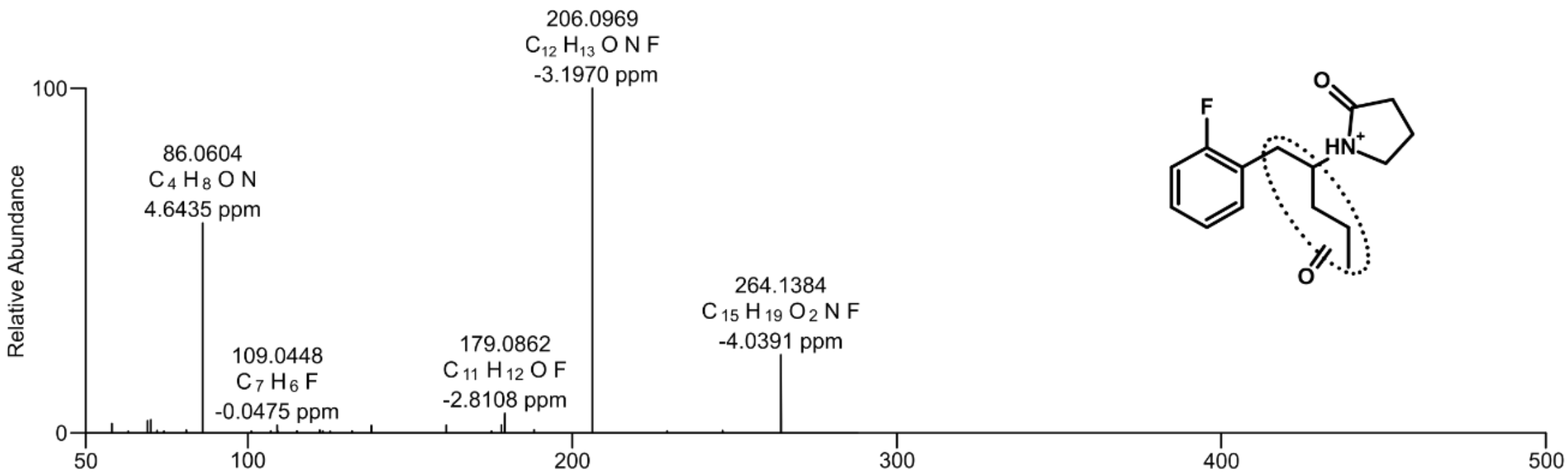

HRMS² of 2FM-10 (dioxo 2-fluoroprolintane, *m/z* at 264.1394, RT 6.78 min)

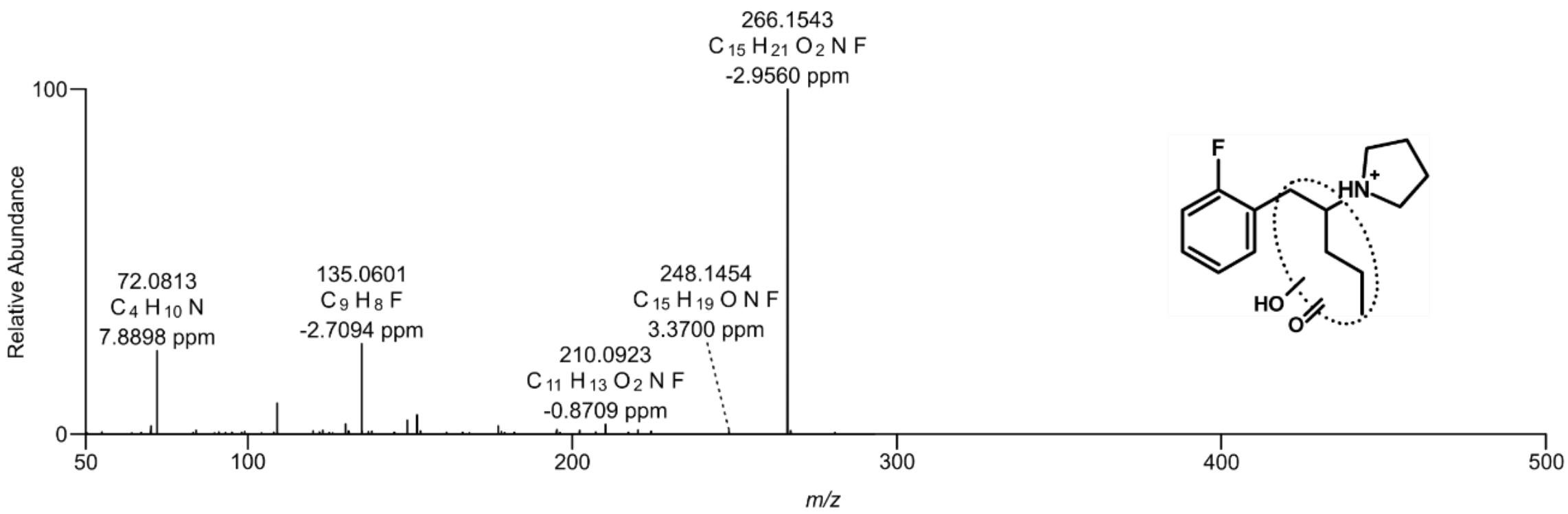

HRMS² of 2FM-11 (oxo hydroxy 2-fluoroprolintane isomer 1, *m/z* at 266.1551, RT 4.34 min)

Figure S1 (continued)

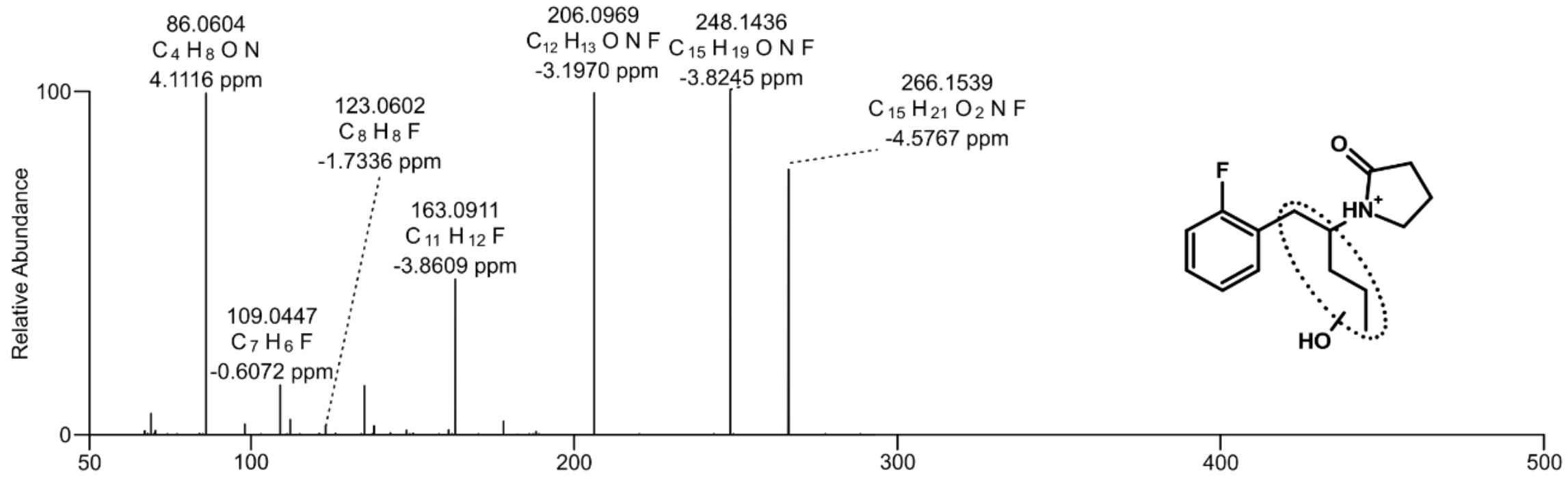

HRMS² of 2FM-12 (oxo hydroxy 2-fluoroprolintane isomer 2, *m/z* at 266.1551, RT 6.59 min)

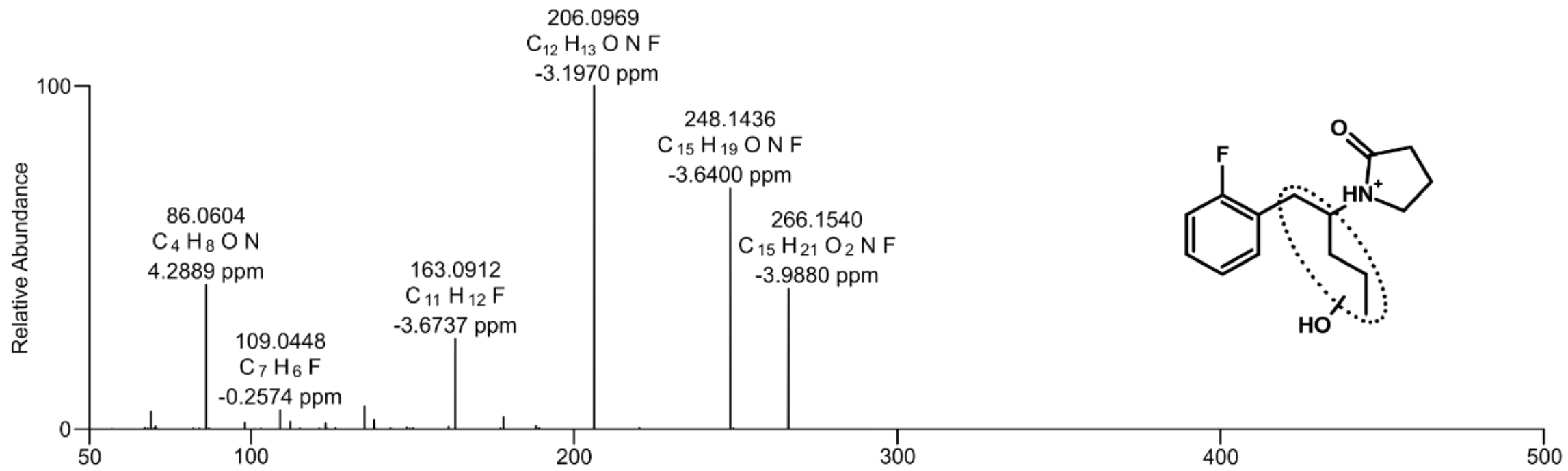

HRMS² of 2FM-13 (oxo hydroxy 2-fluoroprolintane isomer 3, *m/z* at 266.1551, RT 6.95 min)

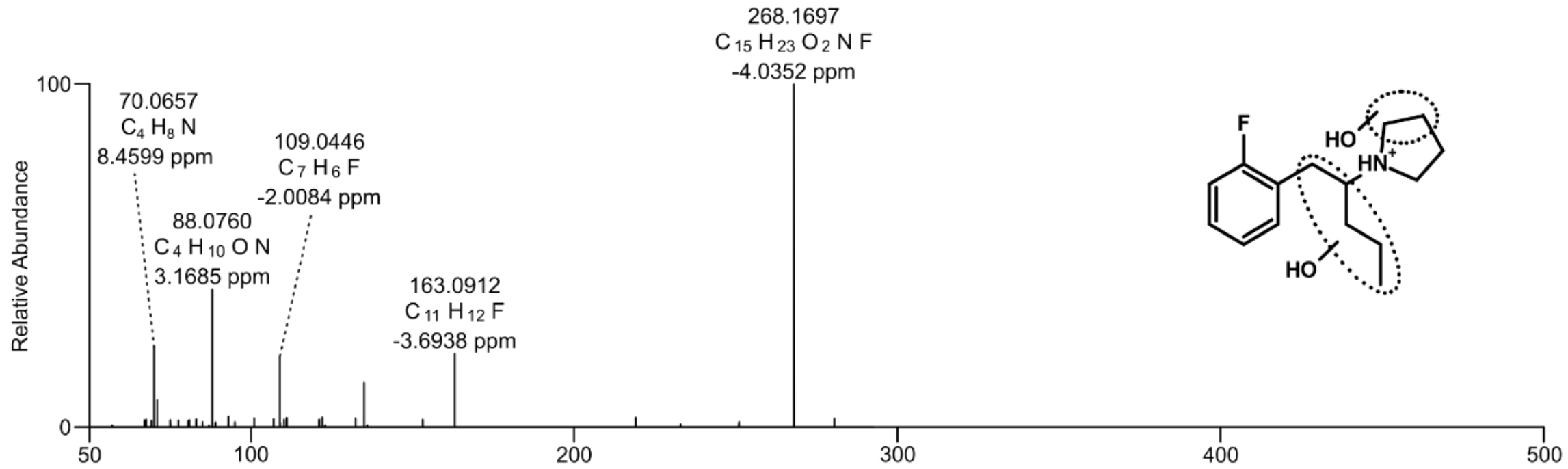

HRMS² of 2FM-14 (dihydroxy 2-fluoroprolintane isomer 1, *m/z* at 268.1707, RT 3.85 min)

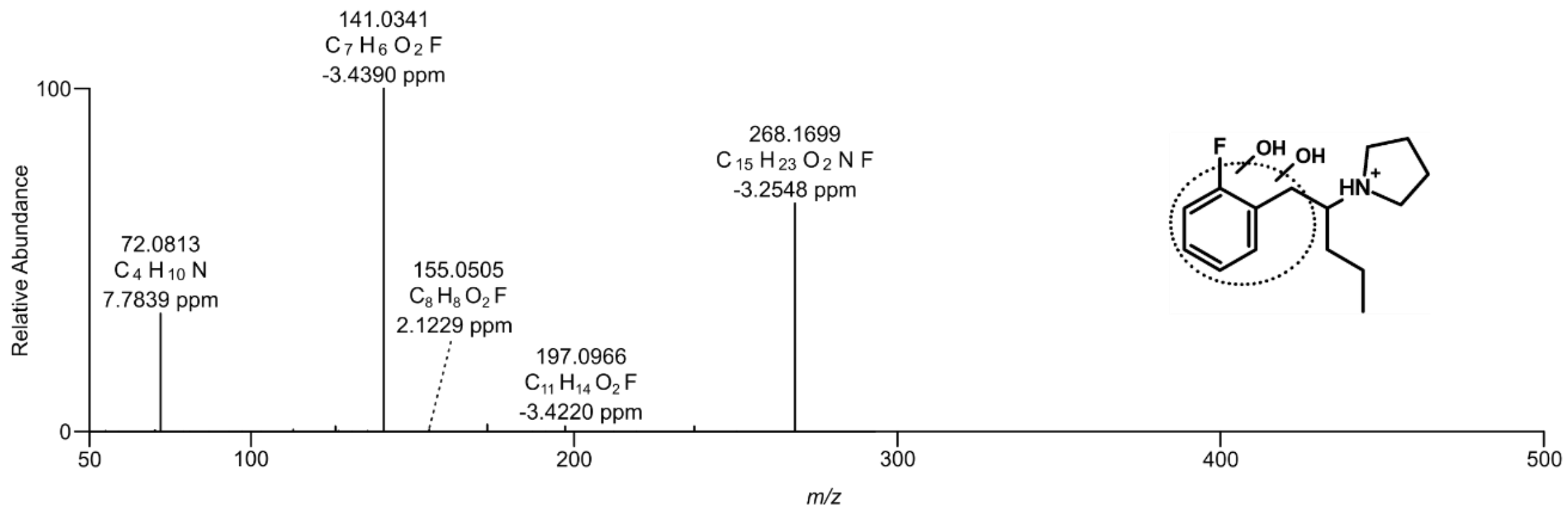

HRMS² of 2FM-15 (dihydroxy 2-fluoroprolintane isomer 2, *m/z* at 268.1707, RT 4.12 min)

Figure S1 (continued)

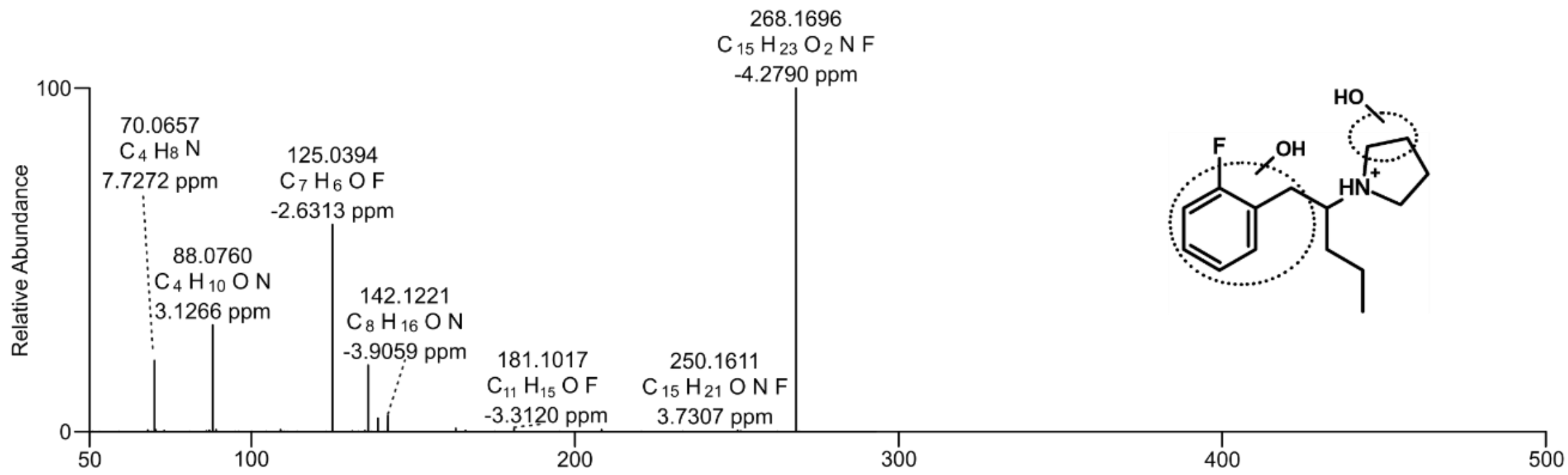

HRMS² of 2FM-16 (dihydroxy 2-fluoroprolintane isomer 3, *m/z* at 268.1707, RT 4.35 min)

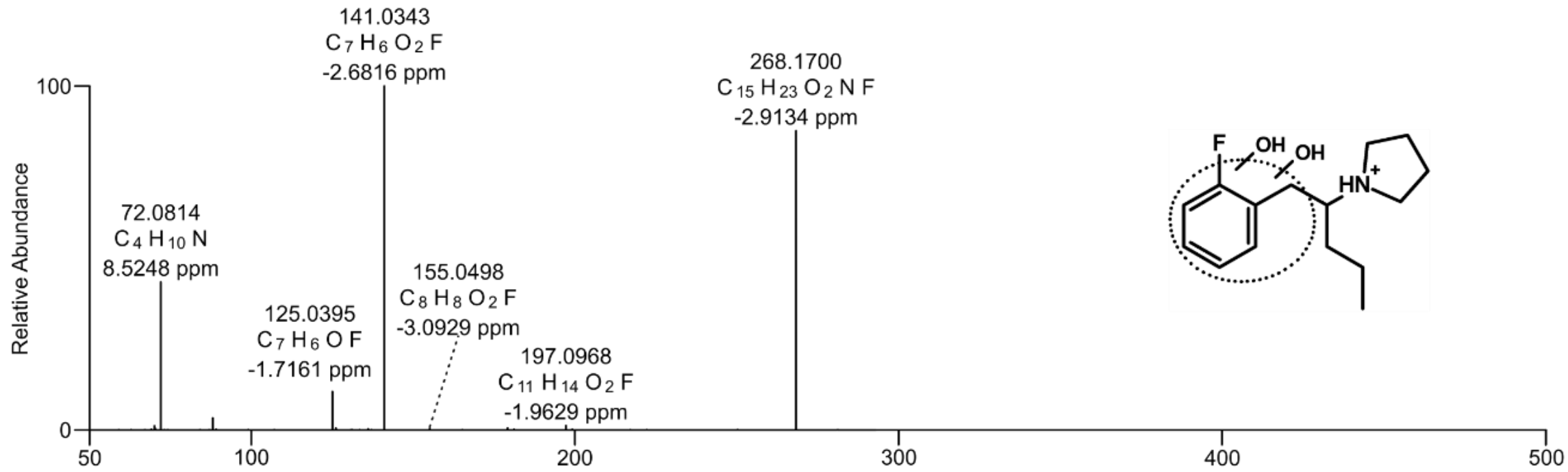

HRMS² of 2FM-17 (dihydroxy 2-fluoroprolintane isomer 4, *m/z* at 268.1707, RT 4.52 min)

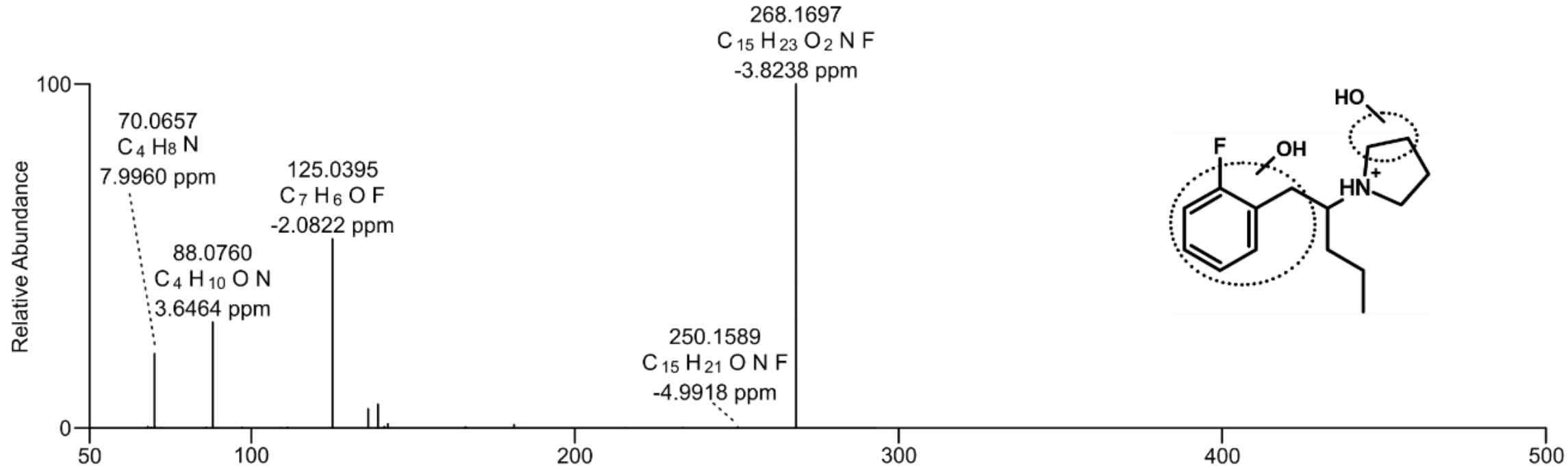

HRMS² of 2FM-18 (dihydroxy 2-fluoroprolintane isomer 5, *m/z* at 268.1707, RT 4.63 min)

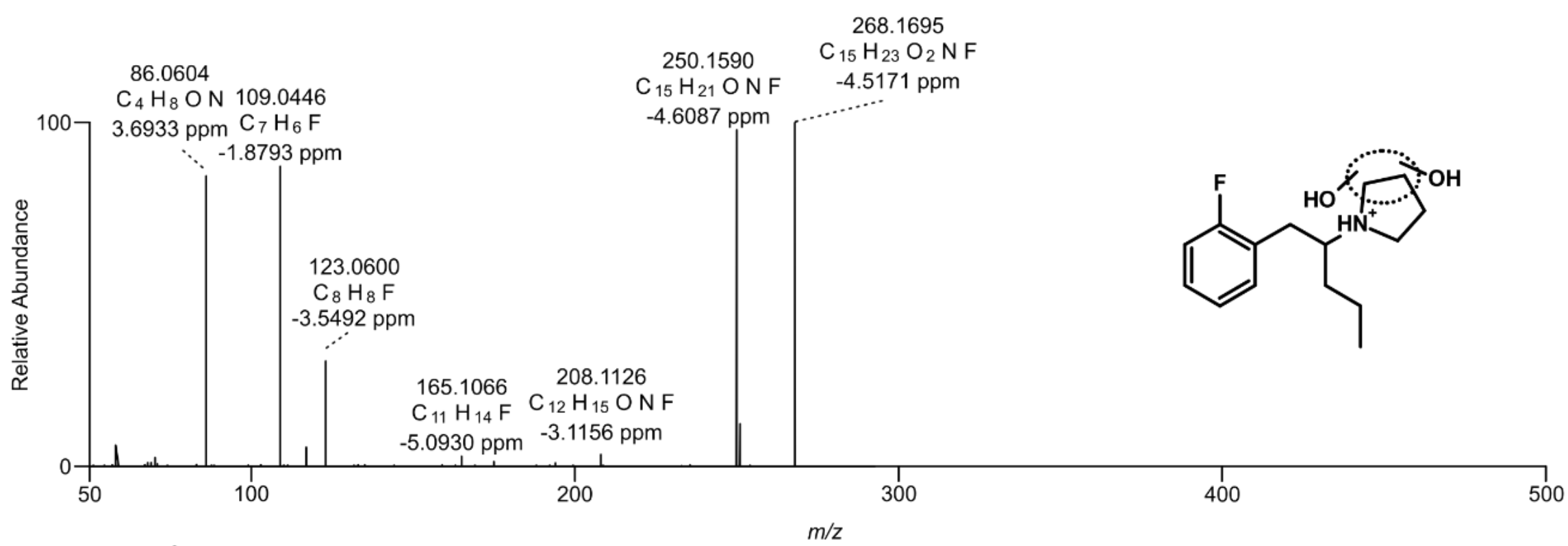

HRMS² of 2FM-19 (dihydroxy 2-fluoroprolintane isomer 6, *m/z* at 268.1707, RT 5.30 min)

Figure S1 (continued)

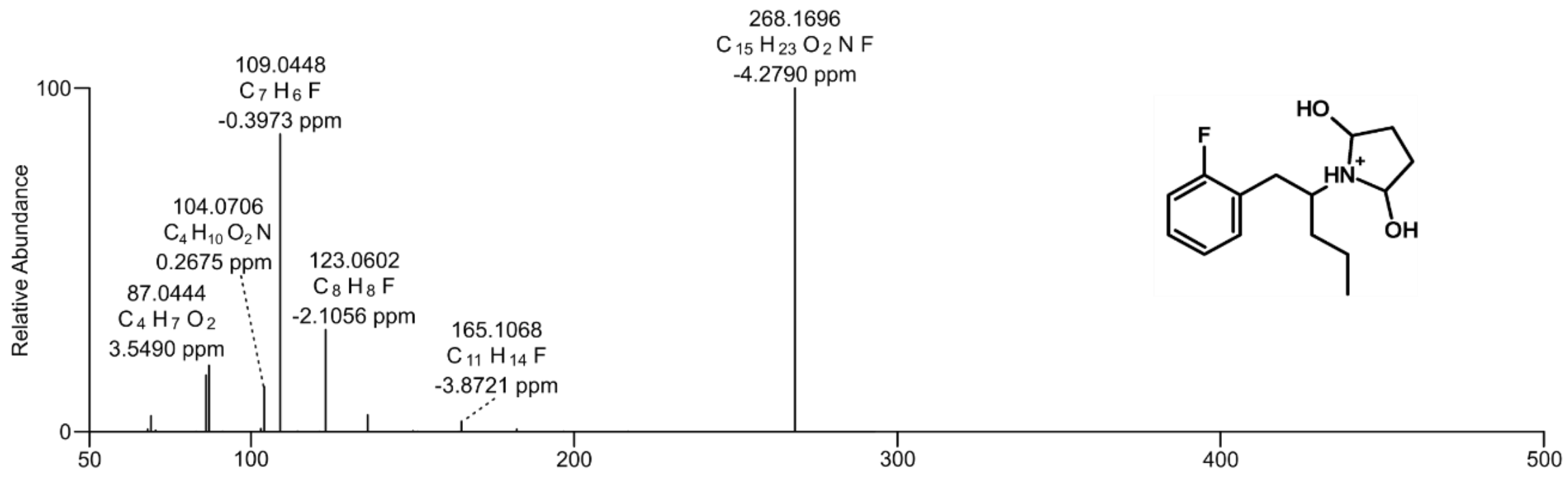

HRMS^2 of 2FM-20 (dihydroxy 2-fluoroprolintane isomer 7, *m/z* at 268.1707, RT 5.64 min)

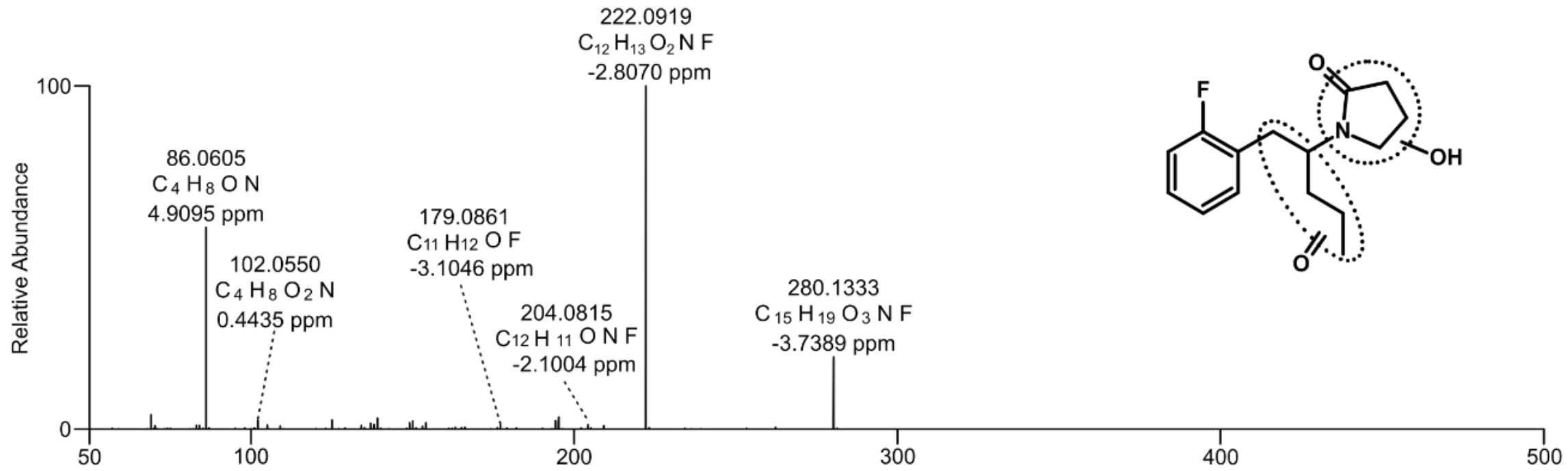

HRMS^2 of 2FM-21 (dioxo hydroxy 2-fluoroprolintane, *m/z* at 280.1343, RT 5.90 min)

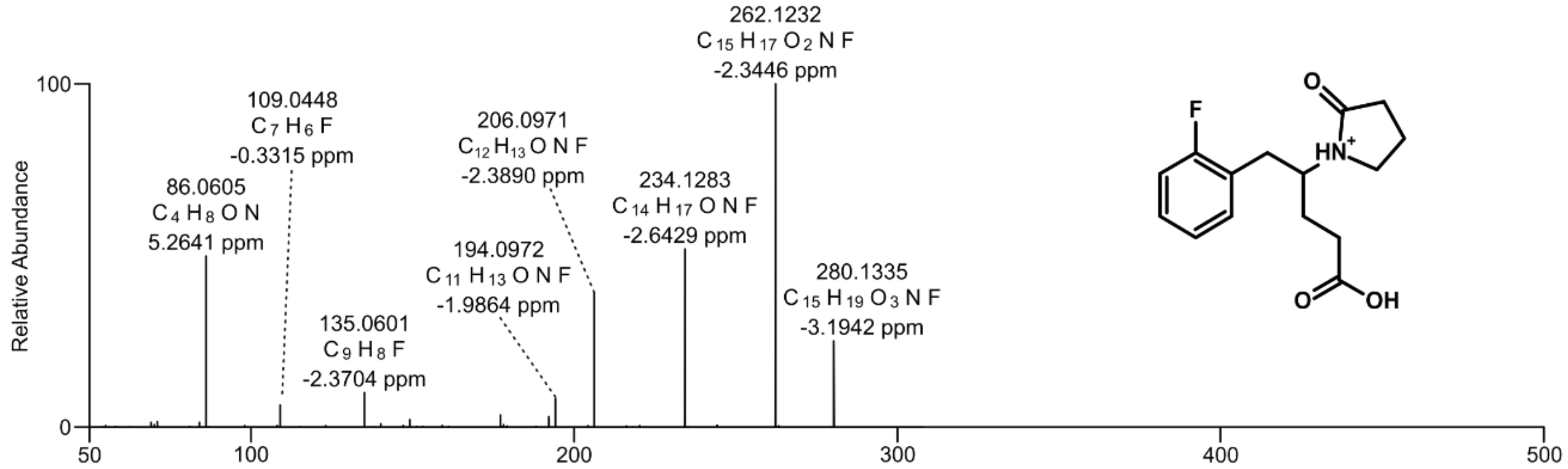

HRMS^2 of 2FM-22 (oxo 2-fluoroprolintane carboxylic acid, *m/z* at 280.1343, RT 6.78 min)

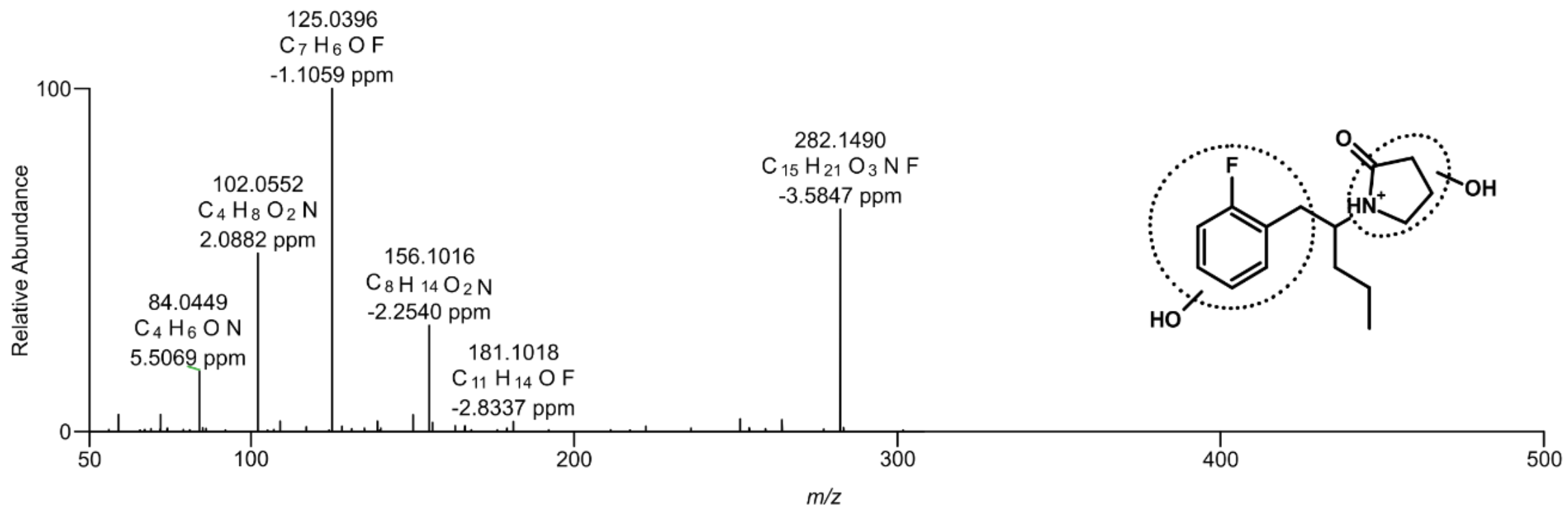

HRMS^2 of 2FM-23 (oxo dihydroxy 2-fluoroprolintane isomer 1, *m/z* at 282.1500, RT 5.34 min)

Figure S1 (continued)

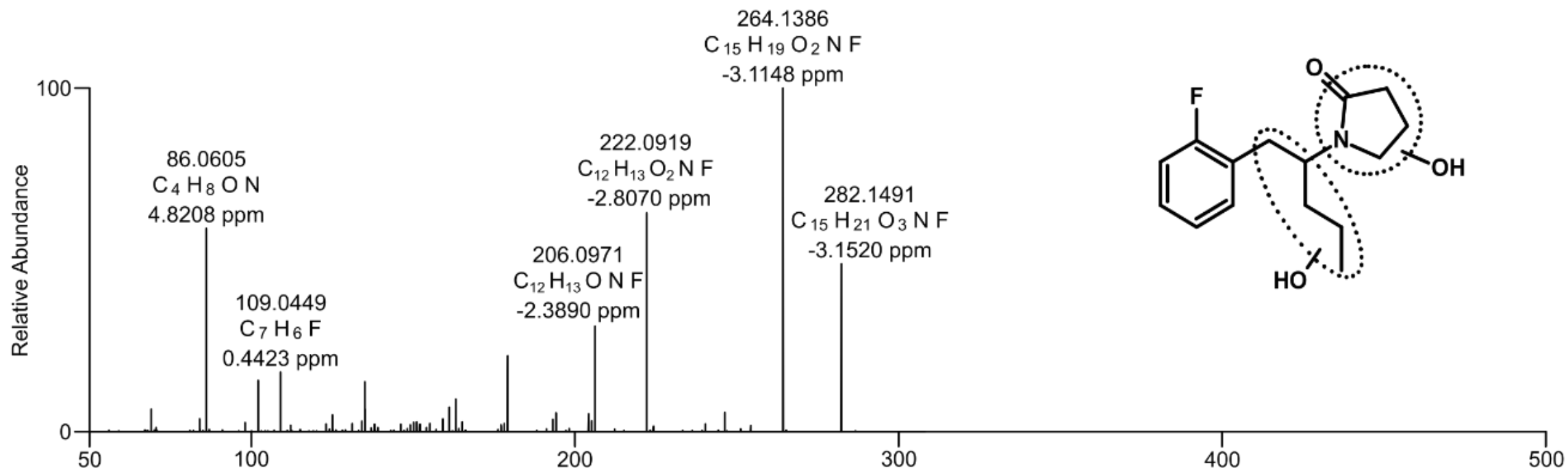

HRMS² of 2FM-24 (oxo dihydroxy 2-fluoroprolintane isomer 2, *m/z* at 282.1500, RT 5.91 min)

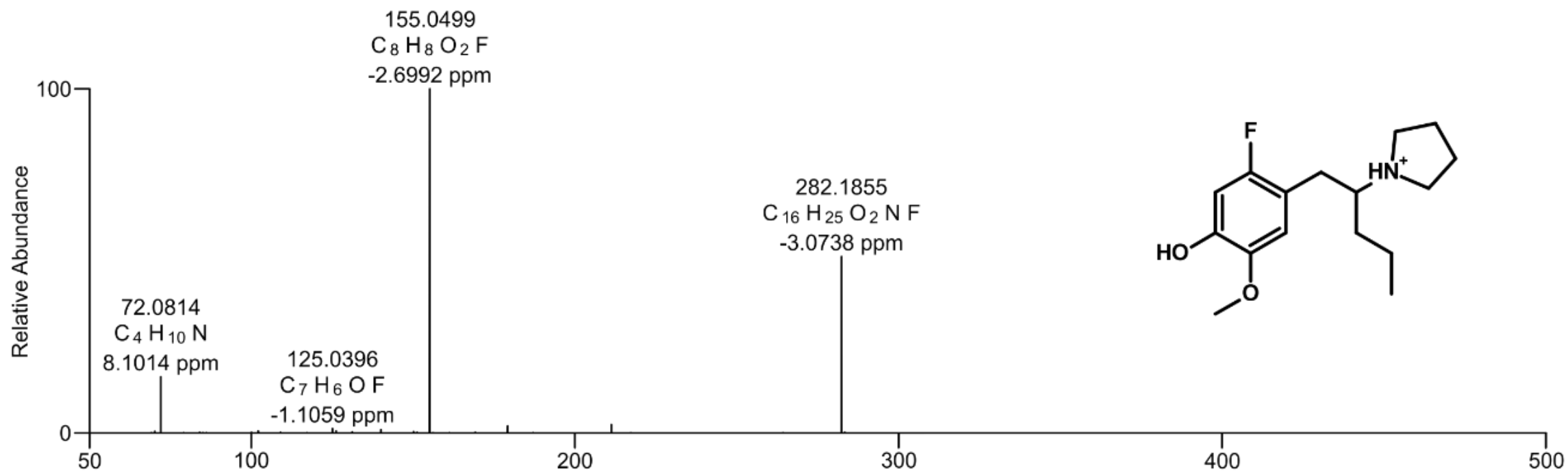

HRMS² of 2FM-25 (hydroxy methoxy 2-fluoroprolintane, *m/z* at 282.1864, RT 5.08 min)

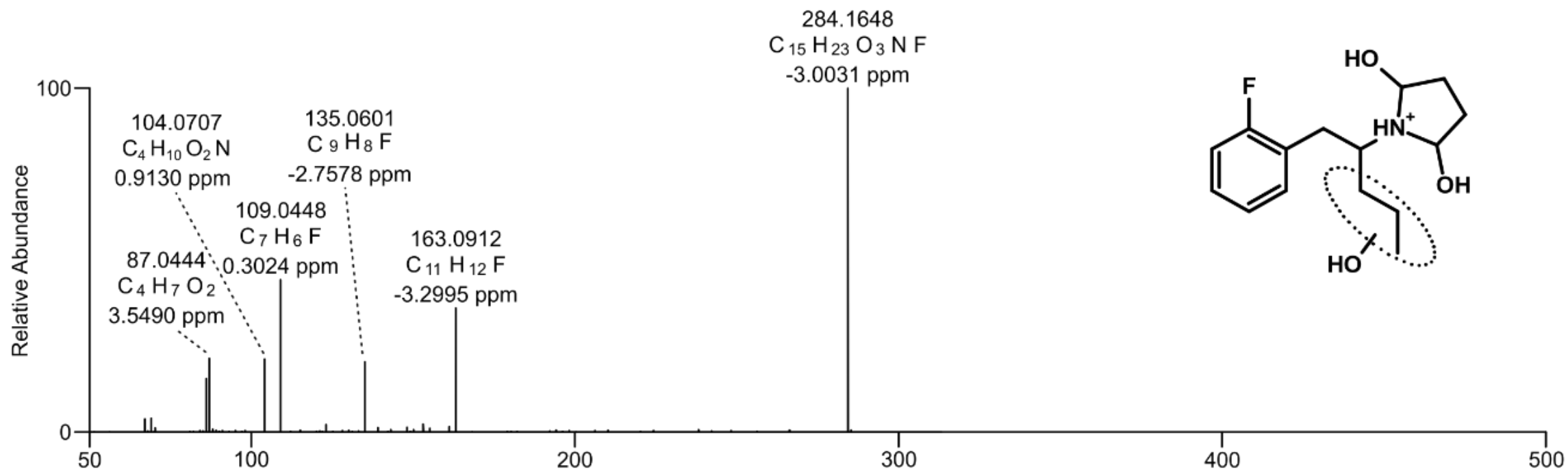

HRMS² of 2FM-26 (trihydroxy 2-fluoroprolintane isomer 1, *m/z* at 284.1656, RT 4.28 min)

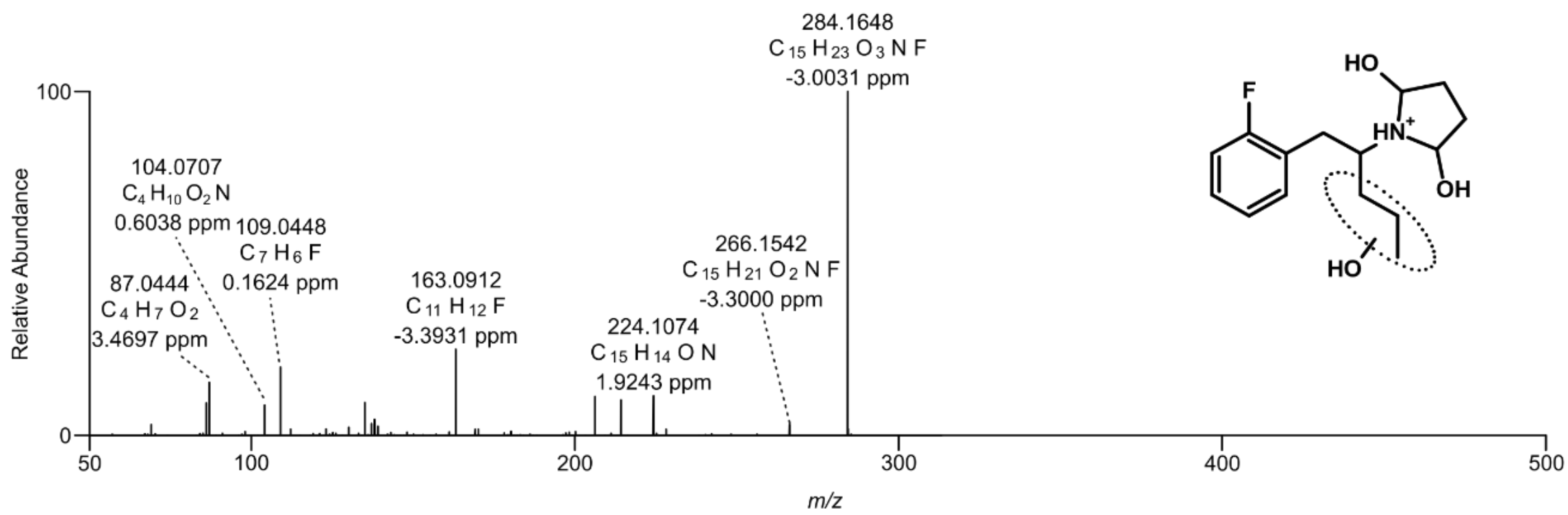

HRMS² of 2FM-27 (trihydroxy 2-fluoroprolintane isomer 2, *m/z* at 284.1656, RT 4.69 min)

Figure S1 (continued)

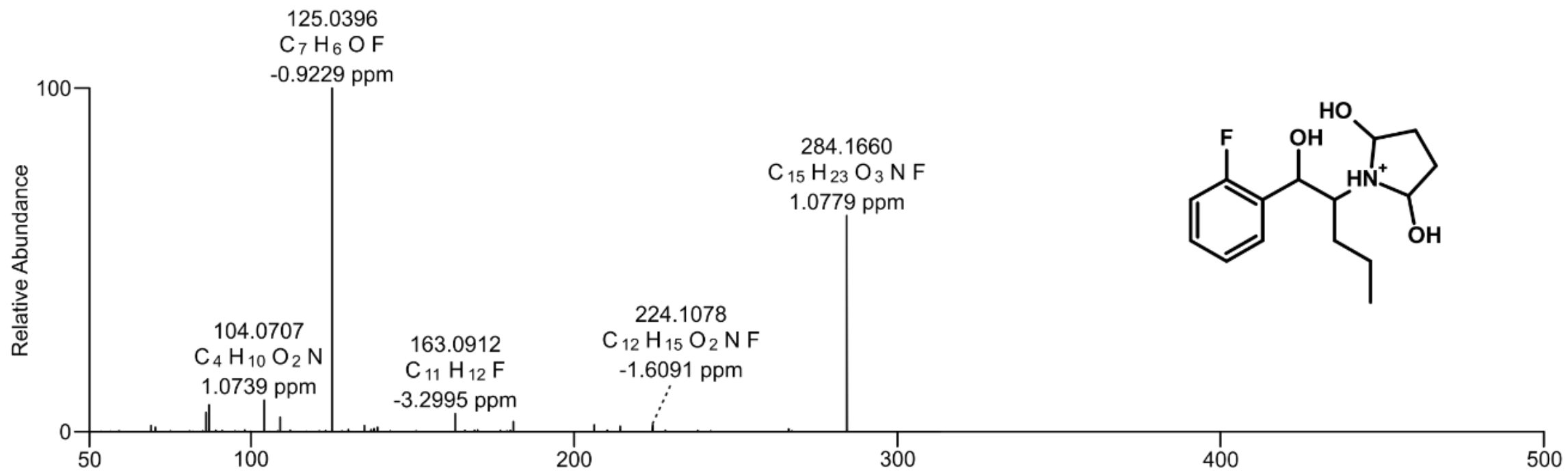

HRMS² of 2FM-28 (trihydroxy 2-fluoroprolintane isomer 3, *m/z* at 284.1656, RT 4.88 min)

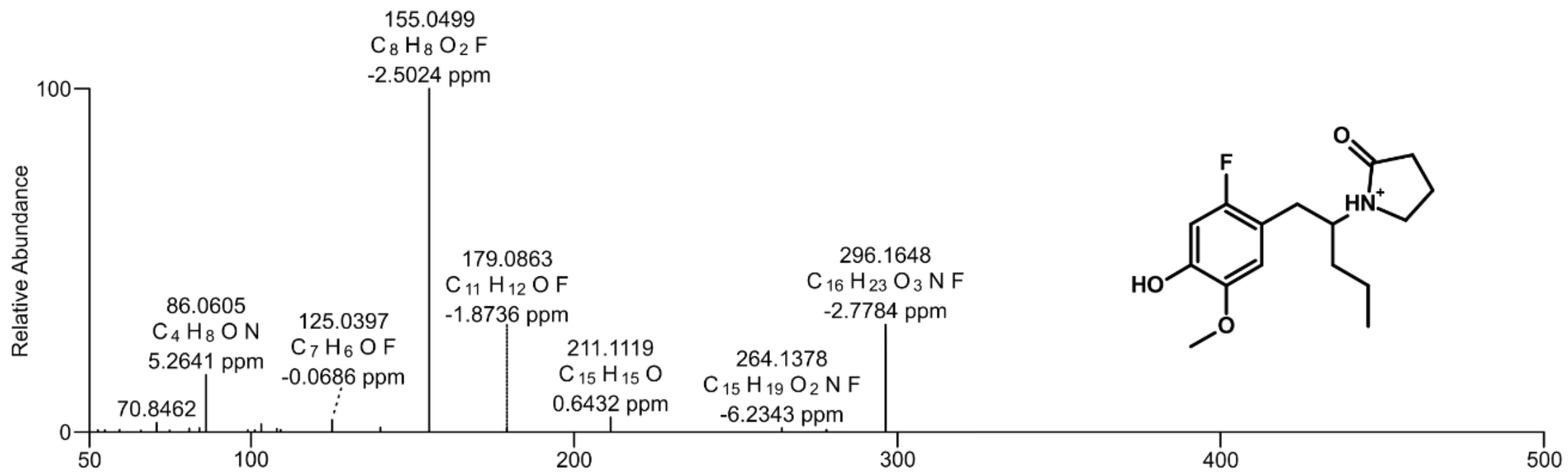

HRMS² of 2FM-29 (oxo hydroxy methoxy 2-fluoroprolintane, *m/z* at 296.1656, RT 7.97 min)

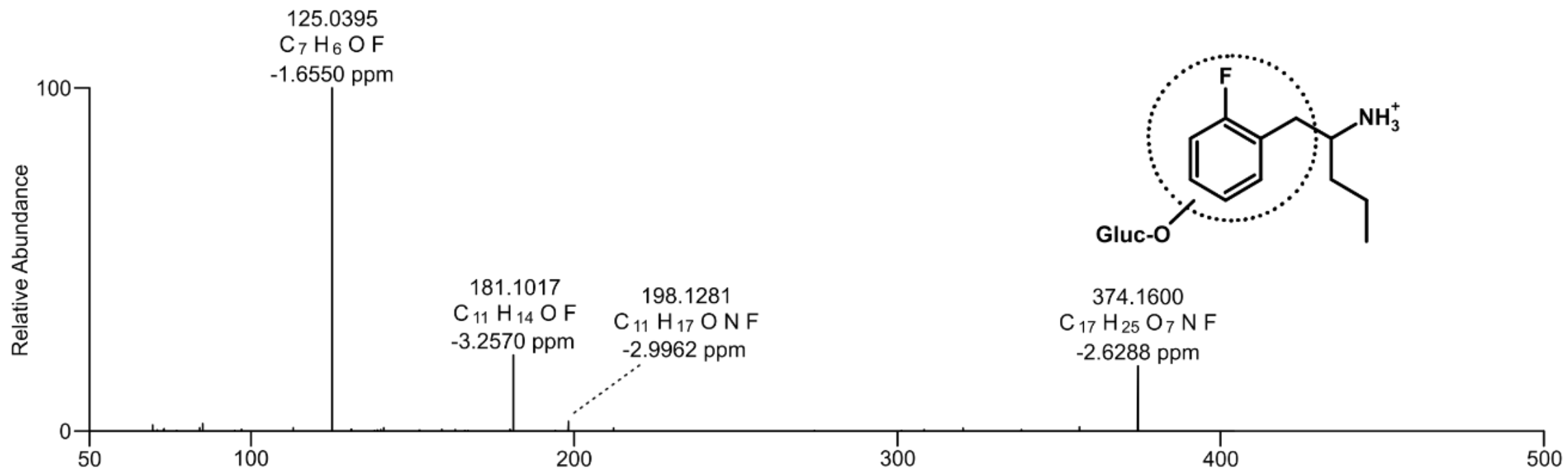

HRMS² of 2FM-30 (*N,N-bis*-dealkyl hydroxy 2-fluoroprolintane glucuronide, *m/z* at 374.1610, RT 3.42 min)

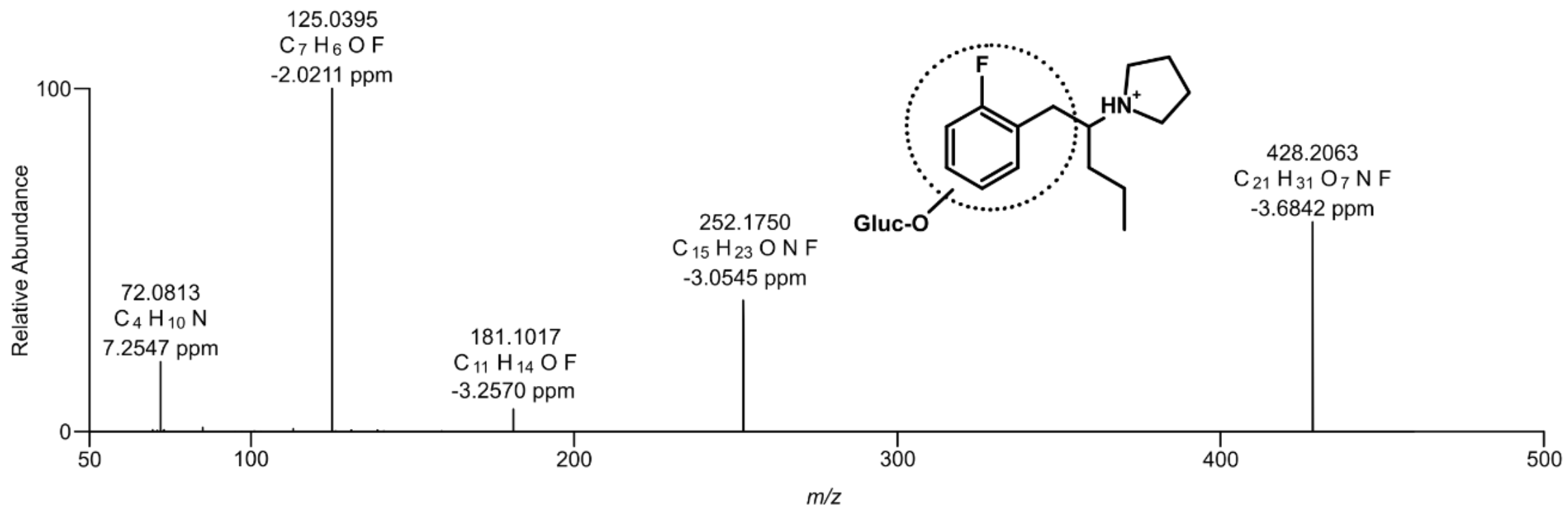

HRMS² of 2FM-31 (hydroxy 2-fluoroprolintane glucuronide, *m/z* at 428.2079, RT 3.77 min)

Figure S1 (continued)

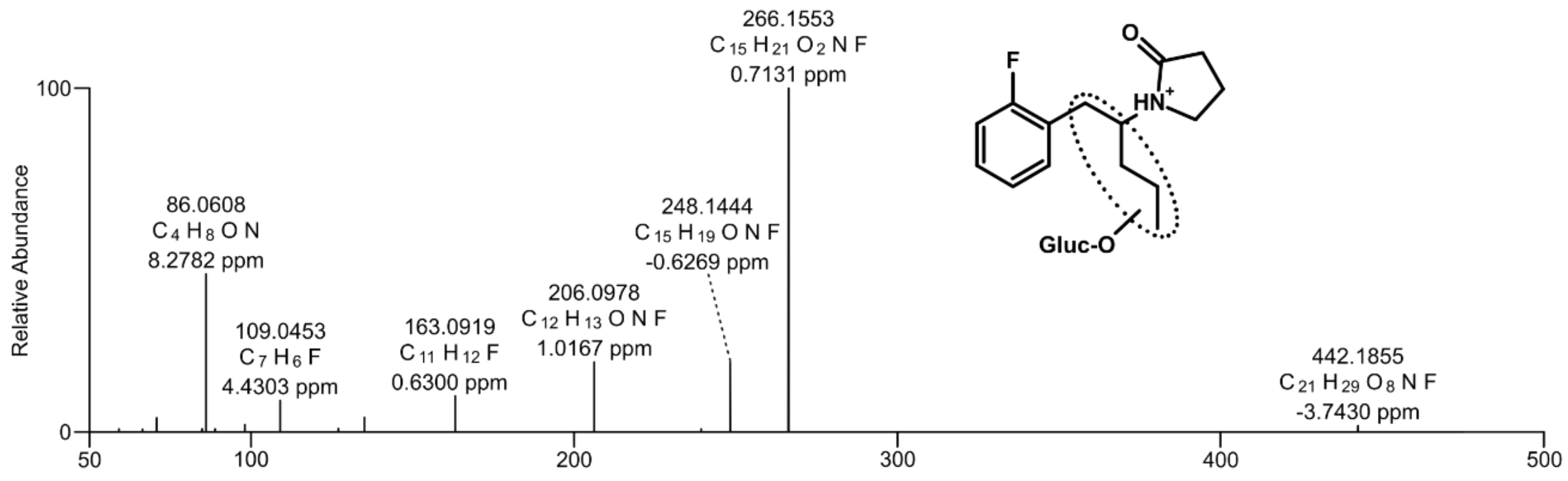

HRMS² of 2FM-32 (oxo hydroxy 2-fluoroprolintane glucuronide, *m/z* at 442.1872, RT 6.02 min)

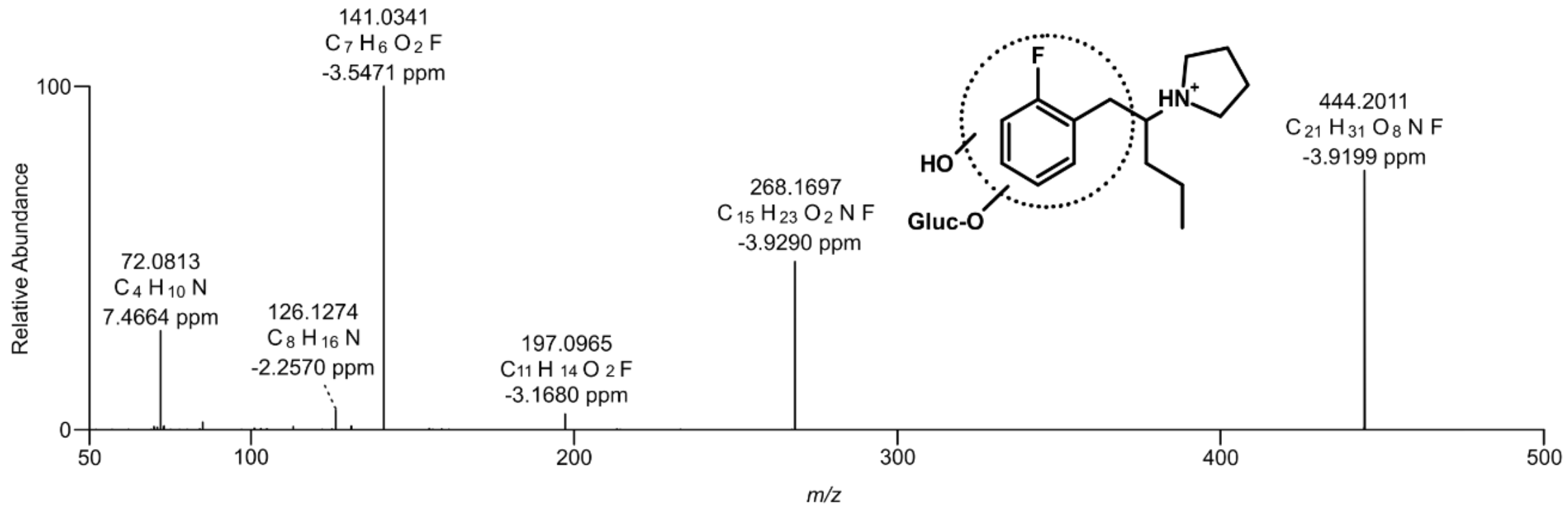

HRMS² of 2FM-33 (dihydroxy 2-fluoroprolintane glucuronide, *m/z* at 444.2028, RT 3.64 min)

Figure S1 (continued)

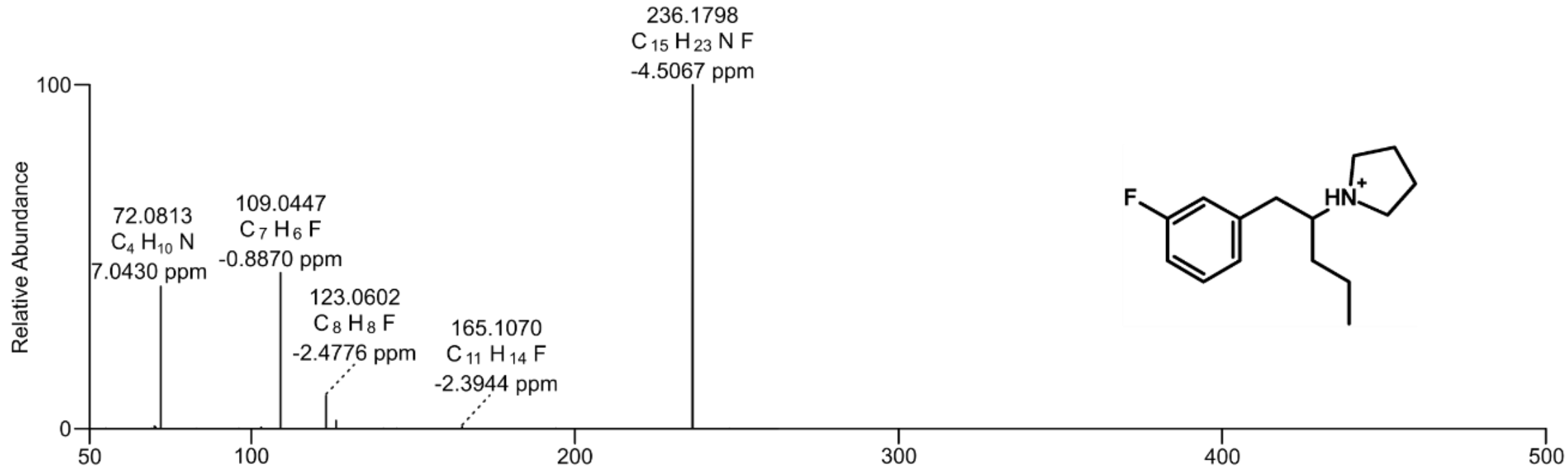

HRMS² of 3-fluoroprolintane (*m/z* at 236.1809, RT 5.74 min)

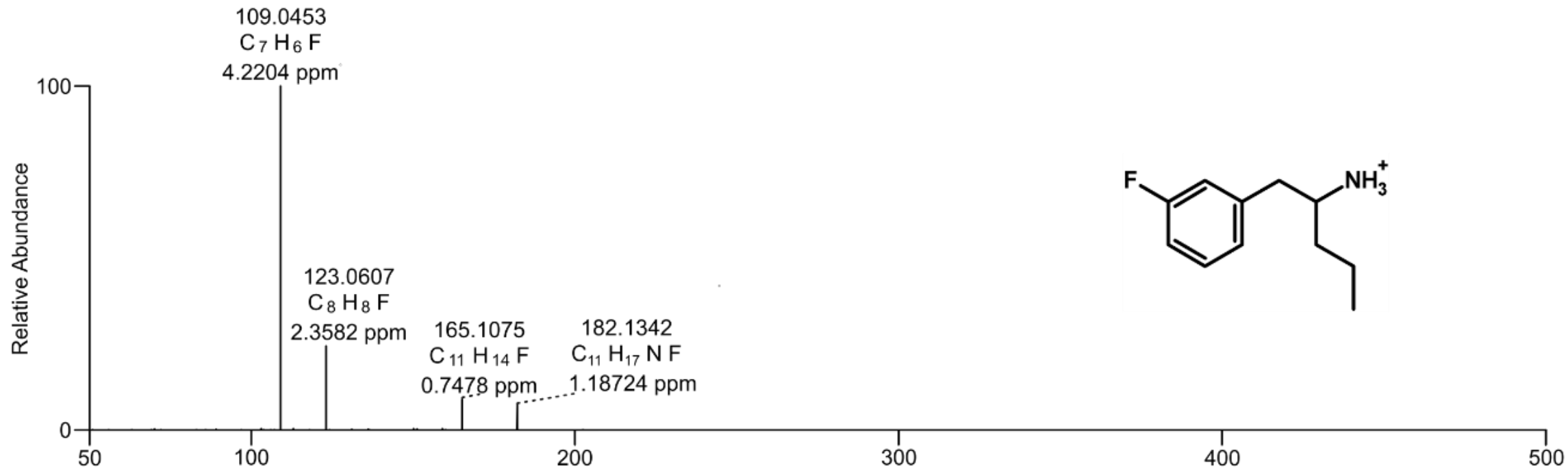

HRMS² of 3FM-1 (*N,N-bis*-dealkyl 3-fluoroprolintane, *m/z* at 182.1340, RT 5.14 min)

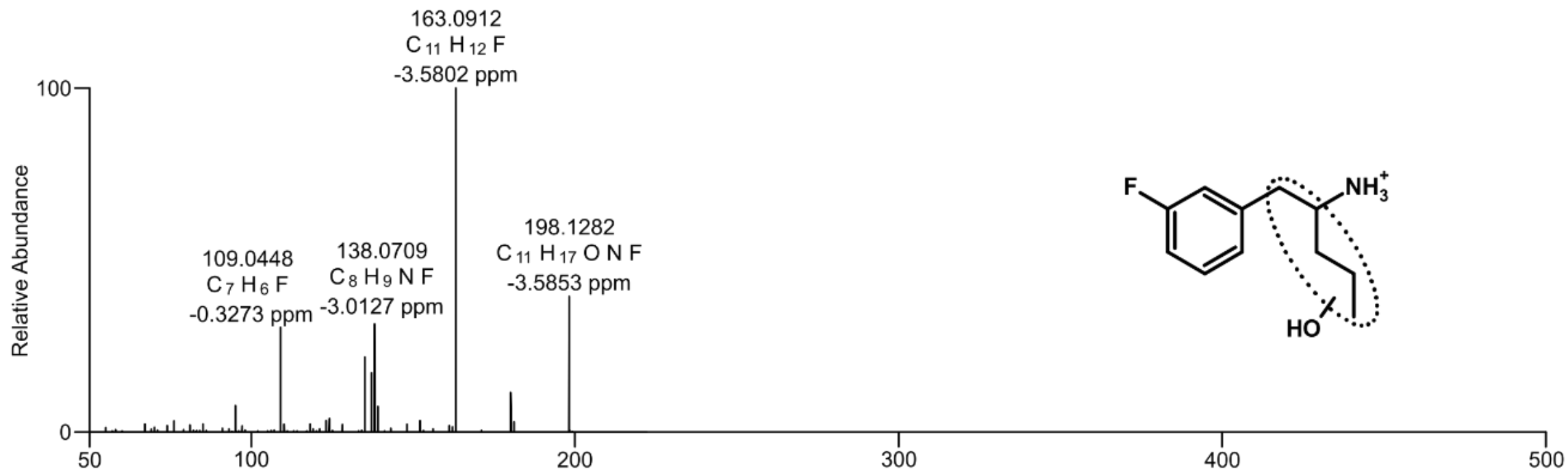

HRMS² of 3FM-2 (*N,N-bis*-dealkyl hydroxy 3-fluoroprolintane, *m/z* at 198.1289, RT 3.96 min)

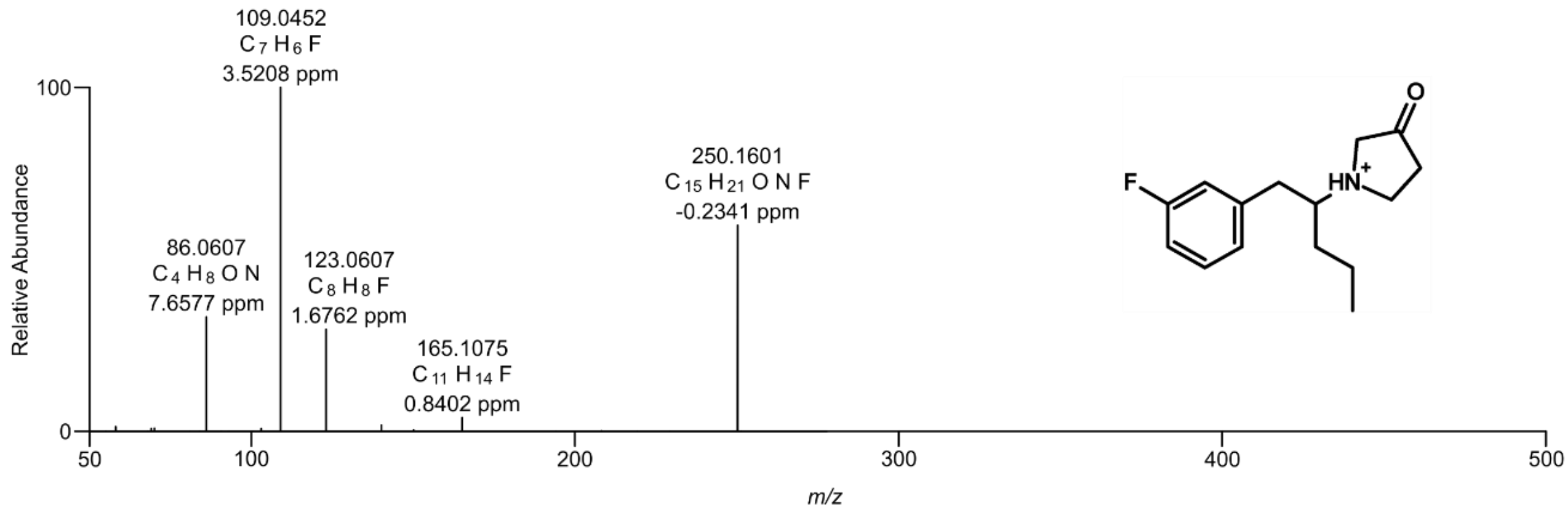

HRMS² of 3FM-3 (oxo 3-fluoroprolintane isomer 1, *m/z* at 250.1602, RT 5.40 min)

Figure S2 High-resolution (HR) MS^2 spectra and retention times (RT) of 3-fluoroprolintane and its metabolites detected in vivo and/or in vitro.

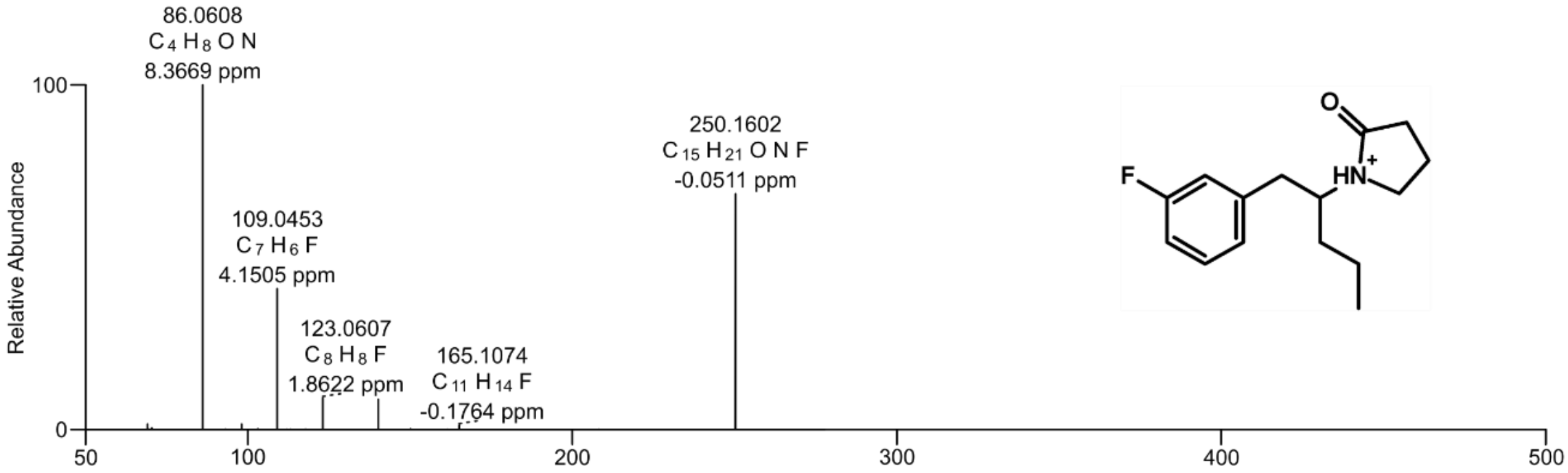

HRMS² of 3FM-4 (oxo 3-fluoroprolintane isomer 2, *m/z* at 250.1602, RT 8.59 min)

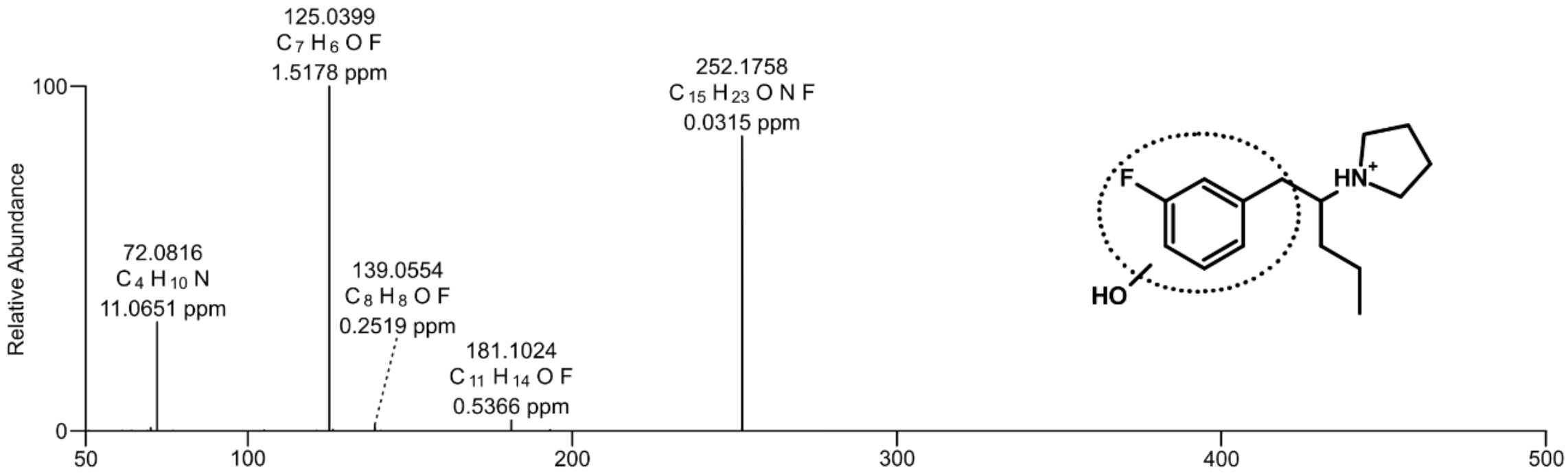

HRMS² of 3FM-5 (hydroxy 3-fluoroprolintane isomer 1, *m/z* at 252.1758, RT 4.53 min)

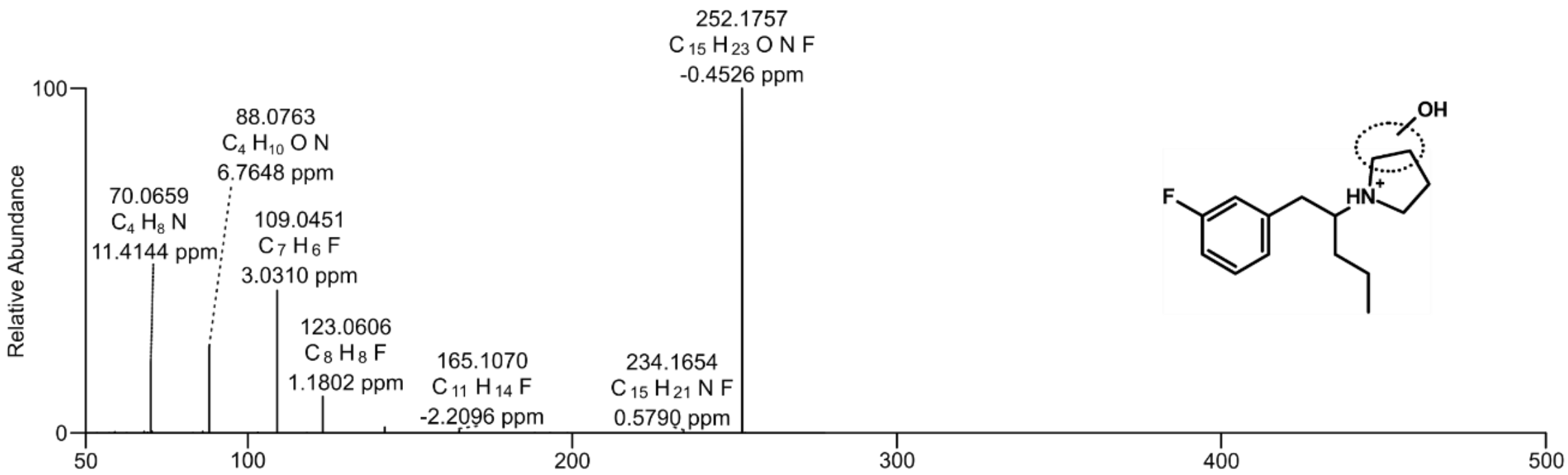

HRMS² of 3FM-6 (hydroxy 3-fluoroprolintane isomer 2, *m/z* at 252.1758, RT 5.42 min)

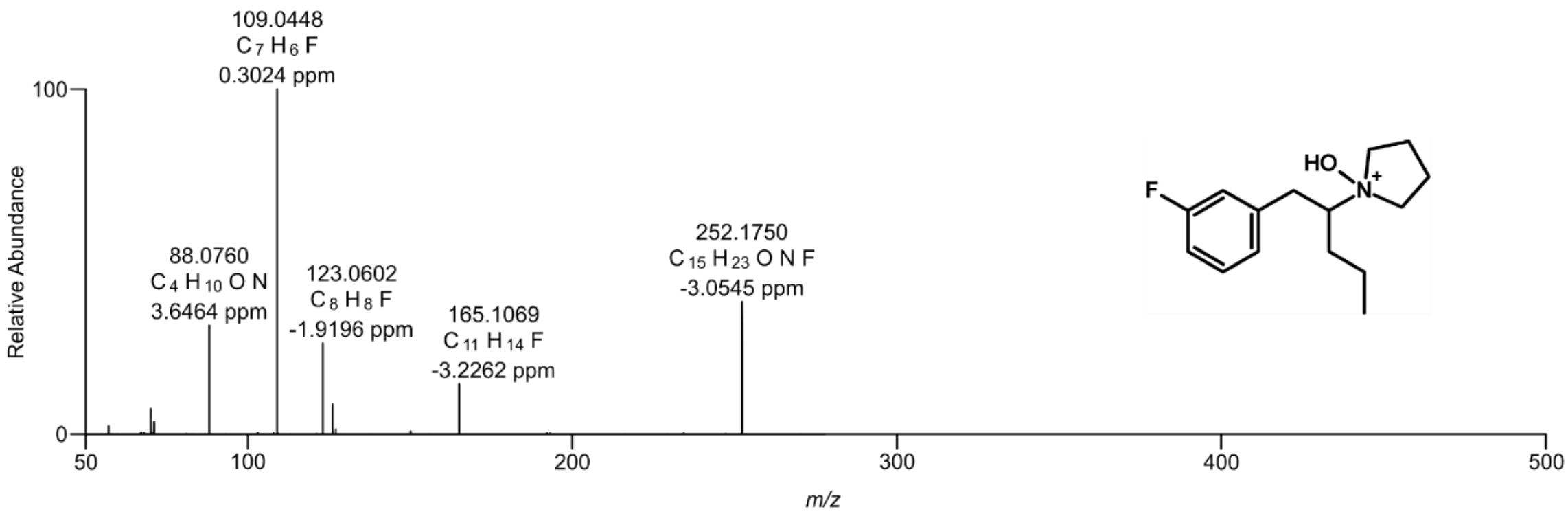

HRMS² of 3FM-7 (3-fluoroprolintane *N*-oxide, *m/z* at 252.1758, RT 6.24 min)

Figure S2 (continued)

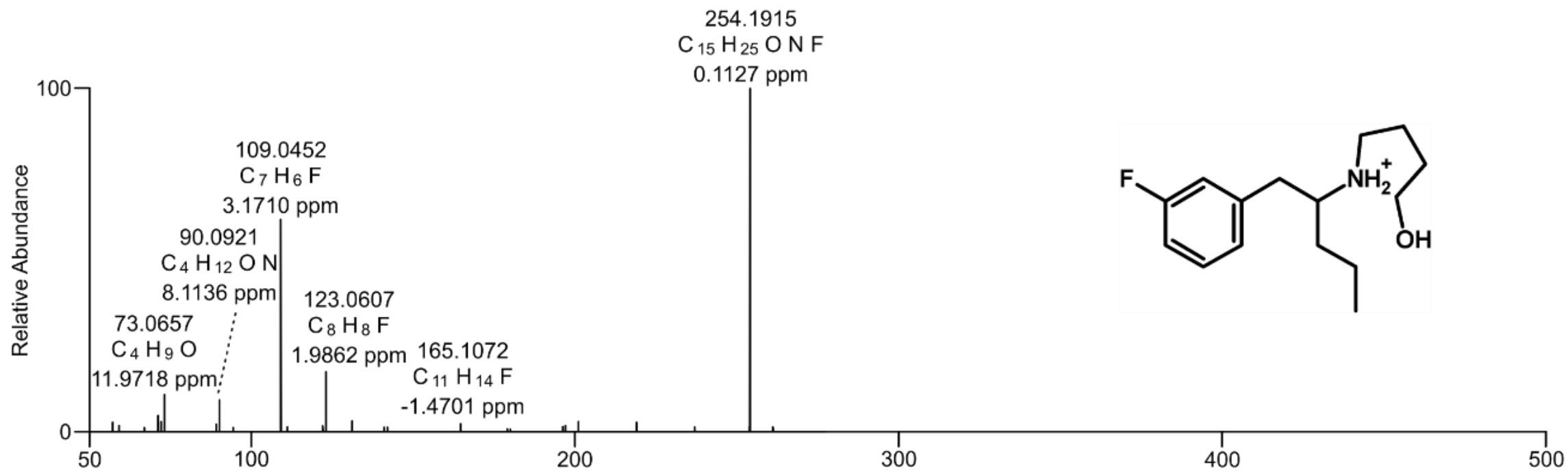

HRMS² of 3FM-8 (ring open hydroxy 3-fluoroprolintane, *m/z* at 254.1915, RT 5.72 min)

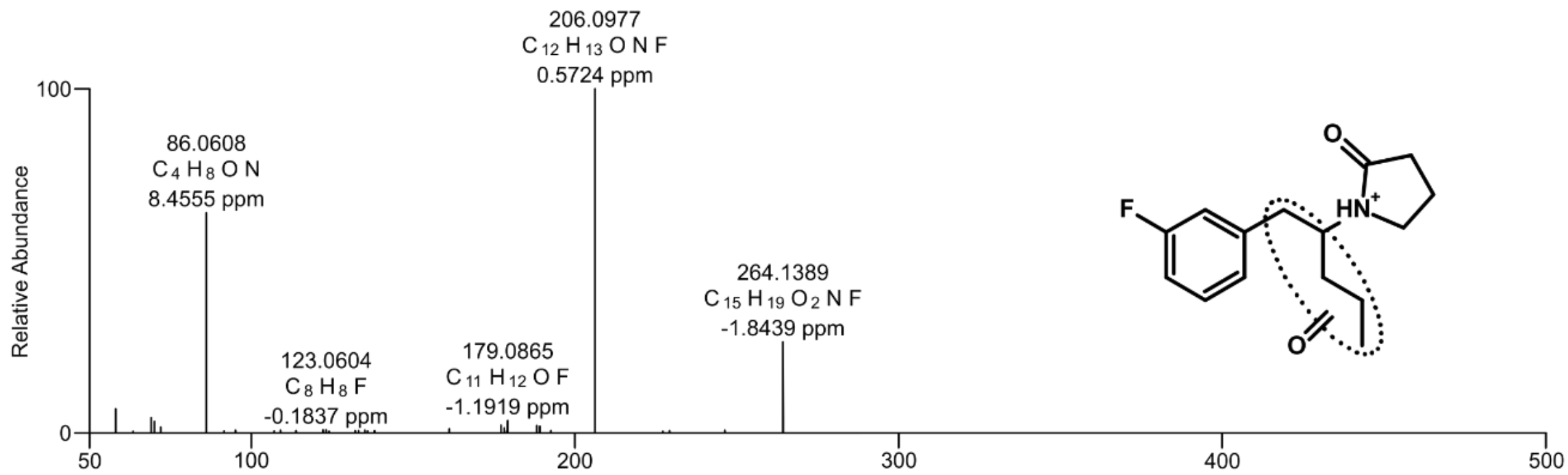

HRMS² of 3FM-9 (dioxo 3-fluoroprolintane, *m/z* at 264.1394, RT 6.79 min)

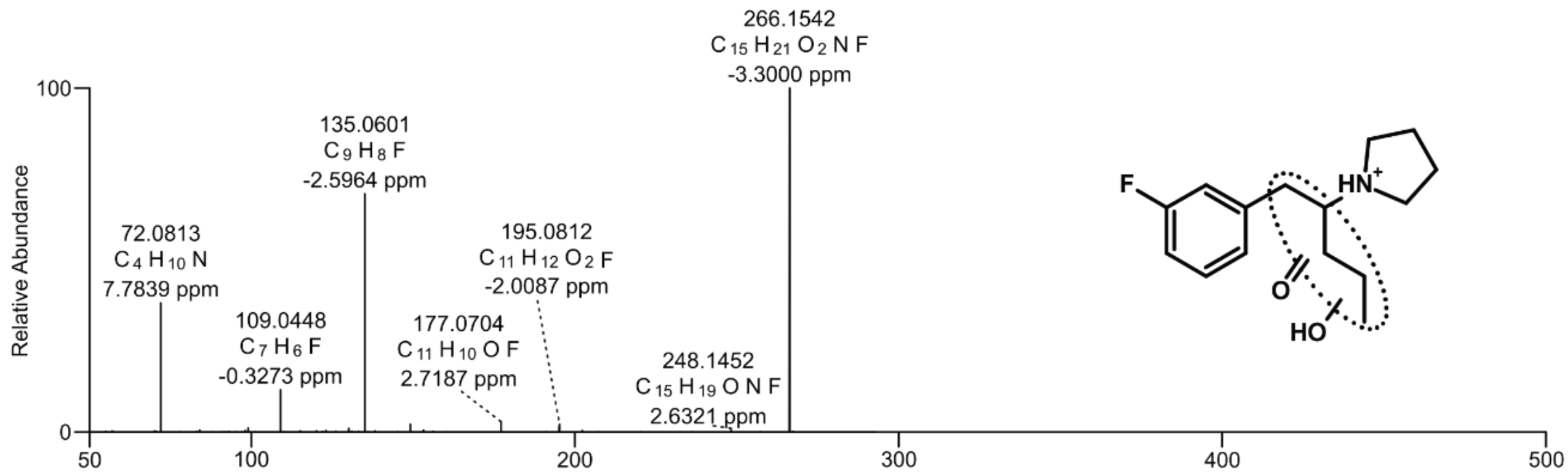

HRMS² of 3FM-10 (oxo hydroxy 3-fluoroprolintane isomer 1, *m/z* at 266.1551, RT 4.32 min)

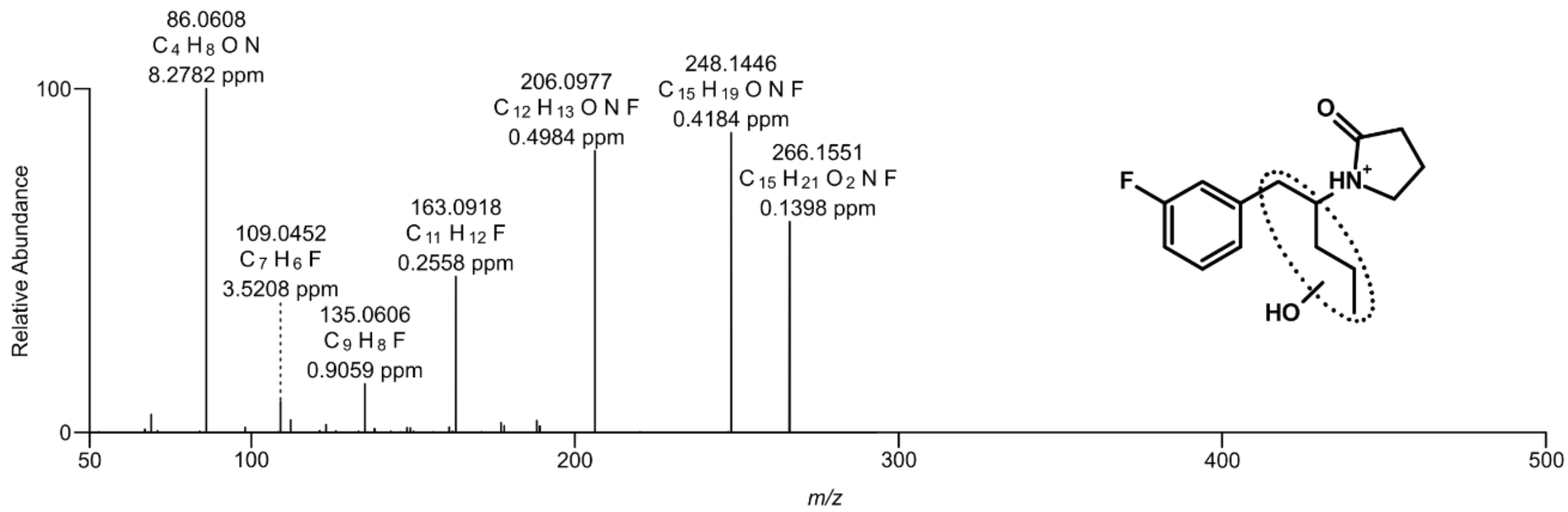

HRMS² of 3FM-11 (oxo hydroxy 3-fluoroprolintane isomer 2, *m/z* at 266.1551, RT 6.57 min)

Figure S2 (continued)

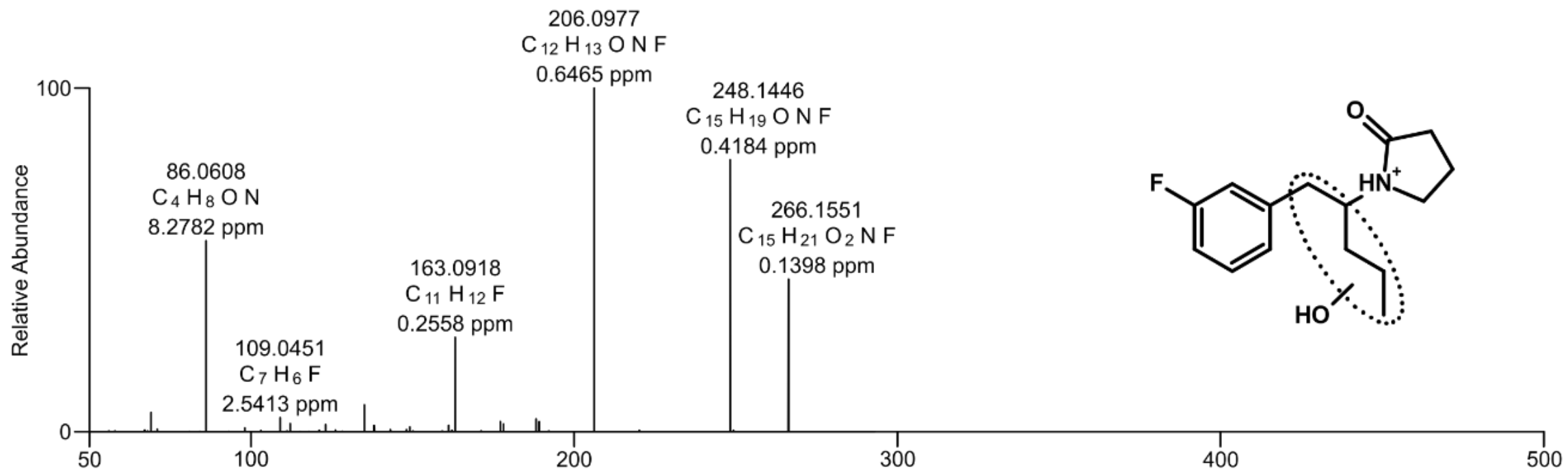

HRMS² of 3FM-12 (oxo hydroxy 3-fluoroprolintane isomer 3, *m/z* at 266.1551, RT 6.92 min)

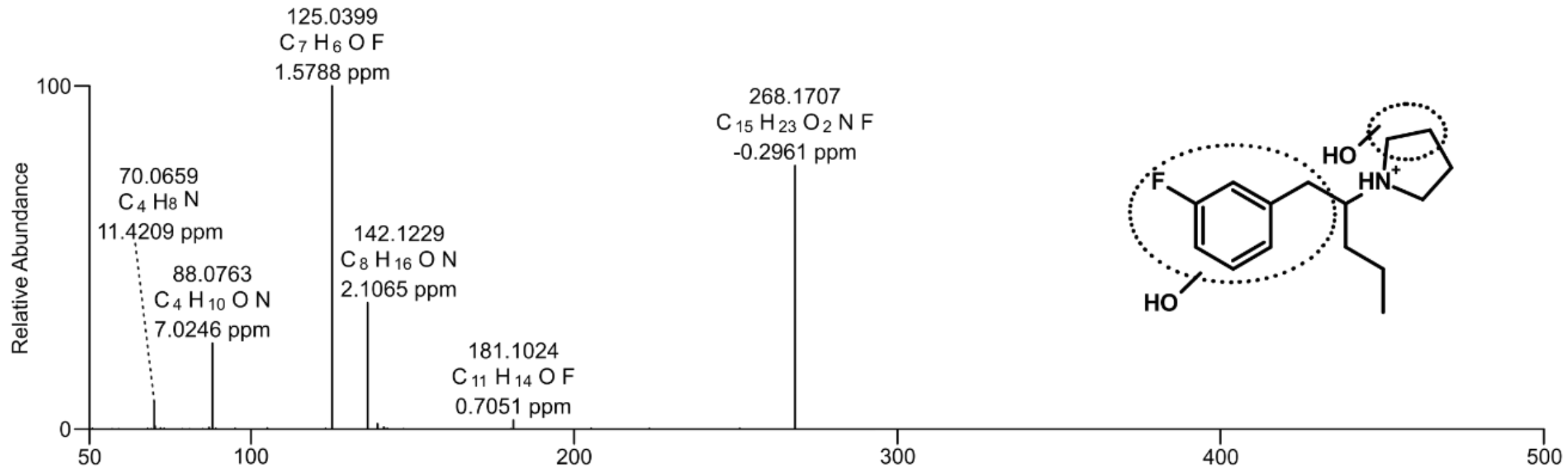

HRMS² of 3FM-13 (dihydroxy 3-fluoroprolintane isomer 1, *m/z* at 268.1707, RT 4.09 min)

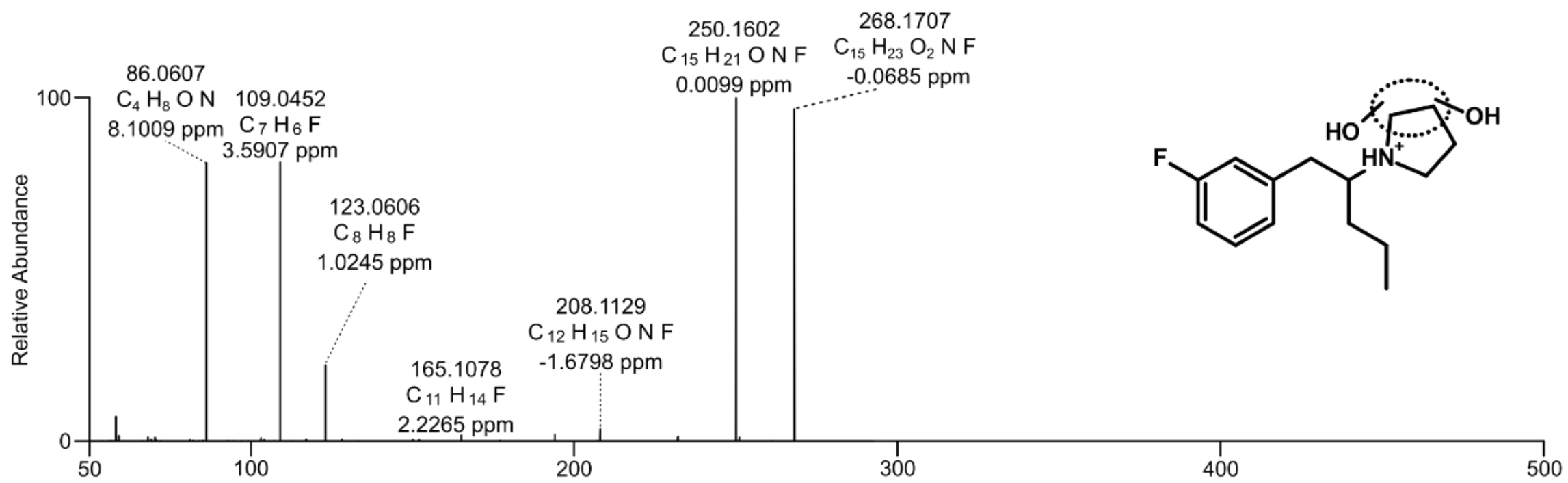

HRMS² of 3FM-14 (dihydroxy 3-fluoroprolintane isomer 2, *m/z* at 268.1707, RT 5.35 min)

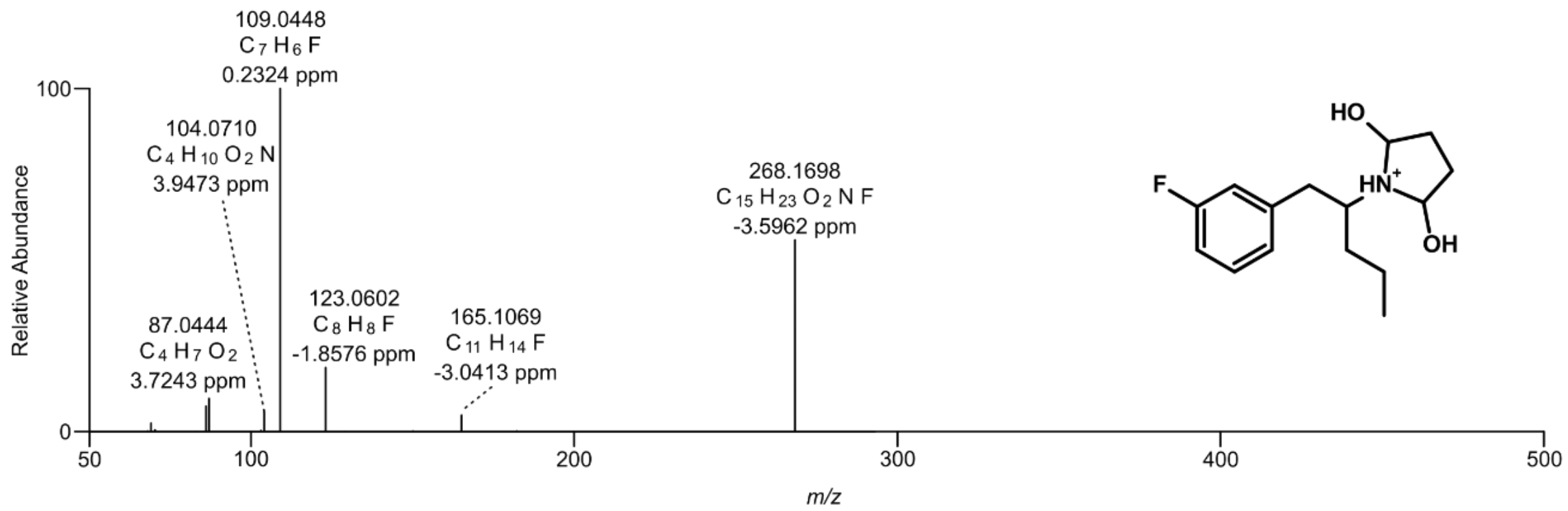

HRMS² of 3FM-15 (dihydroxy 3-fluoroprolintane isomer 3, *m/z* at 268.1707, RT 5.81 min)

Figure S2 (continued)

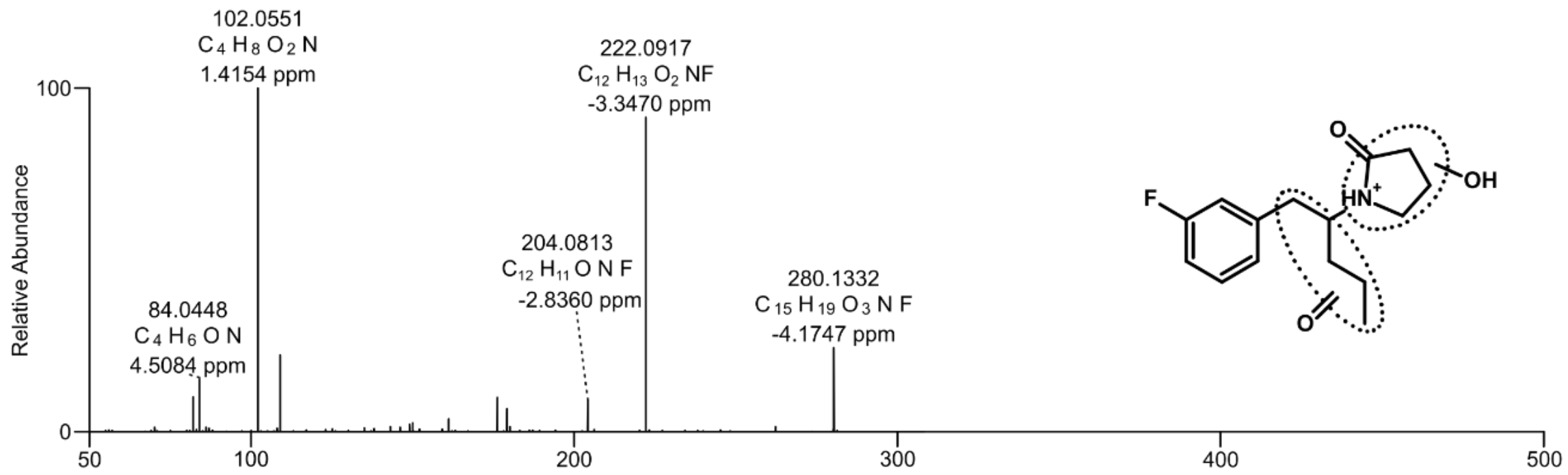

HRMS² of 3FM-16 (dioxo hydroxy 3-fluoroprolintane, *m/z* at 280.1343, RT 5.66 min)

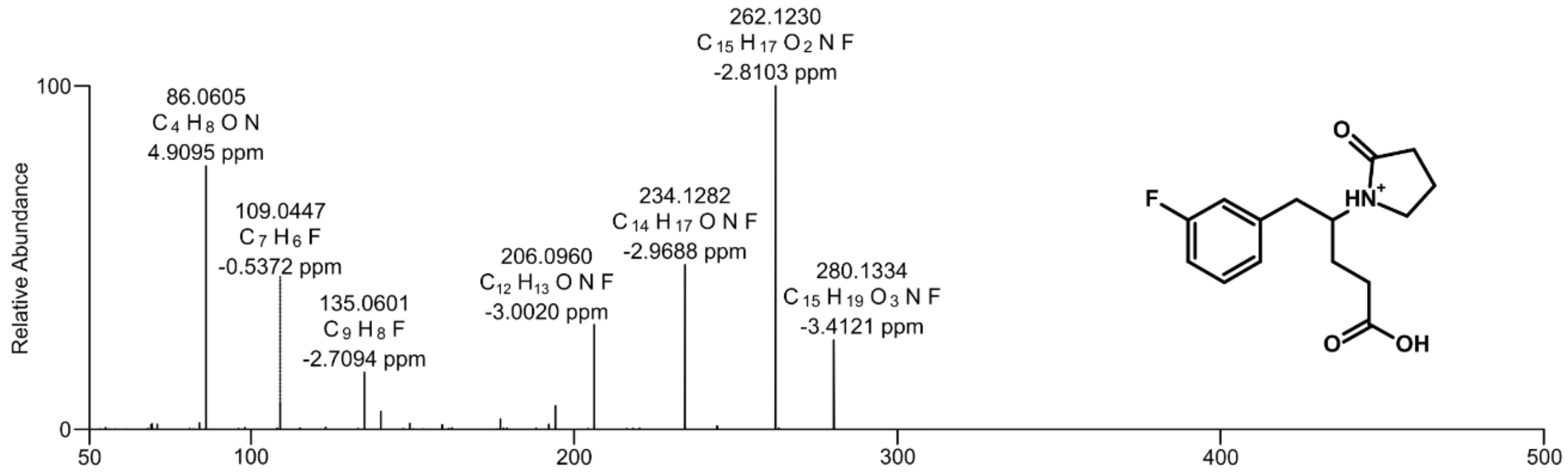

HRMS² of 3FM-17 (oxo 3-fluoroprolintane carboxylic acid, *m/z* at 280.1343, RT 6.76 min)

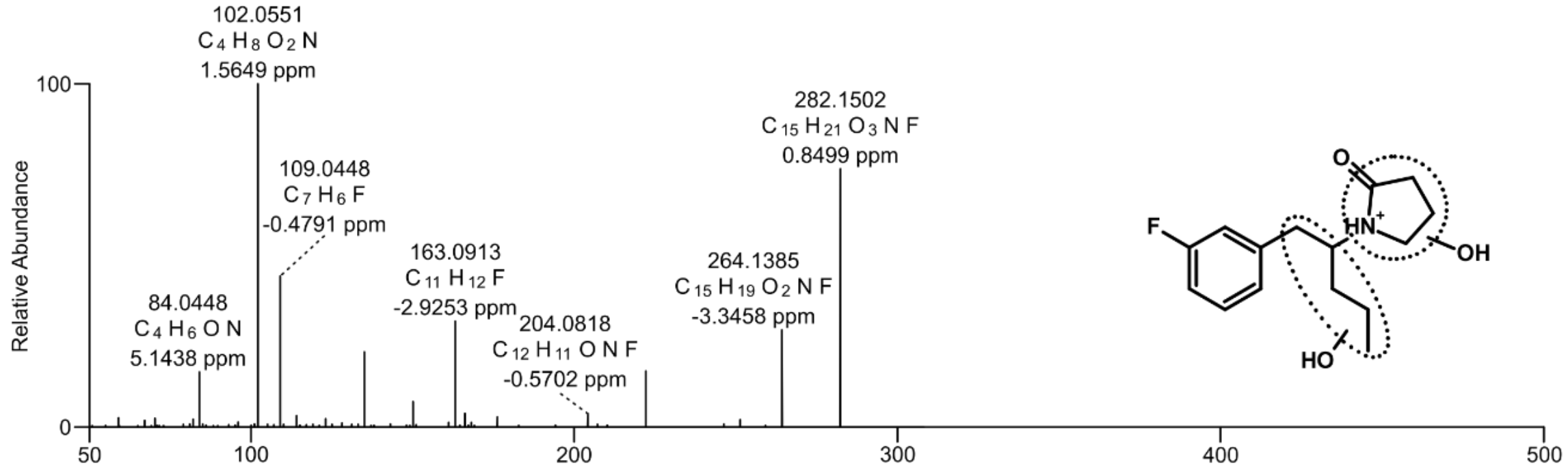

HRMS² of 3FM-18 (oxo dihydroxy 3-fluoroprolintane isomer 1, *m/z* at 282.1500, RT 5.39 min)

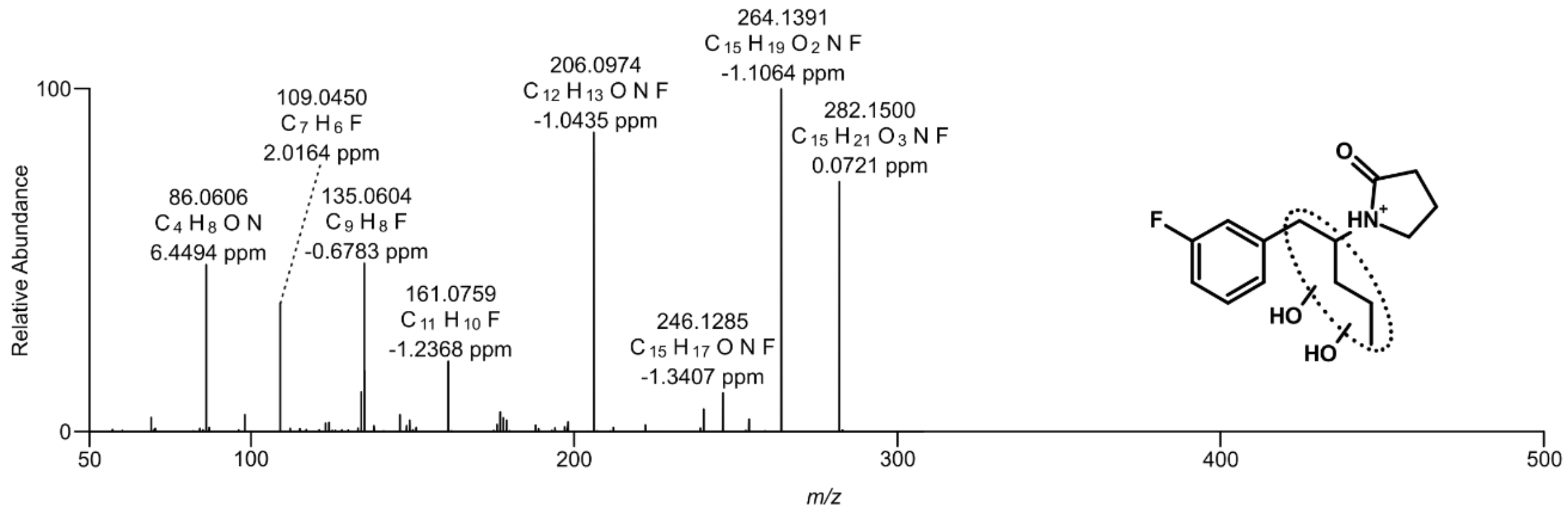

HRMS² of 3FM-19 (oxo dihydroxy 3-fluoroprolintane isomer 2, *m/z* at 282.1500, RT 5.89 min)

Figure S2 (continued)

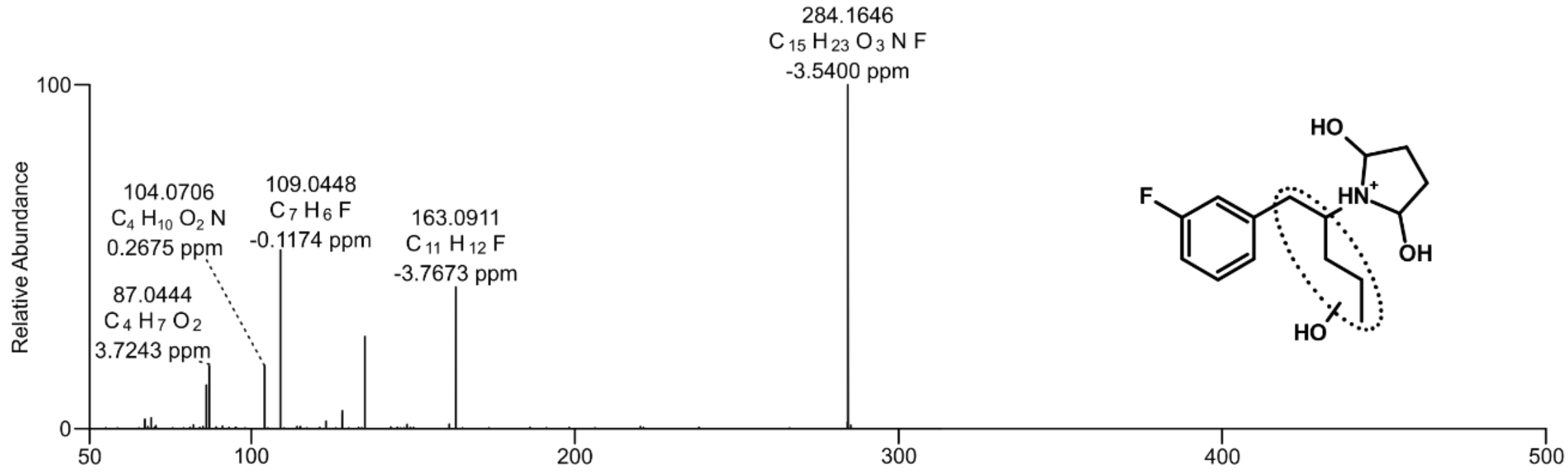

HRMS² of 3FM-20 (trihydroxy 3-fluoroprolintane, *m/z* at 284.1656, RT 4.25 min)

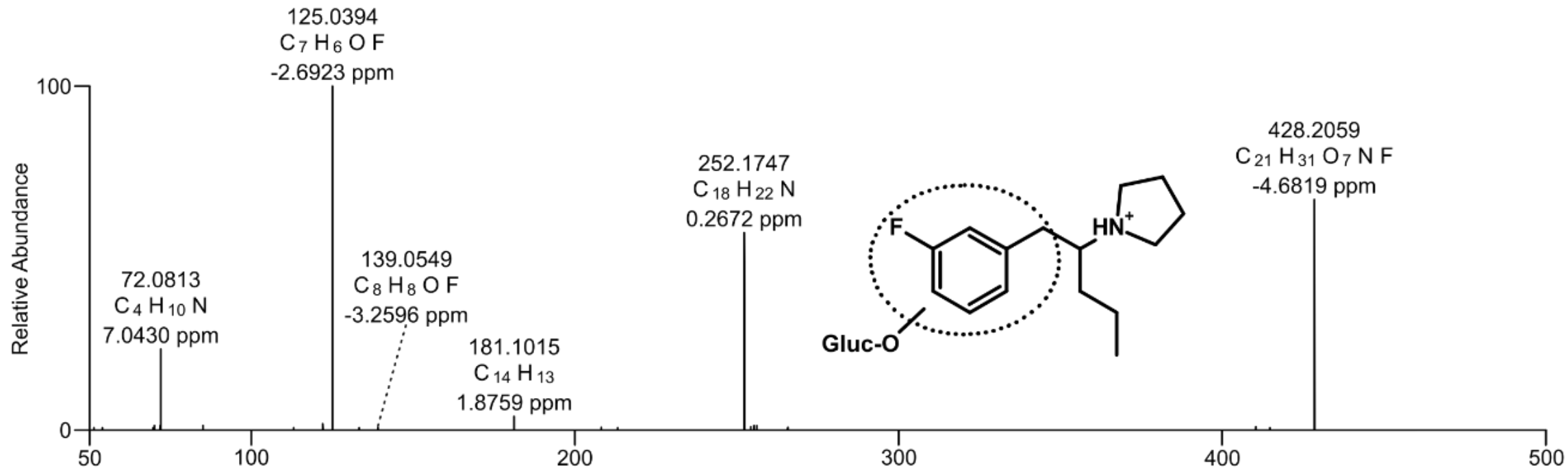

HRMS² of 3FM-21 (hydroxy 3-fluoroprolintane glucuronide isomer 1, *m/z* at 428.2079, RT 3.52 min)

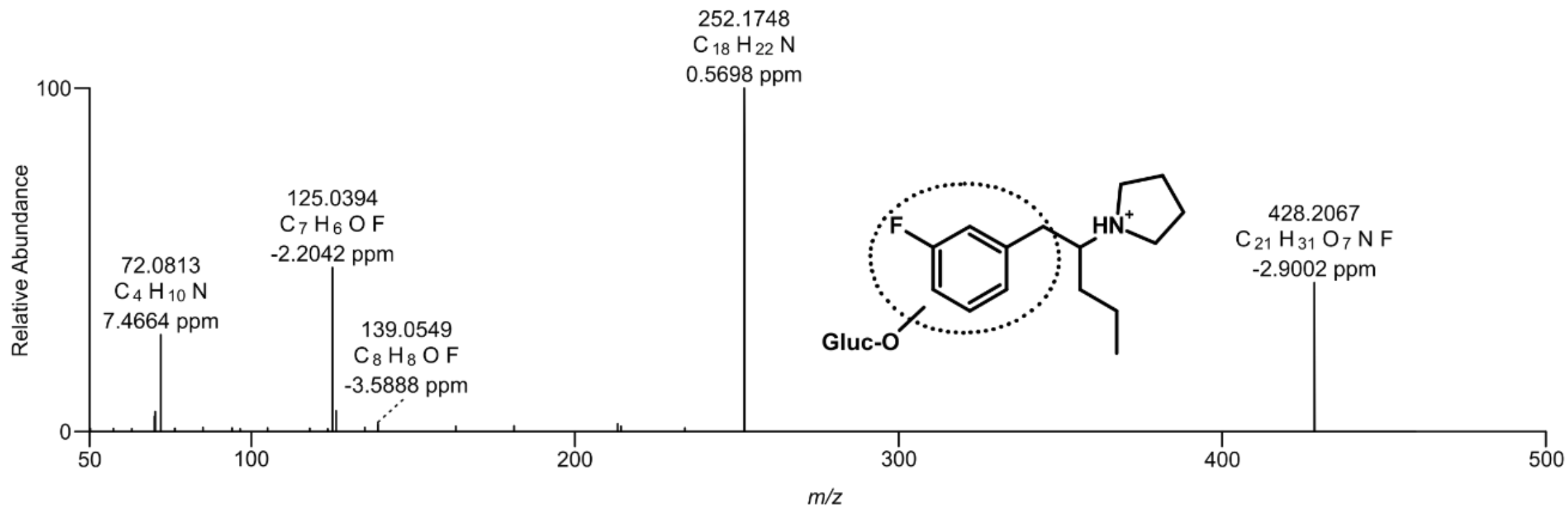

HRMS² of 3FM-22 (hydroxy 3-fluoroprolintane glucuronide isomer 2, *m/z* at 428.2079, RT 4.08 min)

Figure S2 (continued)

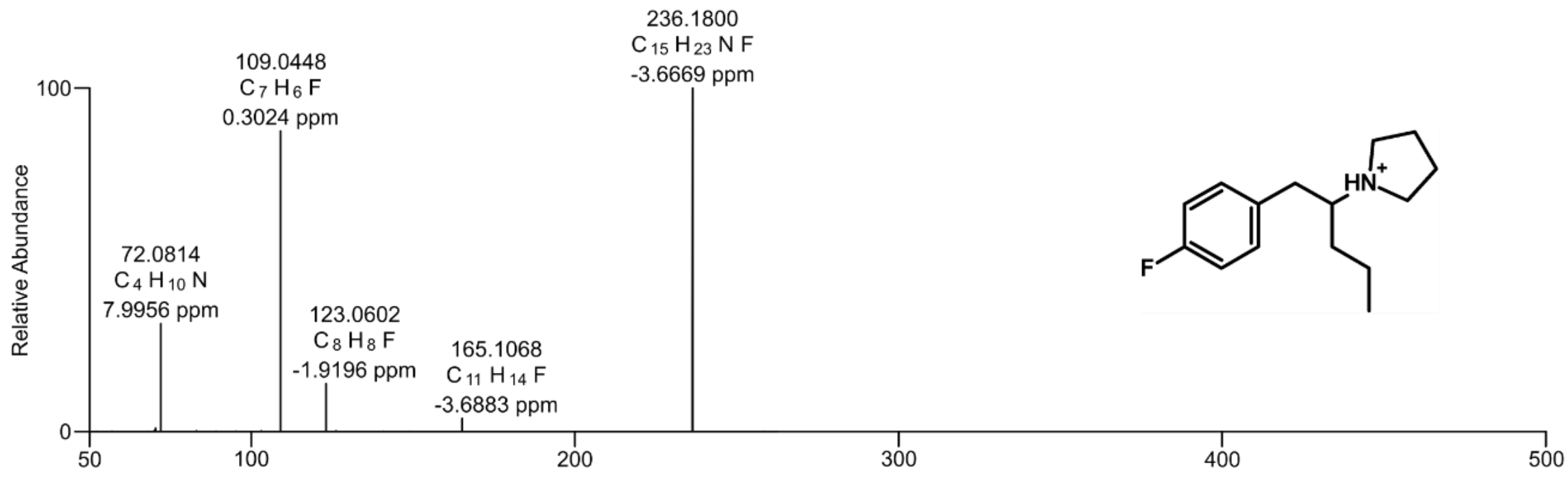

HRMS² of 4-fluoroprolintane (*m/z* at 236.1809, RT 5.72 min)

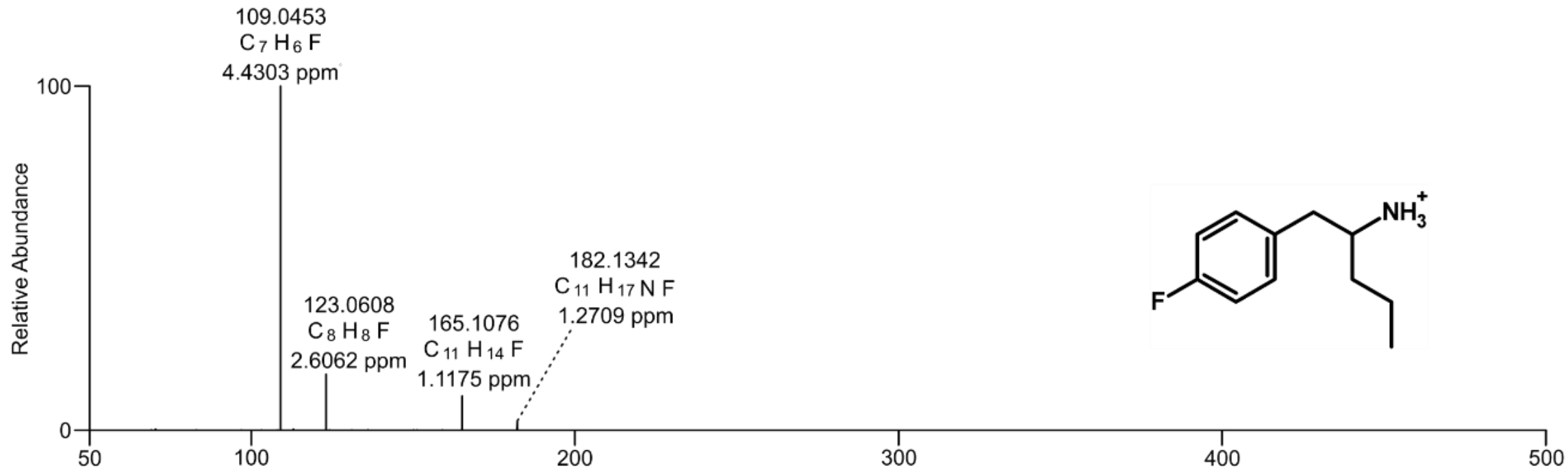

HRMS² of 4FM-1 (*N,N-bis*-dealkyl 4-fluoroprolintane, *m/z* at 182.1340, RT 5.18 min)

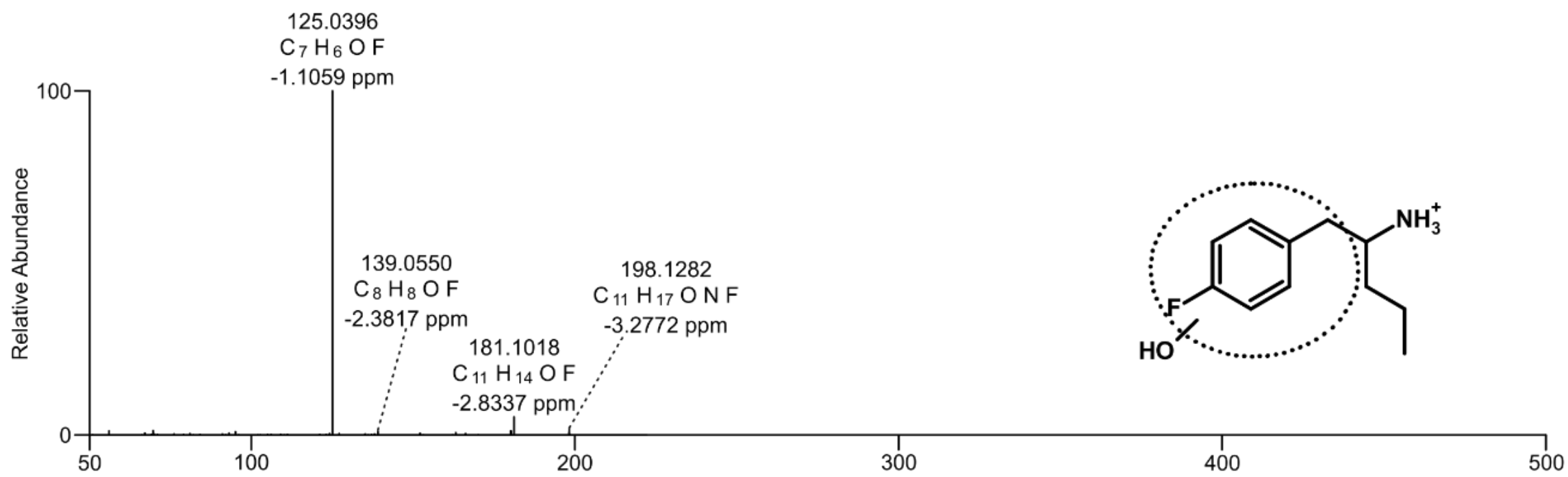

HRMS² of 4FM-2 (*N,N-bis*-dealkyl hydroxy 4-fluoroprolintane, *m/z* at 198.1289, RT 4.15 min)

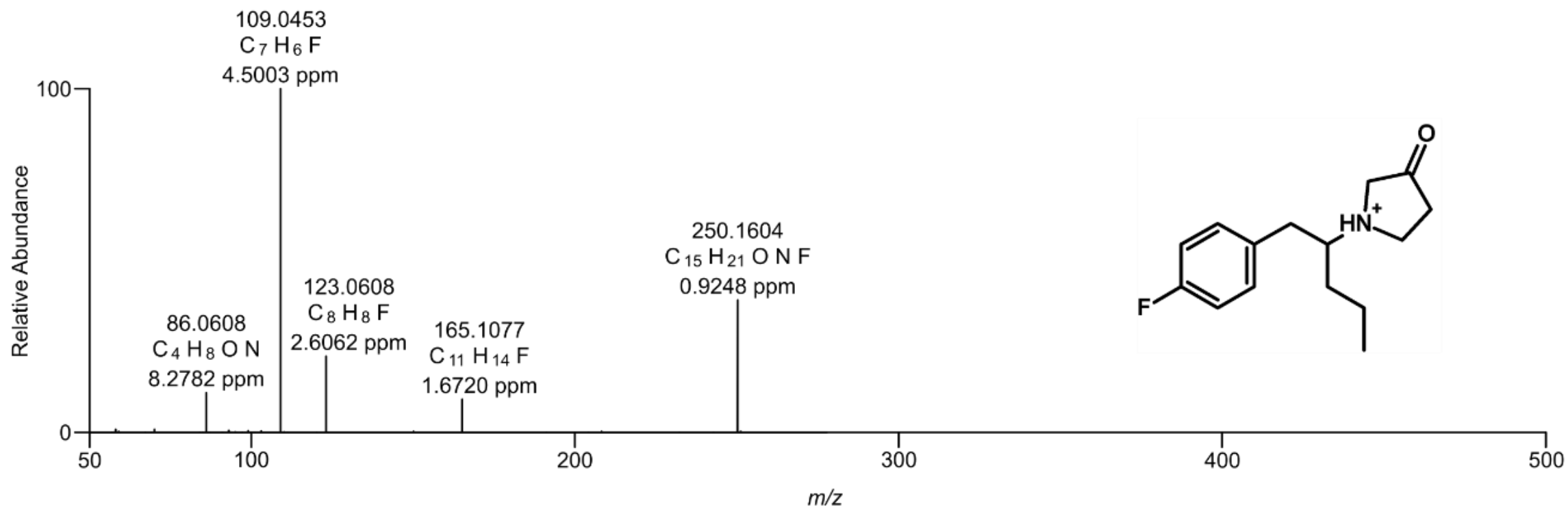

HRMS² of 4FM-3 (oxo 4-fluoroprolintane isomer 1, *m/z* at 250.1602, RT 5.41 min)

Figure S3 High-resolution (HR) MS^2 spectra and retention times (RT) of 4-fluoroprolintane and its metabolites detected in vivo and/or in vitro.

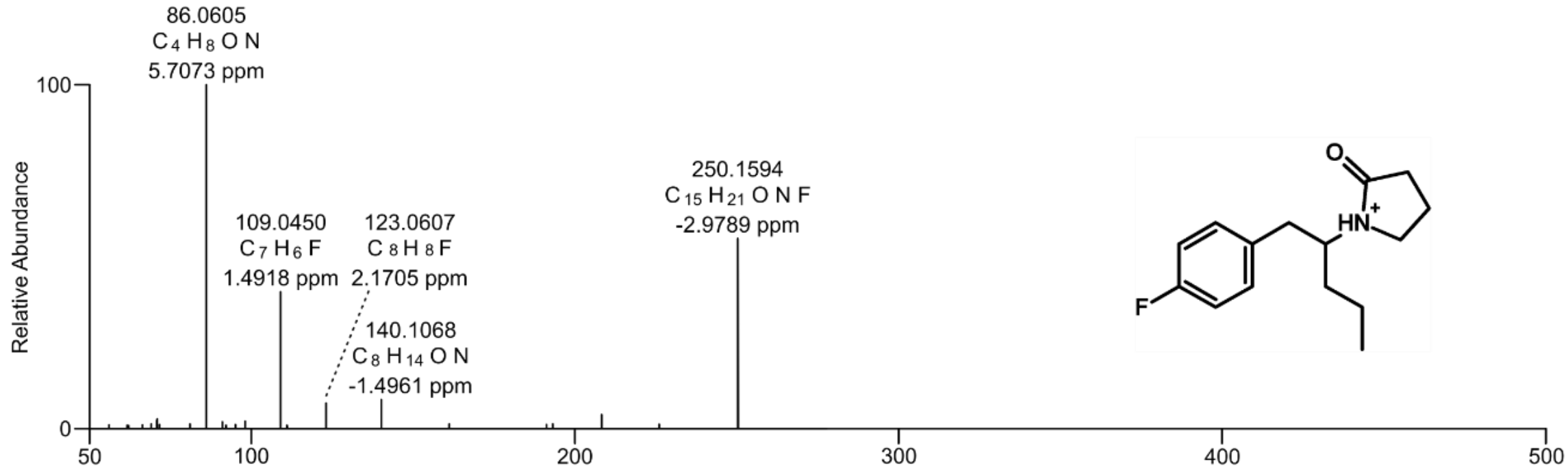

HRMS^2 of 4FM-4 (oxo 4-fluoroprolintane isomer 2, *m/z* at 250.1602, RT 8.65 min)

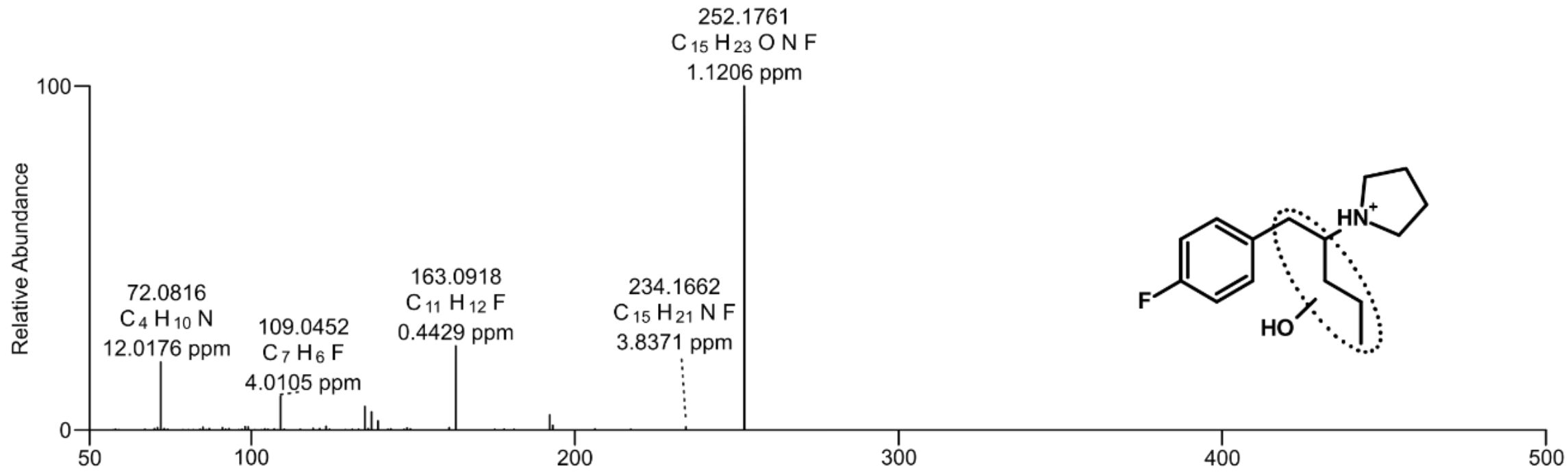

HRMS^2 of 4FM-5 (hydroxy 4-fluoroprolintane isomer 1, *m/z* at 252.1758, RT 4.62 min)

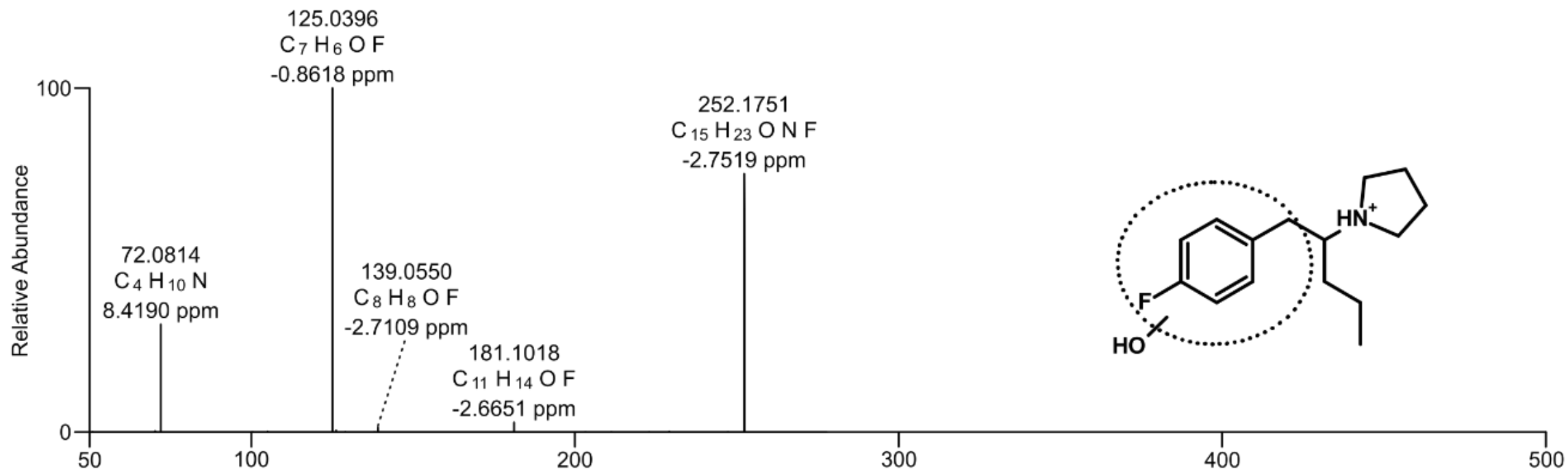

HRMS^2 of 4FM-6 (hydroxy 4-fluoroprolintane isomer 2, *m/z* at 252.1758, RT 4.63 min)

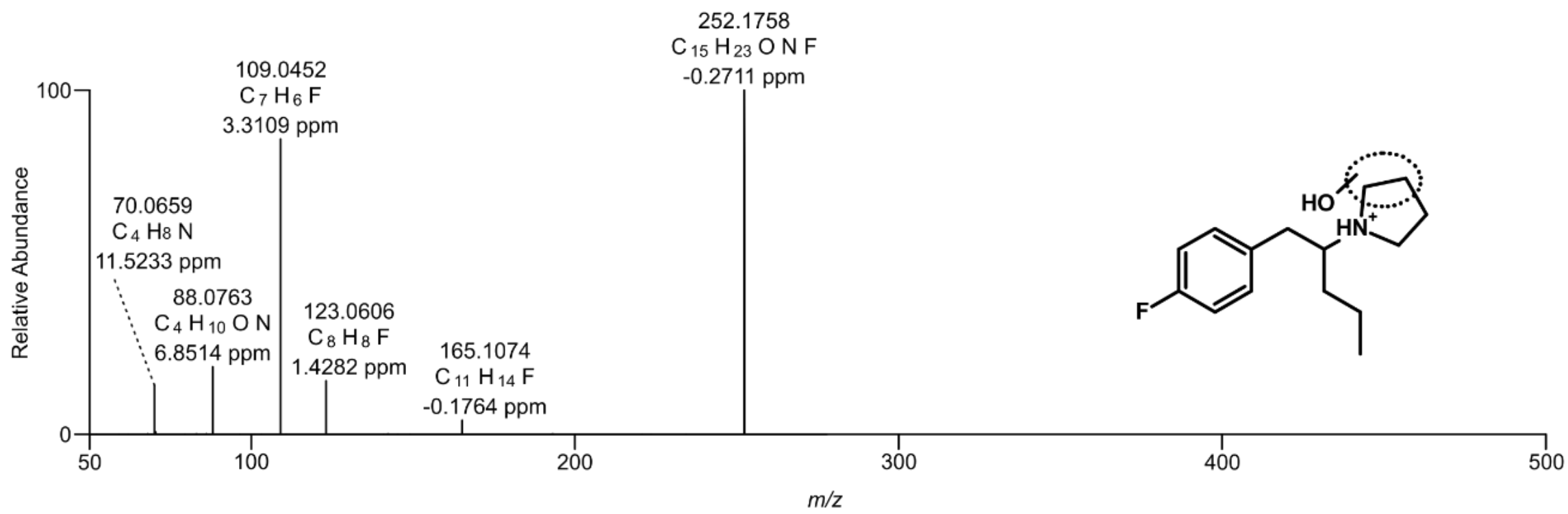

HRMS^2 of 4FM-7 (hydroxy 4-fluoroprolintane isomer 3, *m/z* at 252.1758, RT 5.46 min)

Figure S3 (continued)

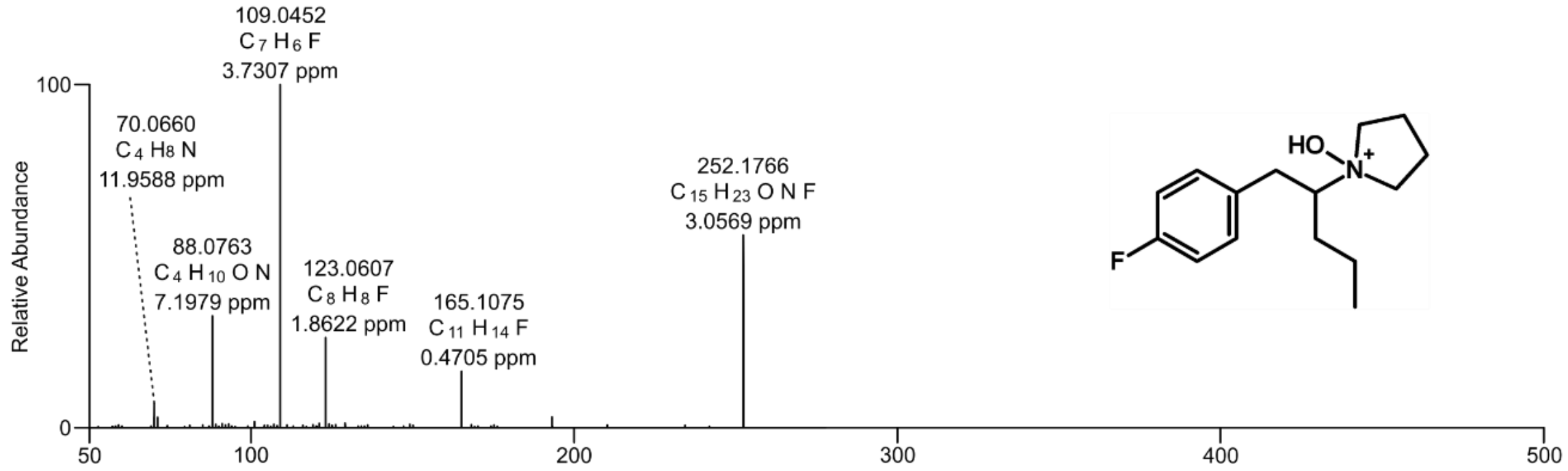

HRMS² of 4FM-8 (4-fluoroprolintane *N*-oxide, *m/z* at 252.1758, RT 6.17 min)

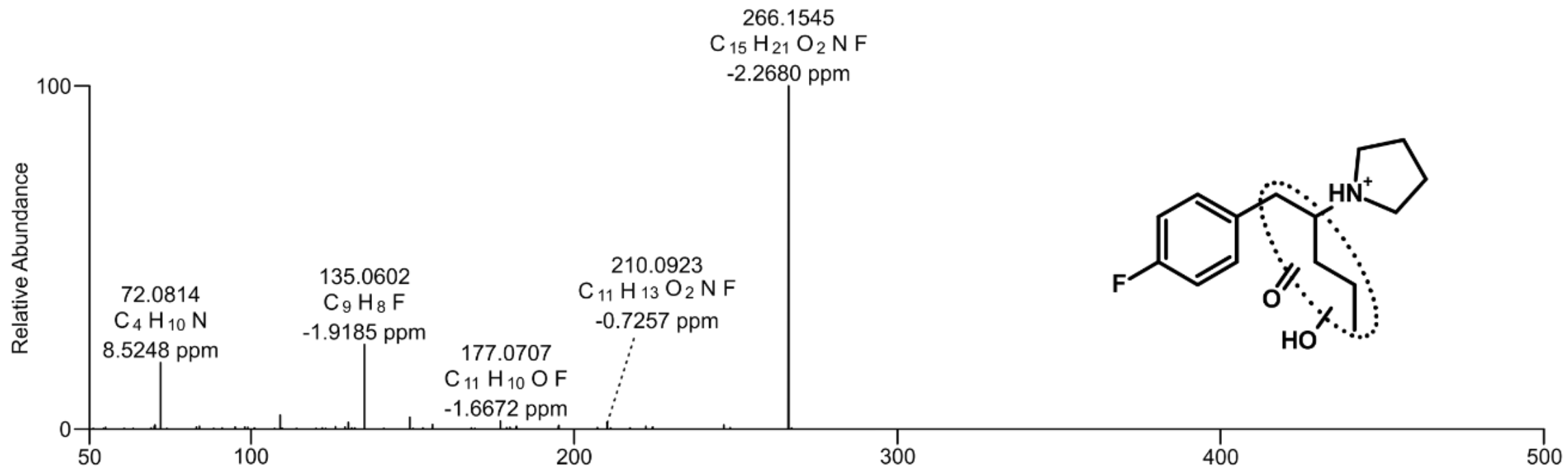

HRMS² of 4FM-9 (oxo hydroxy 4-fluoroprolintane isomer 1, *m/z* at 266.1551, RT 4.28 min)

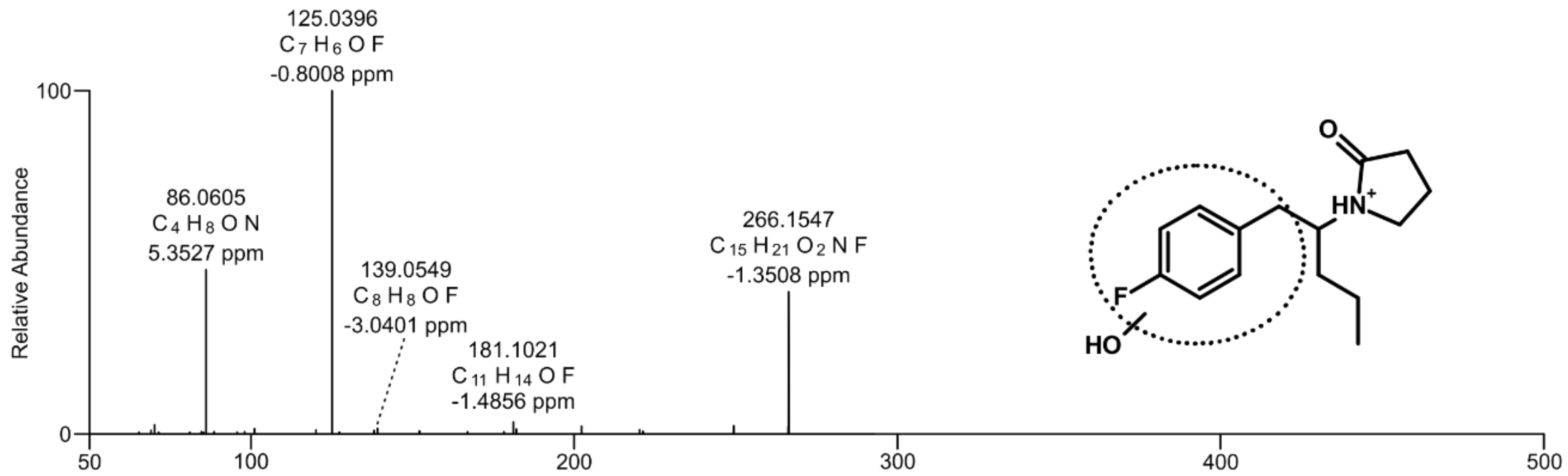

HRMS² of 4FM-10 (oxo hydroxy 4-fluoroprolintane isomer 2, *m/z* at 266.1551, RT 5.81 min)

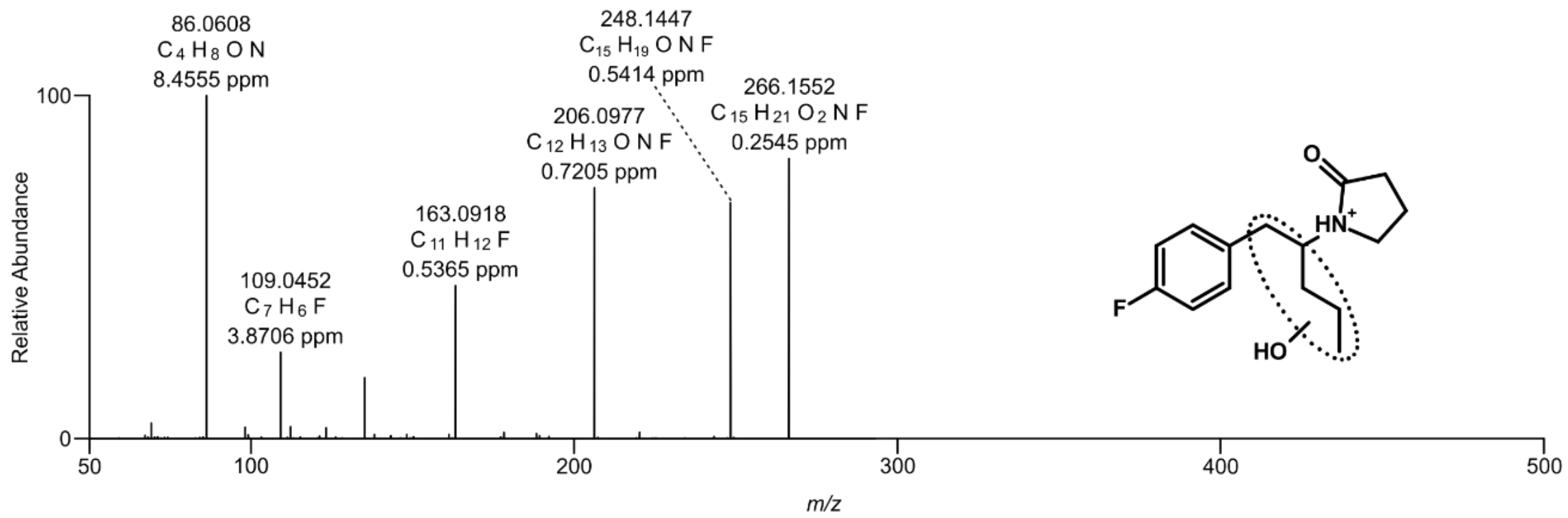

HRMS² of 4FM-11 (oxo hydroxy 4-fluoroprolintane isomer 3, *m/z* at 266.1551, RT 6.61 min)

Figure S3 (continued)

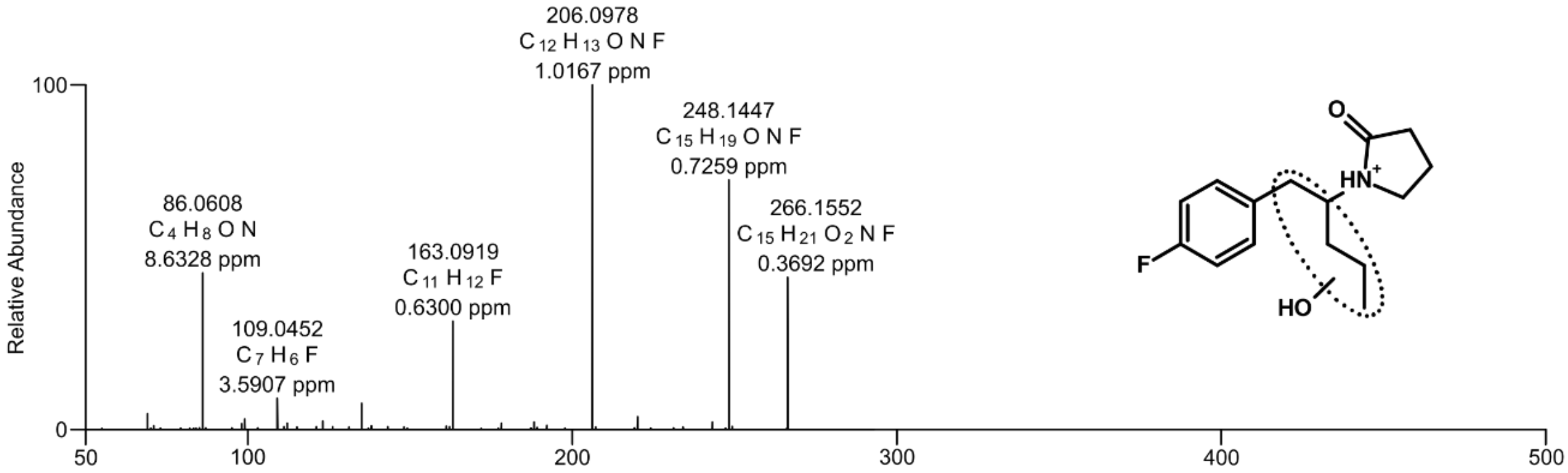

HRMS² of 4FM-12 (oxo hydroxy 4-fluoroprolintane isomer 4, *m/z* at 266.1551, RT 6.98 min)

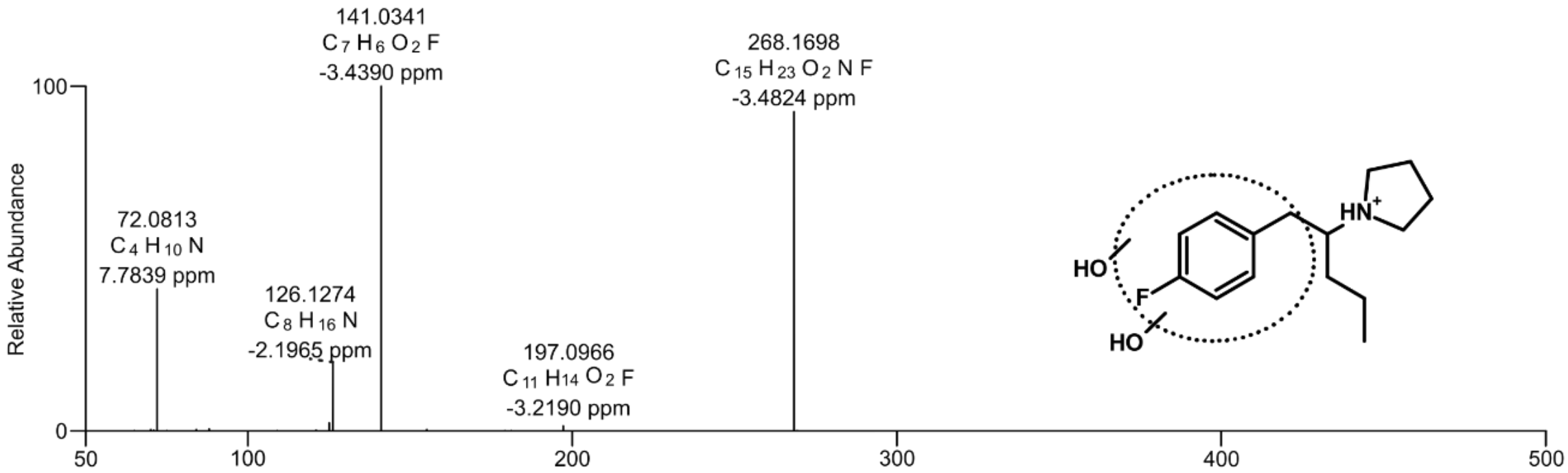

HRMS² of 4FM-13 (dihydroxy 4-fluoroprolintane isomer 1, *m/z* at 268.1707, RT 4.16 min)

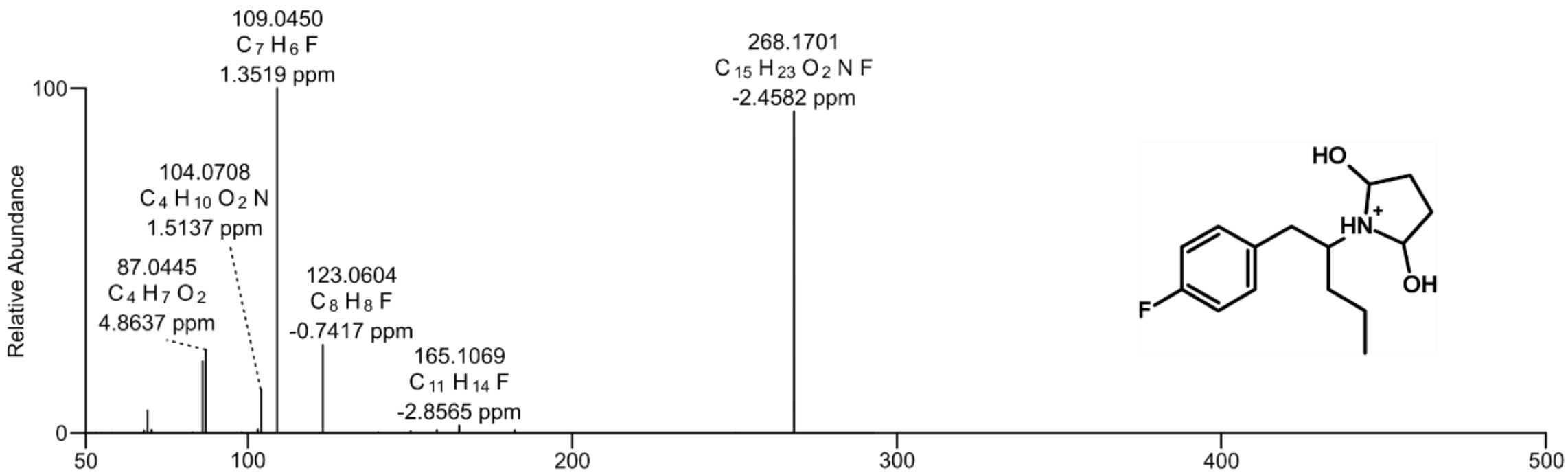

HRMS² of 4FM-14 (dihydroxy 4-fluoroprolintane isomer 2, *m/z* at 268.1707, RT 5.82 min)

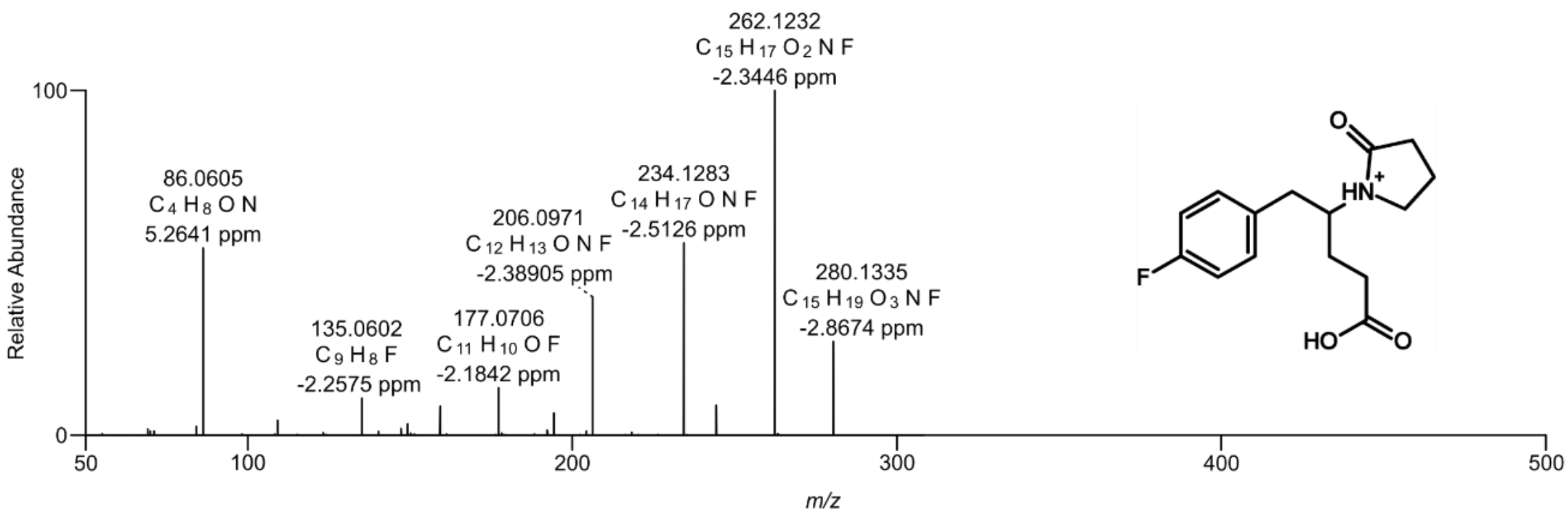

HRMS² of 4FM-15 (oxo 4-fluoroprolintane carboxylic acid, *m/z* at 280.1343, RT 6.75 min)

Figure S3 (continued)

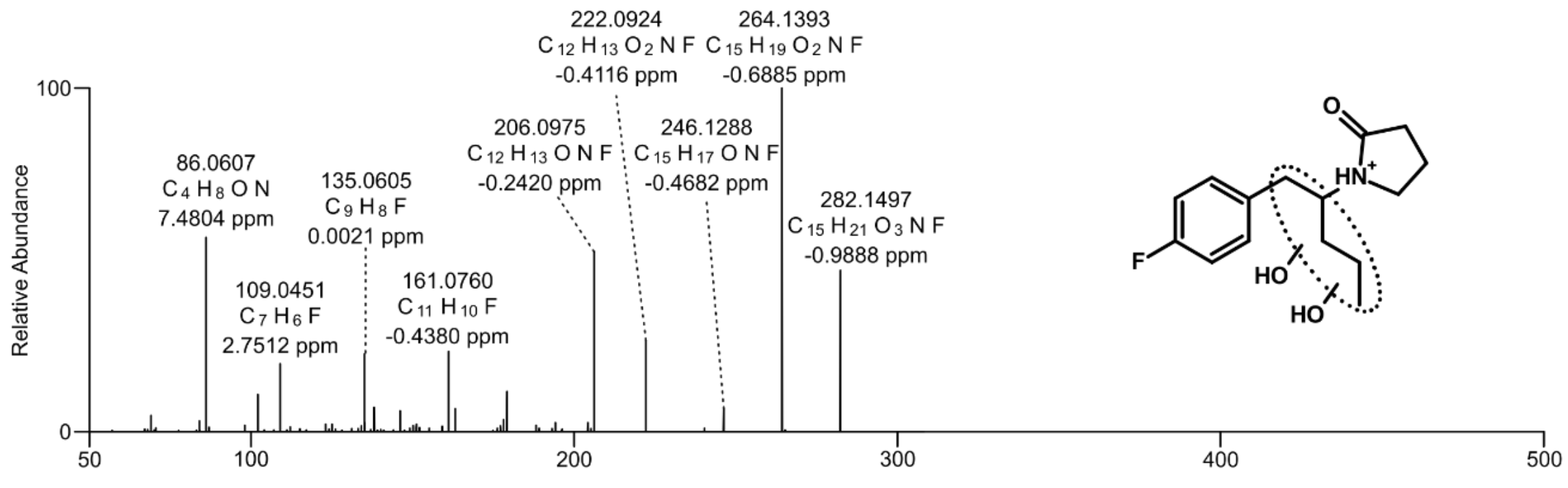

HRMS² of 4FM-16 (oxo dihydroxy 4-fluoroprolintane, *m/z* at 282.1500, RT 5.87 min)

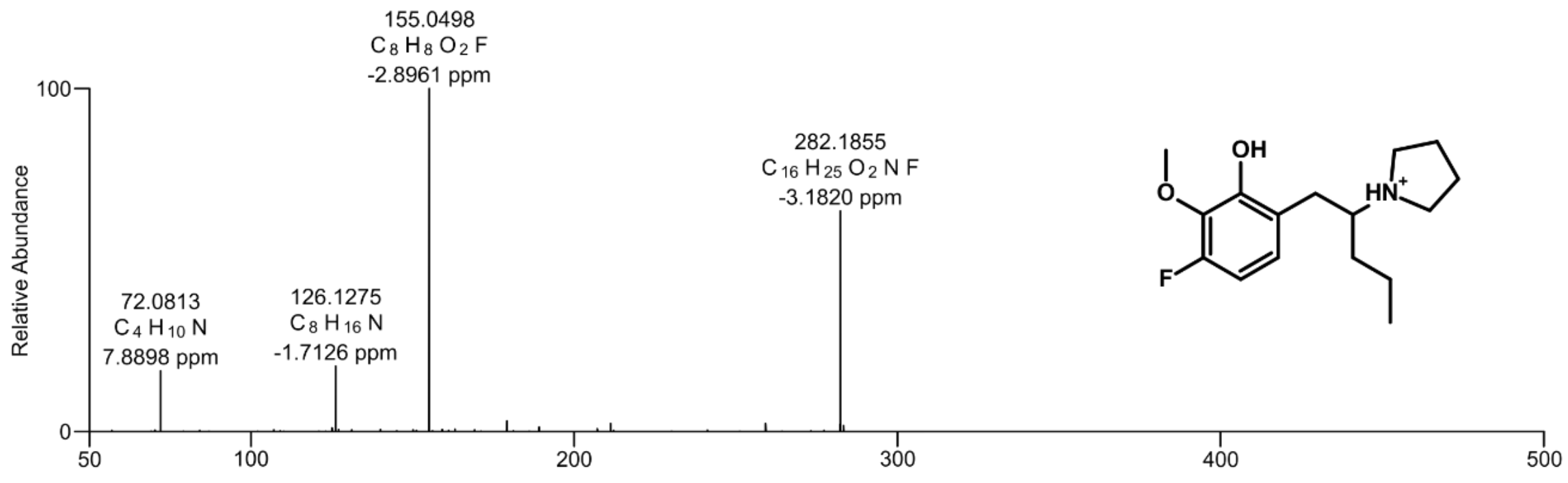

HRMS² of 4FM-17 (hydroxy methoxy 4-fluoroprolintane, *m/z* at 282.1864, RT 4.77 min)

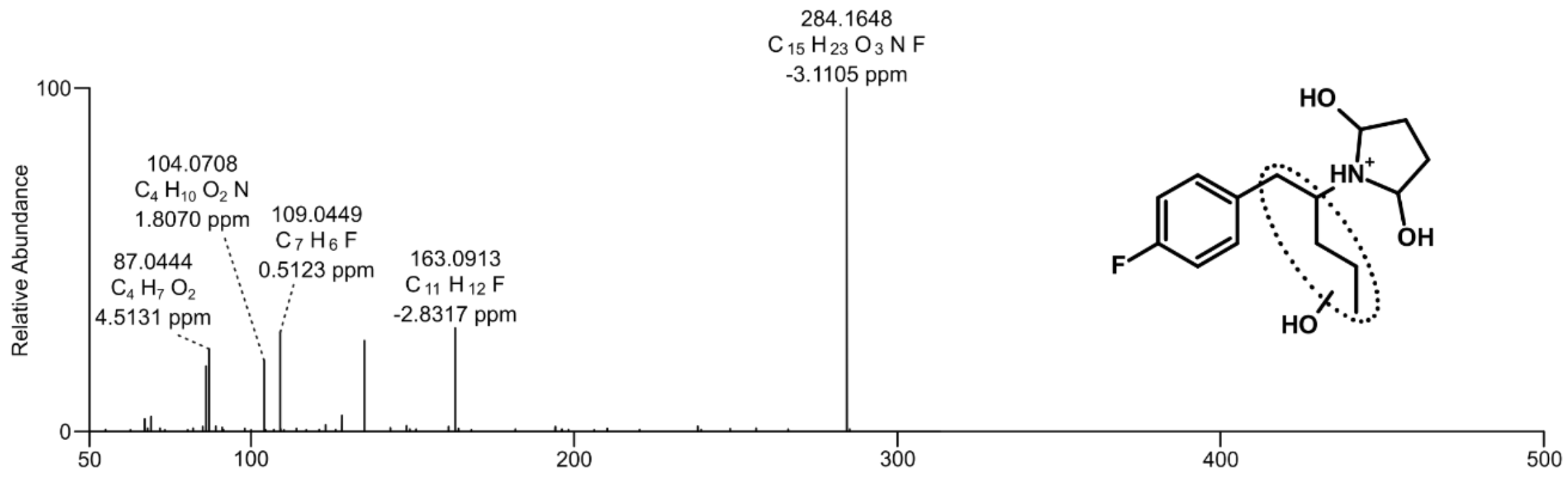

HRMS² of 4FM-18 (trihydroxy 4-fluoroprolintane isomer 1, *m/z* at 284.1656, RT 4.26 min)

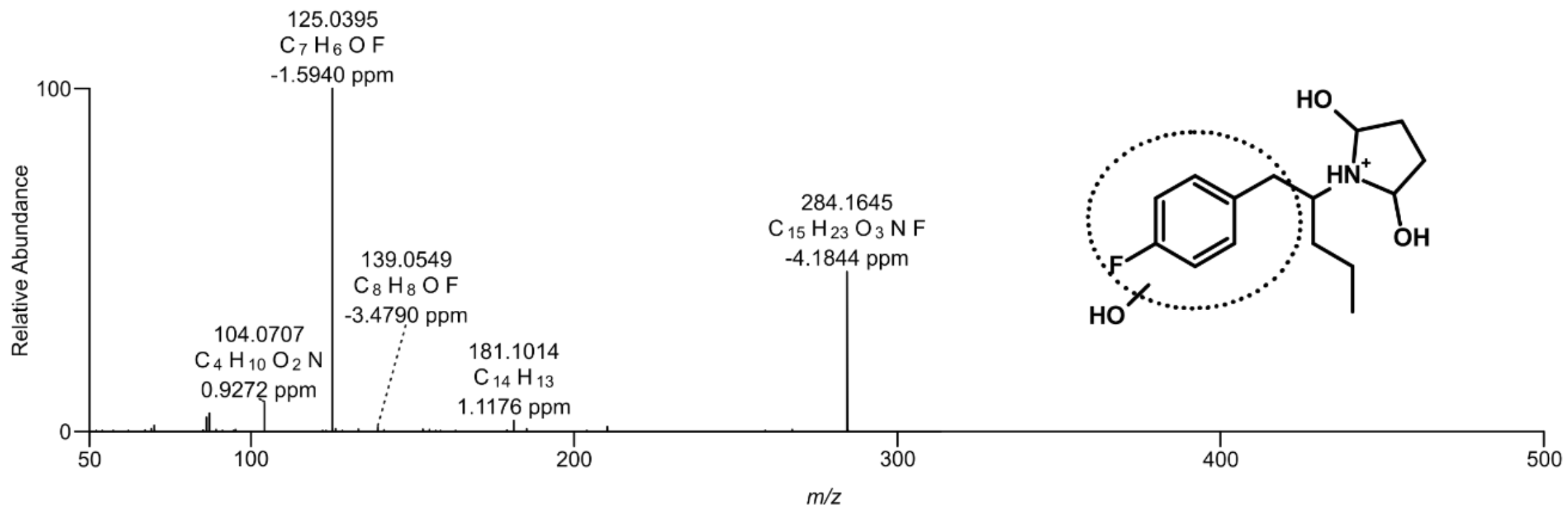

HRMS² of 4FM-19 (trihydroxy 4-fluoroprolintane isomer 2, *m/z* at 284.1656, RT 4.55 min)

Figure S3 (continued)

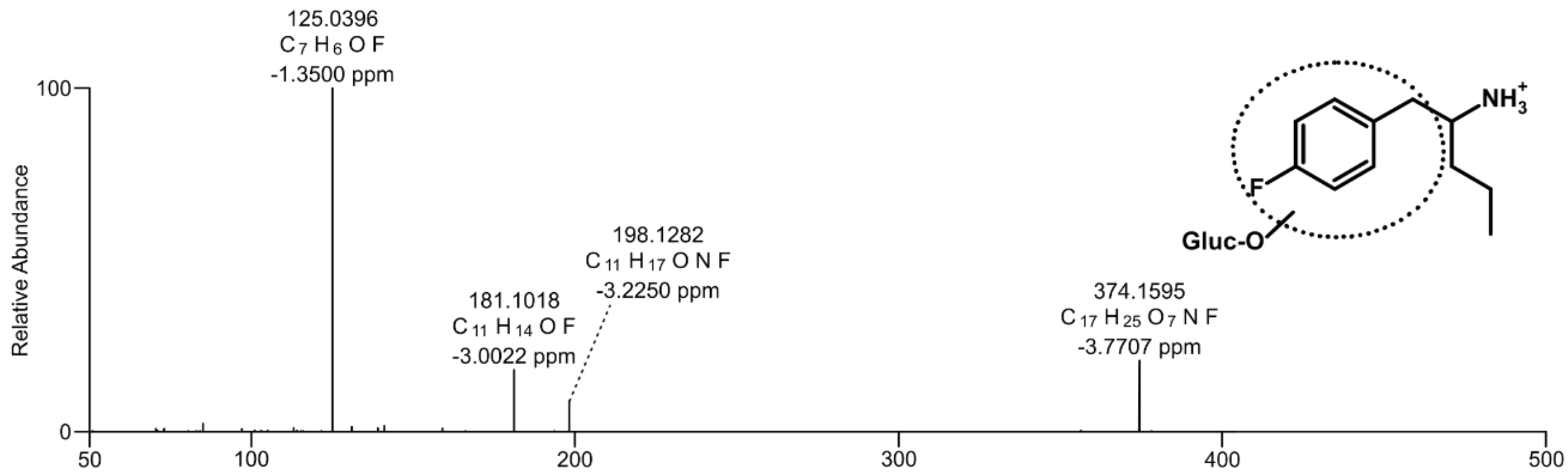

HRMS^2 of 4FM-20 (*N,N-bis*-dealkyl hydroxy 4-fluoroprolintane glucuronide, *m/z* at 374.1610, RT 3.22 min)

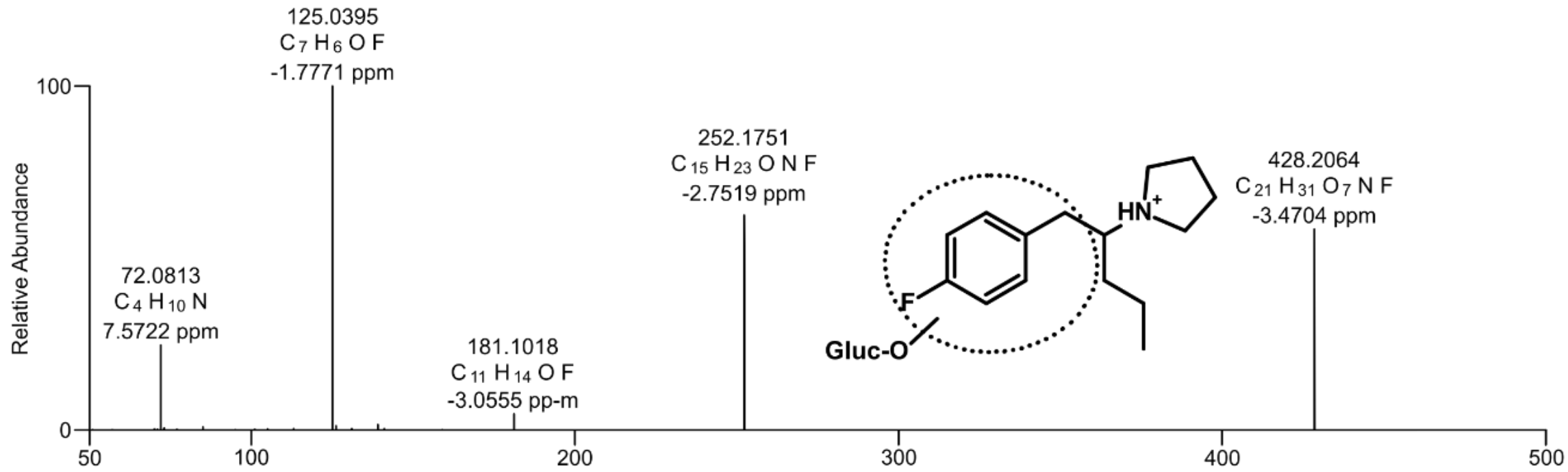

HRMS^2 of 4FM-21 (hydroxy 4-fluoroprolintane glucuronide, *m/z* at 428.2079, RT 3.51 min)

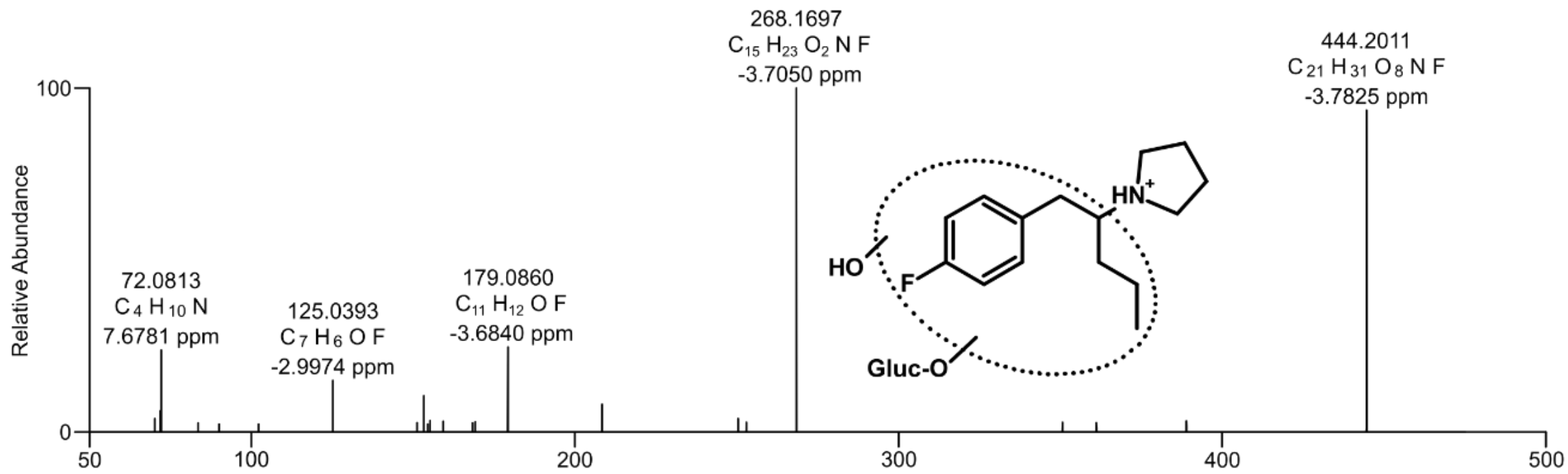

HRMS^2 of 4FM-22 (dihydroxy 4-fluoroprolintane glucuronide isomer 1, *m/z* at 444.2028, RT 2.42 min)

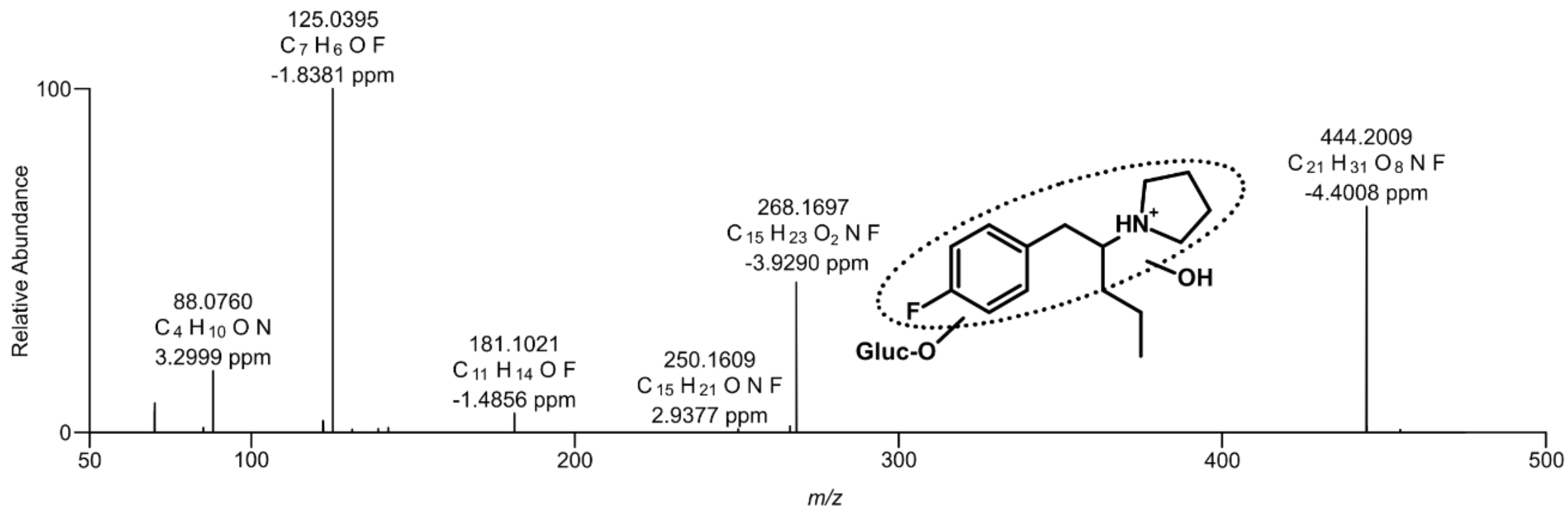

HRMS^2 of 4FM-23 (dihydroxy 4-fluoroprolintane glucuronide isomer 2, *m/z* at 444.2028, RT 3.13 min)

Figure S3 (continued)

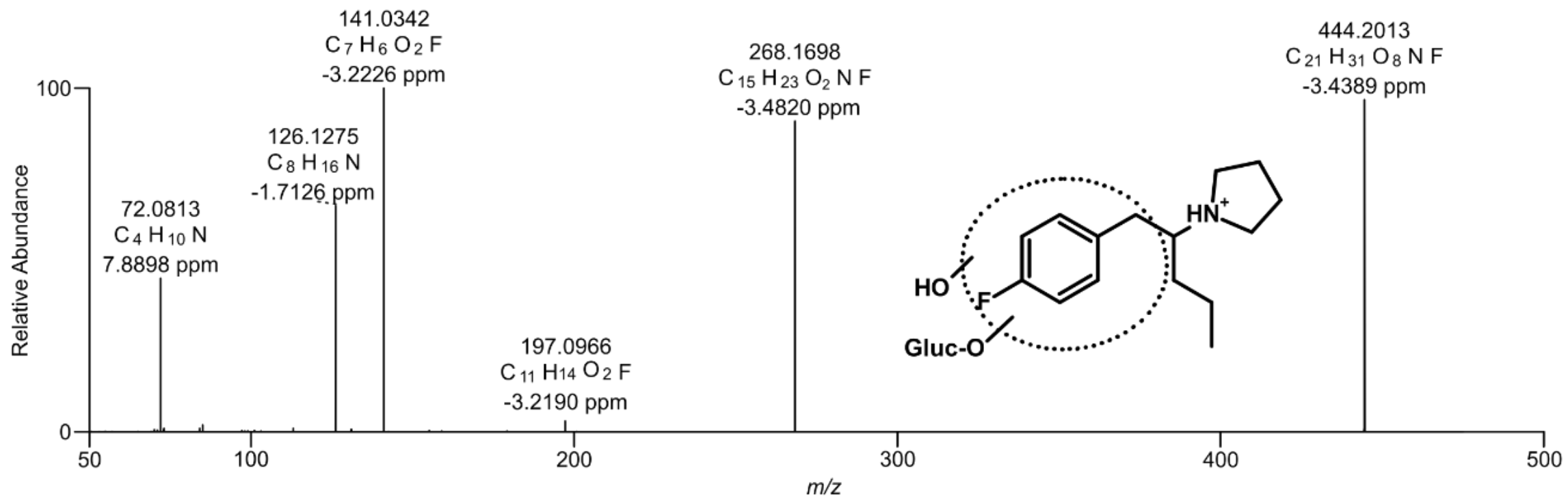

HRMS² of 4FM-24 (dihydroxy 4-fluoroprolintane glucuronide isomer 3, *m/z* at 444.2028, RT 3.61 min)

Figure S3 (continued)

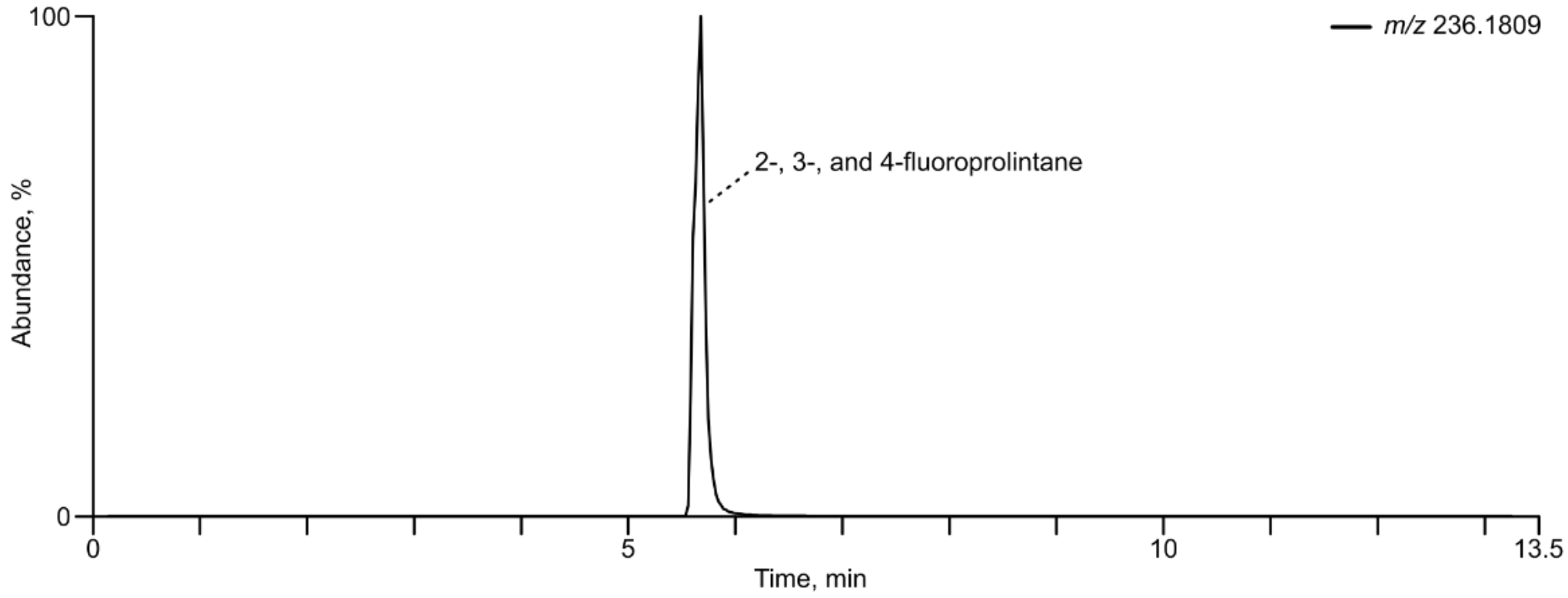

Figure S4 Extracted ion chromatograms of 2-, 3-, and 4-fluoroprolintane after reversed-phase liquid chromatography high-resolution mass spectrometry analysis of a pure sure substance solution (100 ng/mL).

TABLE S1 Detection of the 2-, 3-, and 4-fluoroprolintane metabolites in the given metabolism models. Metabolite IDs correspond to Figures 3-5. RU, rat urine; pHLS9, pooled human liver S9 fraction; MAS, monooxygenases activity screening; +, detected; -, not detected.

Metabolite ID	RU	pHLS9	MAS
2-Fluoroprolintane	+	+	+
2FM-1 (oxo-)	-	-	+
2FM-2 (oxo-)	+	+	+
2FM-3 (hydroxy-)	+	-	+
2FM-4 (hydroxy-)	+	+	+
2FM-5 (hydroxy-)	+	-	+
2FM-6 (hydroxy-)	+	+	+
2FM-7 (hydroxy-)	+	+	+
2FM-8 (*N*-oxide)	-	-	+
2FM-9 (ring open hydroxy-)	-	+	-
2FM-10 (dioxo-)	+	+	+
2FM-11 (oxo hydroxy-)	+	-	-
2FM-12 (oxo hydroxy-)	+	+	+
2FM-13 (oxo hydroxy-)	+	+	+
2FM-14 (dihydroxy-)	-	-	+
2FM-15 (dihydroxy-)	+	-	-
2FM-16 (dihydroxy-)	+	-	+
2FM-17 (dihydroxy-)	+	-	+
2FM-18 (dihydroxy-)	-	-	+
2FM-19 (dihydroxy-)	-	-	+
2FM-20 (dihydroxy-)	+	+	+
2FM-21 (dioxo hydroxy-)	+	-	-
2FM-22 (oxo carboxylic acid-)	+	-	-
2FM-23 (oxo dihydroxy-)	+	-	-
2FM-24 (oxo dihydroxy-)	+	-	-
2FM-25 (hydroxy methoxy-)	+	-	-
2FM-26 (trihydroxy-)	+	-	-
2FM-27 (trihydroxy-)	+	-	-
2FM-28 (trihydroxy-)	+	-	-
2FM-29 (oxo hydroxy methoxy-)	+	-	-
2FM-30 (*N,N-bis*-dealkyl hydroxy glucuronide-)	+	-	-
2FM-31 (hydroxy glucuronide-)	+	-	-
2FM-32 (oxo hydroxy glucuronide-)	-	+	-
2FM-33 (dihydroxy glucuronide-)	+	-	-
3-Fluoroprolintane	+	+	+
3FM-1 (*N,N-bis*-dealkyl-)	+	-	+
3FM-2 (*N,N-bis*-dealkyl hydroxy-)	+	-	-
3FM-3 (oxo-)	-	-	+
3FM-4 (oxo-)	+	+	+
3FM-5 (hydroxy-)	+	+	+
3FM-6 (hydroxy-)	+	-	+
3FM-7 (*N*-oxide)	+	-	+
3FM-8 (ring open hydroxy-)	-	+	-
3FM-9 (dioxo-)	+	+	+
3FM-10 (oxo hydroxy-)	+	-	-
3FM-11 (oxo hydroxy-)	+	+	+
3FM-12 (oxo hydroxy-)	+	+	+
3FM-13 (dihydroxy-)	-	-	+
3FM-14 (dihydroxy-)	-	-	+
3FM-15 (dihydroxy-)	+	+	+
3FM-16 (dioxo hydroxy-)	+	-	-
3FM-17 (oxo carboxylic acid-)	+	-	-
3FM-18 (oxo dihydroxy-)	+	-	-
3FM-19 (oxo dihydroxy-)	+	-	-
3FM-20 (trihydroxy-)	+	-	-
3FM-21 (hydroxy glucuronide-)	+	-	-
3FM-22 (hydroxy glucuronide-)	+	-	-
4-Fluoroprolintane	+	+	+
4FM-1 (*N,N-bis*-dealkyl-)	+	-	+
4FM-2 (*N,N-bis*-dealkyl hydroxy-)	+	-	-
4FM-3 (oxo-)	-	-	+
4FM-4 (oxo-)	+	+	+
4FM-5 (hydroxy-)	-	+	+
4FM-6 (hydroxy-)	+	-	-
4FM-7 (hydroxy-)	-	+	+

4FM-8 (*N*-oxide)	+	-	+
4FM-9 (oxo hydroxy-)	+	-	-
4FM-10 (oxo hydroxy-)	+	-	-
4FM-11 (oxo hydroxy-)	+	+	+
4FM-12 (oxo hydroxy-)	+	+	+
4FM-13 (dihydroxy-)	+	-	-
4FM-14 (dihydroxy-)	+	+	+
4FM-15 (oxo carboxylic acid-)	+	-	-
4FM-16 (oxo dihydroxy-)	+	-	-
4FM-17 (hydroxy methoxy-)	+	-	-
4FM-18 (trihydroxy-)	+	-	-
4FM-19 (trihydroxy-)	+	-	-
4FM-20 (*N,N-bis*-dealkyl hydroxy glucuronide-)	+	-	-
4FM-21 (hydroxy glucuronide-)	+	-	-
4FM-22 (dihydroxy glucuronide-)	+	-	-
4FM-23 (dihydroxy glucuronide-)	+	-	-
4FM-24 (dihydroxy glucuronide-)	+	-	-
